# Ruthenocenoporphyrinoids—π-Conjugation Transmitted across 1,3-Substituted Ruthenocene

**DOI:** 10.1021/acs.inorgchem.5c00470

**Published:** 2025-04-10

**Authors:** Anna Berlicka, Aleksandra Walczak, Michał J. Białek, Katarzyna Ślepokura, Piotr J. Chmielewski, Lechosław Latos-Grażyński

**Affiliations:** Department of Chemistry, University of Wrocław, 14 F. Joliot-Curie, 50-383 Wrocław, Poland

## Abstract

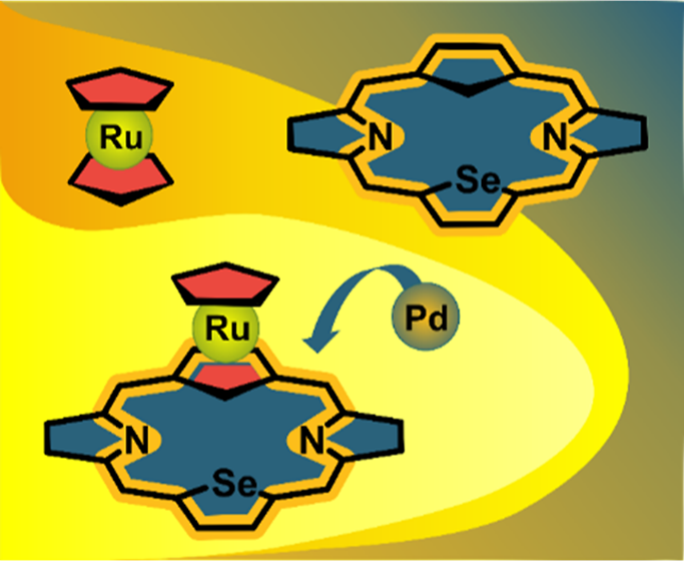

Synthesis of ruthenocenoporphyrinoids, wherein the [RuCp*]^+^ moiety coordinates to the cyclopentadienyl π-surface of the 21-carba-23-selenaporphyrin macrocyclic platform, has been developed. The specific electronic ruthenocene-macrocycle communication is observed. The macrocyclic ring current is maintained despite the strong π-conjugation in the cyclopentadienyl ring of the ruthenocene fragment. The theoretical data are consistent with the magnetic properties reflected by ^1^H NMR spectra. DFT-optimized molecular models were used to evaluate the NICS 2D maps and EDDB plots. These data gave an insight into the aromaticity and effectiveness of π-conjugation across 1,3-substituted ruthenocene in the obtained hybrid molecules.

## Abstract 2

The coordination of the [RuCp*]^+^ moiety to the cyclopentadienyl π-surface of 21-carba-23-selenaporphyrin or its palladium(II) complex resulted in the formation of original molecular hybrids incorporating 1,3-ruthenocene fragments. The NICS 2D maps and EDDB plots visualize the aromaticity and π-conjugation in the ruthenocenoselenaporphyrin.

## Introduction

Porphyrins and their analogues are excellent scaffolds for advanced investigation of the macrocyclic π aromaticity. The aromaticity of various porphyrinoid systems has been extensively investigated.^1^ Several factors, such as the molecular framework’s topology, the molecule’s conformation, metal coordination in the inner core, and tautomerism, can determine their π-electron conjugation.

**Figure 1:**
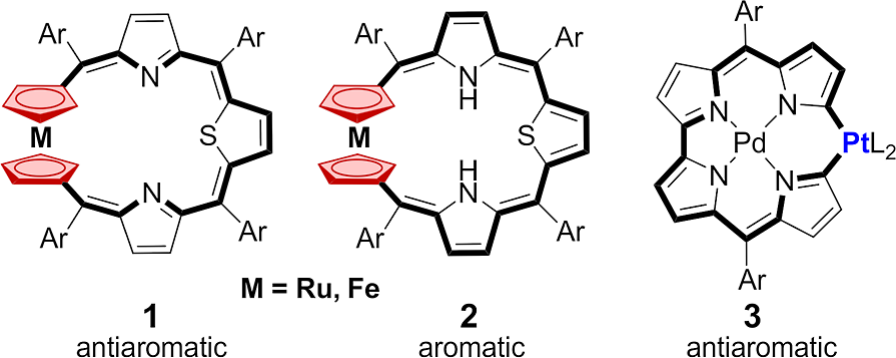
π-Delocalization through metallocenes (**1**, **2**) and the metal center (**3**).

The macrocycles combining the structural features of *ansa*-metallocenes and heteroporphyrin were obtained. The reported ferroceno- and ruthenocenoporphyrinoids provide evidence for direct transmission of π-electron conjugation across a d-electron ferrocene and ruthenocene, respectively (**1**, **2**; Figure 1).^2,3^ In the case of ruthenocenothiaporphyrin and ruthenocenoporphyrin, the corresponding macrocyclic antiaromaticity and aromaticity observed for the same number of π electrons were controlled by the mutual orientation of the cyclopentadienyl (Cp) rings.^3^ Recently, the effective π-conjugation through d-orbitals of the metal center, resulting in the antiaromatic character of the platinacorrole complex, has also been documented (**3**, Figure 1).^4^

**Figure 2:**
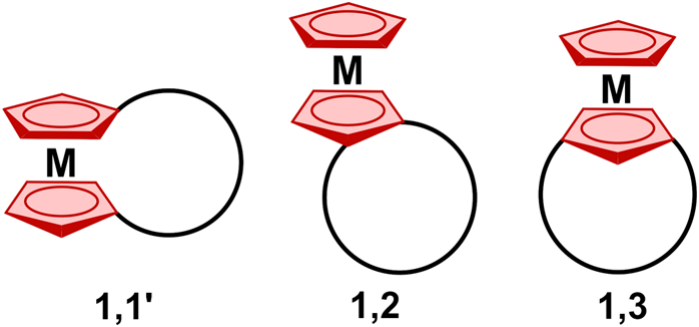
Feasible motifs of metallocene incorporation into a macrocyclic structure.

The metallocene unit can be incorporated into the macrocyclic platform in three modes: 1,1′; 1,2; and 1,3, as shown in Figure 2. To date, several bridged 1,1′-metallocene macrocycles have been reported. Except for a few examples involving porphyrinoids **1** and **2**,^2,3,5^ macrocyclic π-conjugation is impossible due to structural and/or conformational features.^6^ On the other hand, metallocenes that are 1,2- and 1,3-disubstituted and incorporated into a cyclic framework (Figure 2) are exceedingly uncommon.^7^

**Figure 3:**
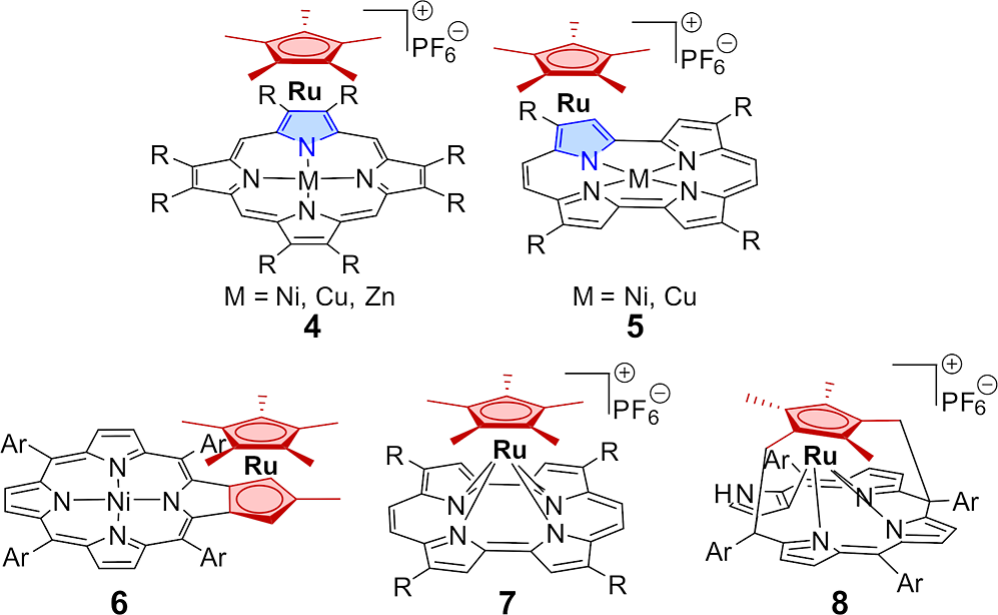
Examples of the coordination motifs of [RuCp*]^+^ to porphyrinoids.

The incorporation of metallocene subunits at the 1,2 or 1,3 positions into fully π-conjugated macrocycles is of fundamental importance in determining the overall electronic properties of the created hybrid molecules.^8,9^ The recent approach of direct metal π complexation to various oligopyrrolic ligands has been shown to modulate their spectroscopic, redox, optical, and electronic properties. In this approach, a direct coordination of [RuCp]^+^, [RuCp*]^+^ (Cp*, pentamethylcyclopentadienyl), or [Ru(*p*-cymene)]^+^ fragment to the pyrrole π-surface of π-conjugated porphyrins (**4**),^10^ porphycenes (**5**),^11^ and hexaphyrins^12^ has been investigated (Figure 3). In these complexes with a “fused” 1,3-azaruthenocene moiety (**4**, **5**; Figure 3), strong electronic azaruthenocene-macrocycle communication was observed.^10,11^ The synthesis of β,β′-fused cyclopentenylporphyrins allowed the introduction of a metallocene at the macrocyclic periphery (**6**, Figure 3).^13^

A different coordination motif of pentamethylcyclopentadienyl ruthenium cation is represented by “sitting-atop” semisandwich complexes of porphycene **7**,^11^ N-fused porphyrin,^14^ and N-confused porphyrin **8**,^15^ in which a [RuCp*]^+^ fragment is accommodated in the central N_3_/N_4_ core of the macrocycle.

**Scheme 1:**
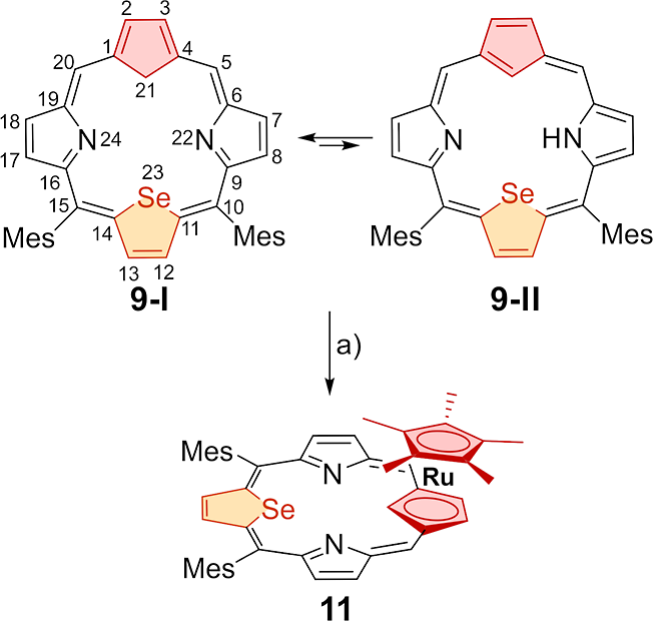
Synthesis of Ruthenocenoporphyrin **11** (Conditions a): [RuCp*(CH_3_CN)_3_][PF_6_] (0.5 equiv), CH_2_Cl_2_, 293 K

**Scheme 2:**
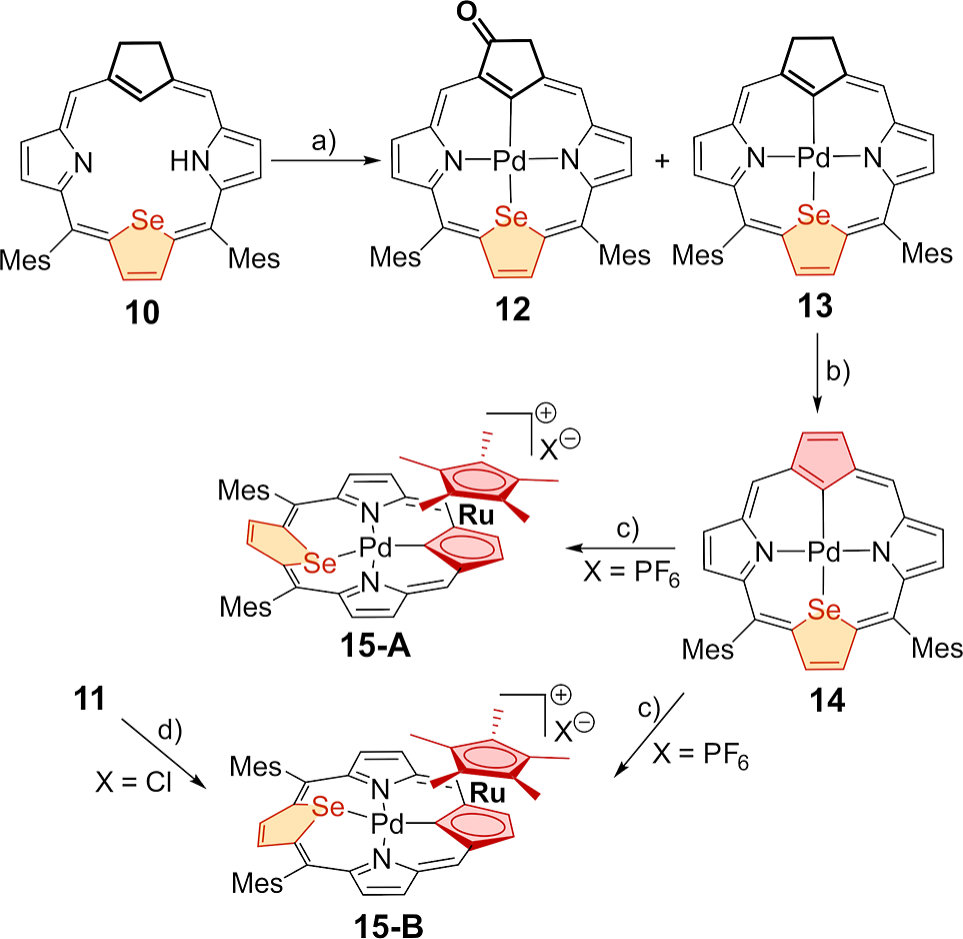
Synthesis of Palladium(II) Ruthenocenoporphyrin **15** Reaction conditions (a): PdCl_2_ (10 equiv), K_2_CO_3_, DMF, reflux, 10 min; (b): DDQ, CDCl_3_ (NMR titration), (c): [RuCp*(CH_3_CN)_3_][PF_6_] (1.1 equiv), CH_2_Cl_2_, 293 K, and (d): PdCl_2_ (10 equiv), K_2_CO_3_, CHCl_3_/CH_3_CN, reflux, 10 min.

Based on the above investigations and a recent study of 21-carbaselenaporphyrinoids **9** and **10** (Schemes 1 and 2),^16^ it is assumed that 21-carba-23-selenaporphyrin **9** can serve as a perfect macrocyclic π-conjugated platform for the coordination to a Cp ruthenium fragment, leading to the formation of the 1,3-metallacenoporphyrin hybrid (Figure 2). In contrast to other 21-carbaheteroporphyrins,^17,18^ 21-carba-23-selenaporphyrin exists predominantly as a tautomer **9-I** with the tetrahedral C(21) carbon atom (Scheme 1).^16^ Importantly, 1,3-metallocenoporphyrin was postulated as an intermediate complex in the synthesis of *meso*-tetraaryl-21-carbaporphyrin.^19^

## Results and Discussion

Herein, we report the synthesis and characterization of ruthenocenoselenaporphyrin **11** and its palladium complex **15**. Importantly, these hybrid carbaporphyrin-ruthenocene molecules indicate the direct transmission of π-electron conjugation across the 1,3-ruthenocene bridge. Significantly, the macrocyclic aromaticity is influenced by protonation or metal coordination in the metallocenoporphyrin cavity.

Treatment of 21-carbaselenaporphyrin **9** or its palladium complex **14** with [RuCp*(CH_3_CN)_3_][PF_6_] in dichloromethane at 293 K resulted in the η^5^-coordination of a [RuCp*]^+^ fragment to the cyclopentadiene ring of **9** or **14**. This led to the formation of ruthenocenoporphyrin **11** (Scheme 1) or bimetallic palladium(II) ruthenocenoporphyrin **15** (two stereoisomers, Scheme 2) in yields of 62% and 96%, respectively. Palladium(II) 21-carbaselenaporphyrin **14** was synthesized on an NMR scale by attentive titration of the corresponding 21-carbaselenachlorin complex **13** with a saturated solution of DDQ in CDCl_3_ (Scheme 2). Palladium(II) complexes **12** and **13** were obtained in the reaction of 21-carbaselenachlorin **10** with PdCl_2_ in DMF at reflux with 5% and 39% yields, respectively (Scheme 2; see the Supporting Information). The selective formation of complex **13** with a yield of 58% was observed in a mixture of CHCl_3_/CH_3_CN as a solvent.

**Figure 4:**
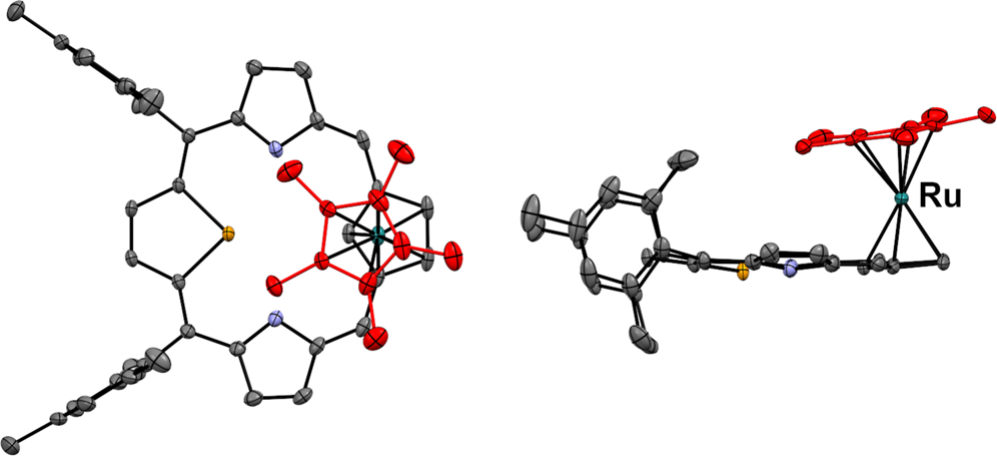
Molecular structure of **11** is presented in two perspectives: a top view (left) and a side view (right). Atoms of the pentamethylcyclopentadienyl ring are shown in red. The displacement ellipsoids indicate a 30% probability. Hydrogen atoms, the disorder of Cp*, and mesityl groups have been omitted for clarity.

The coordination of the [RuCp*]^+^ moiety to the cyclopentadiene ring of **9** has been confirmed by X-ray diffraction studies (Figure 4). The macrocyclic ring adopts a nearly planar conformation. The planes of the macrocyclic Cp and pentamethylcyclopentadienyl rings are almost parallel (the dihedral angle equals 4.5°). The distances between the Cp*, Cp ring planes, and the ruthenium ion are 1.81 and 1.83 Å, respectively. These values are slightly longer than those observed in the corresponding RuCp*-polyarene complexes (ca. 1.80–1.81 Å)^20^ and RuCp* complexes of β-substituted porphyrin and porphycene (ca. 1.78–1.79 Å).^10,11^

**Figure 5:**
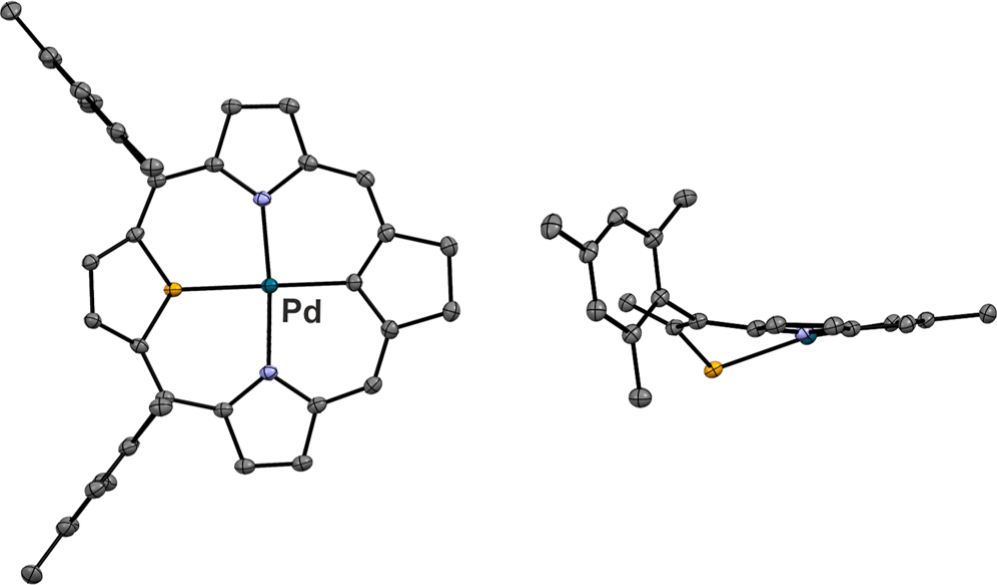
Molecular structure of **13** is presented in two perspectives: a top view (left) and a side view (right). The displacement ellipsoids indicate a 30% probability. Hydrogen atoms have been omitted for clarity.

The molecular structure of **13** exhibits a significant deviation from planarity (Figure 5). This is due to the selenophene ring being tilted at an angle of 38.0° to the macrocyclic plane, defined by four *meso*-carbons. This distortion allows pyramidal side-on coordination of the palladium(II) ion.

**Figure 6:**
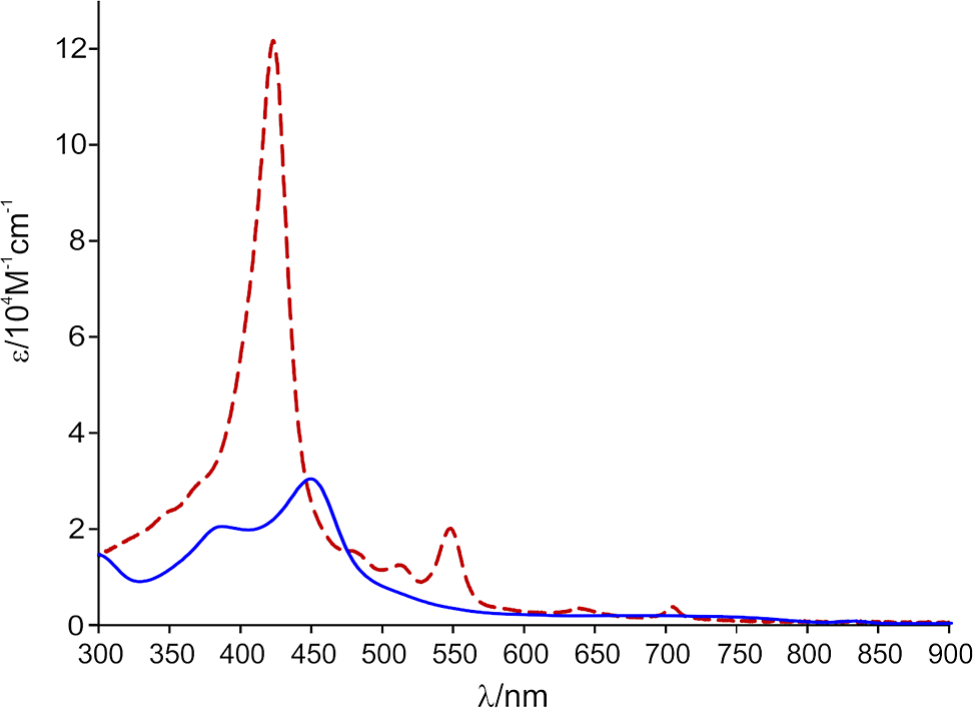
UV–vis spectra of **9** (dashed red) and **11** (solid blue) in dichloromethane.

The electronic spectrum of ruthenocenoselenaporphyrin **11** (blue in Figure 6) displays four bands at 299, 385, 449, and 692 nm. The Soret band at 449 nm is bathochromically shifted compared to 21-carbaselenaporphyrin **9** (423 nm, red in Figure 6).^16^ The electronic absorption spectrum of **14** shows eight bands from 350 to 790 nm, while the palladium(II) of ruthenocenoselenaporphyrin **15** (mixture of two isomers) demonstrates an entirely different spectrum with three bands at 302, 404, and 497 nm (Figure S31).

**Figure 7:**
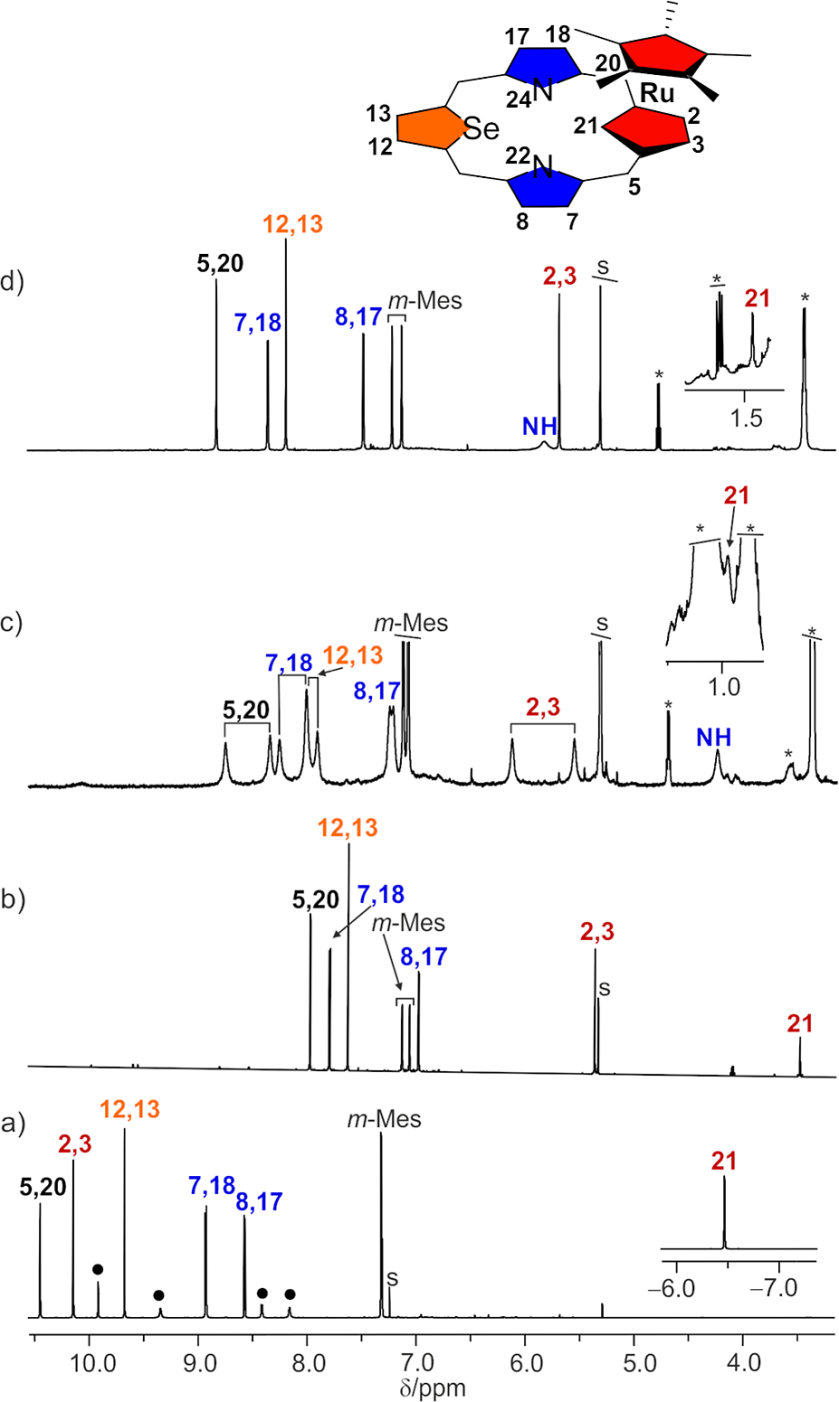
^1^H NMR spectra for compounds (a) **9-I** (**9-II** shown as black circles, 300 K, CDCl_3_), (b) **11** (300 K, CD_2_Cl_2_), (c) **11-H**^**+**^ (190 K, CD_2_Cl_2_), and (d) **11-H**_**2**_^**2+**^ (300 K, CD_2_Cl_2_). *o*,*p*-Methyl range is omitted (see the Supporting Information for details). The inset at trace (d) presents the numbering of proton positions.

The ^1^H NMR spectrum of the ruthenium(II) π-complex of 21-carba-23-selenaporphyrin **11** indicates its *C*_s_ symmetry (Figure 7b). The resonances of the external macrocyclic ring protons are relocated upfield compared to those of the parent macrocycle **9** (Figure 7a). The external *meso*-H and selenophene H(12,13) are detected at 7.96 and 7.61 ppm, respectively. The β-pyrrole protons H(7,18) and H(8,17) produce two doublets observed at 7.78 and 6.97 ppm, correspondingly. The outer protons H(2,3) of cyclopentadiene give a doublet at 5.35 ppm, resulting from scalar coupling with the inner H(21). The inner H(21) signal shows a significant relocation to 3.47 ppm, in contrast to **9** (**9-I**: −6.53 ppm; **9-II**: −4.17 ppm),^16^ however similar to the chemical shifts observed for ruthenocene (δ = 4.52 ppm).^21^ The positions of the signals indicate a weakening of the diatropic ring current due to the η^5^–π bonding of the [RuCp*]^+^ fragment to the cyclopentadiene ring of **9**. Notably, the ^13^C chemical shifts detected for the cyclopentadiene carbon atoms C(1,4), C(2,3), and C(21) of **11** at 89.1, 82.4, and 77.3 ppm, respectively, clearly indicate coordination of the carbaselenaporphyrin platform to the [RuCp*]^+^ moiety (for ruthenocene and its substituted derivatives δ = 70.0–91.0 ppm).^22^

**Scheme 3:**
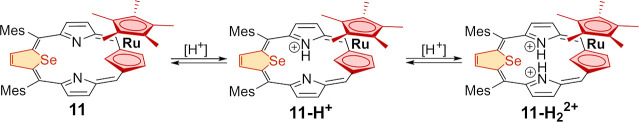
Protonation of **11**

Gradual acidification of compound **11** with a fluoroboric acid diethyl ether complex or trifluoroacetic acid solutions resulted in deep orange monocation **11-H**^**+**^ formation in the first stage, followed by red-brown dication **11-H**_**2**_^**2+**^ (Scheme 3).

The protonation of both pyrrole nitrogen atoms resulted in the relocation of the H(2,3) and H(21) ^1^H NMR signals to 5.87 and 1.76 ppm in **11-H**_**2**_^**2+**^, respectively (Figure 7d). The signals of meso, selenophene, and pyrrole hydrogens show downfield transfer to 8.66, 8.03, 8.19, and 7.32 ppm, correspondingly. Symmetry lowering of **11-H**^**+**^ due to the protonation of one pyrrole nitrogen atom is reflected in the ^1^H NMR spectrum (Figure 7c). The UV–vis electronic spectra of **11-H**^**+**^ and **11-H**_**2**_^**2+**^ are shown in the Supporting Information (Figure S29).

**Figure 8:**
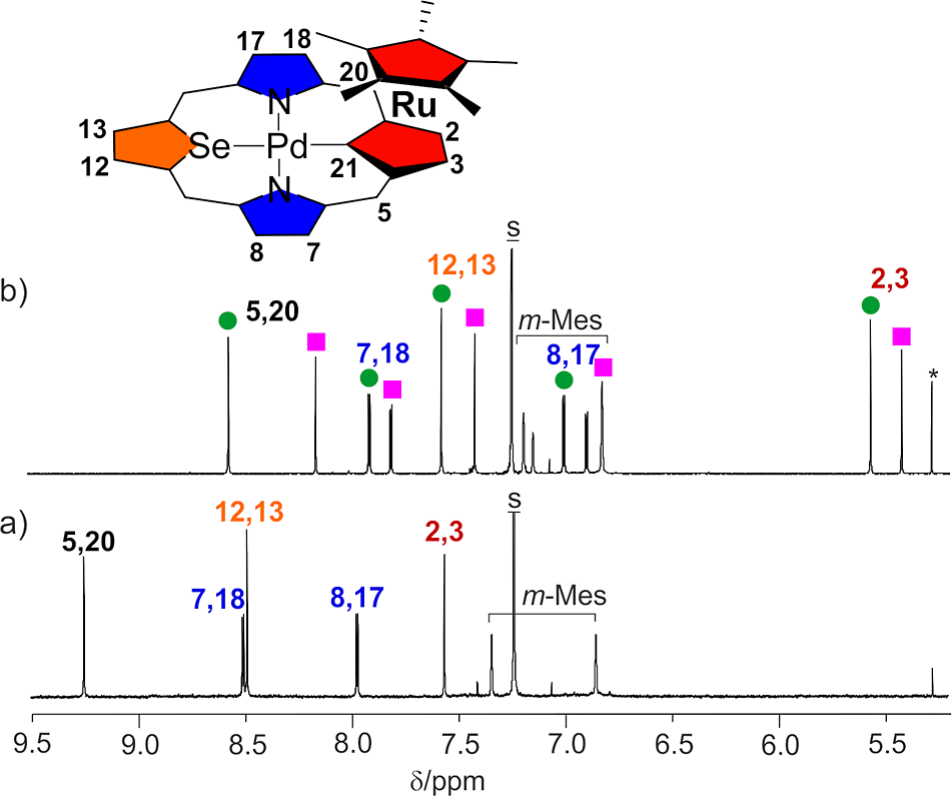
^1^H NMR spectra for compounds (a) **14** and (b) two isomers of **15** (**15-A** denoted as green circles, **15-B** denoted as pink squares; 300 K, CDCl_3_). *o*,*p*-Methyl range is omitted (see the Supporting Information for details).

The ^1^H NMR spectra of the palladium(II) complexes of 21-carbaselenaporphyrinoids **12**, **13**, and **14** exhibit resonances at positions consistent with their aromatic structures (Figures 8a, S10, and S15). The bonding of the [RuCp*]^+^ cation to the cyclopentadiene ring of **14** resulted in the formation of two isomers of a bimetallic complex (**15-A** and **15-B**, Scheme 2). These isomers are differentiated by the orientation of the selenophene ring, which is tilted toward or against the [RuCp*]^+^ moiety in **15-A** and **15-B**, respectively. At 300 K, both stereoisomers of **15** exhibit well-separated sets of ^1^H NMR resonances with similar patterns (Figure 8b). Each set of signals was assigned to a specific isomer based on the GIAO-calculated ^1^H NMR spectra for the density functional theory (DFT)-optimized structures (Figures S23–S45). The ratio of **15-A** to **15-B** isomers, determined through analysis of ^1^H NMR spectra, is 1:0.7 (300 K). The external meso [H(5,20)], pyrrole [H(7,18); H(8,17)], selenophene [H(12,13)], and cyclopentadiene [H(2,3)] protons exhibit resonances that are shifted upfield relative to the parent **14** (Figure 8a) indicating a weakening of the aromatic ring current due to coordination of the [RuCp*]^+^ moiety.

A rare example of stereoselective porphyrin metalation^23^ was encountered once ruthenocenoporphyrin **11** was engaged as a macrocyclic ligand. Thus, the reaction with PdCl_2_ resulted in the selective formation of a single stereoisomer of **15**, presumably **15-B**, due to the steric hindrance imparted by the [RuCp*]^+^ fragment (Scheme 2). A feasible conversion to the counterpart was not detected in CDCl_3_ (300 K) over 48 h, as followed by ^1^H NMR spectroscopy. The ^77^Se chemical shift values of the two isomers of **15** are observed in a similar region at 354 and 346 ppm for **15-A** and **15-B**, respectively. These values are shifted upfield compared to ligand **11**, which demonstrates a ^77^Se chemical shift at 598 ppm. This is similar to the value observed for selenophene (δ = 605 ppm).^24^

A DFT study was conducted for complexes **11** and **15** to elucidate the electronic structure of the 21-carbaselenaporphyrin and its palladium(II) complex following π-coordination of the Cp ring to the [RuCp*]^+^ moiety.

**Figure 9:**
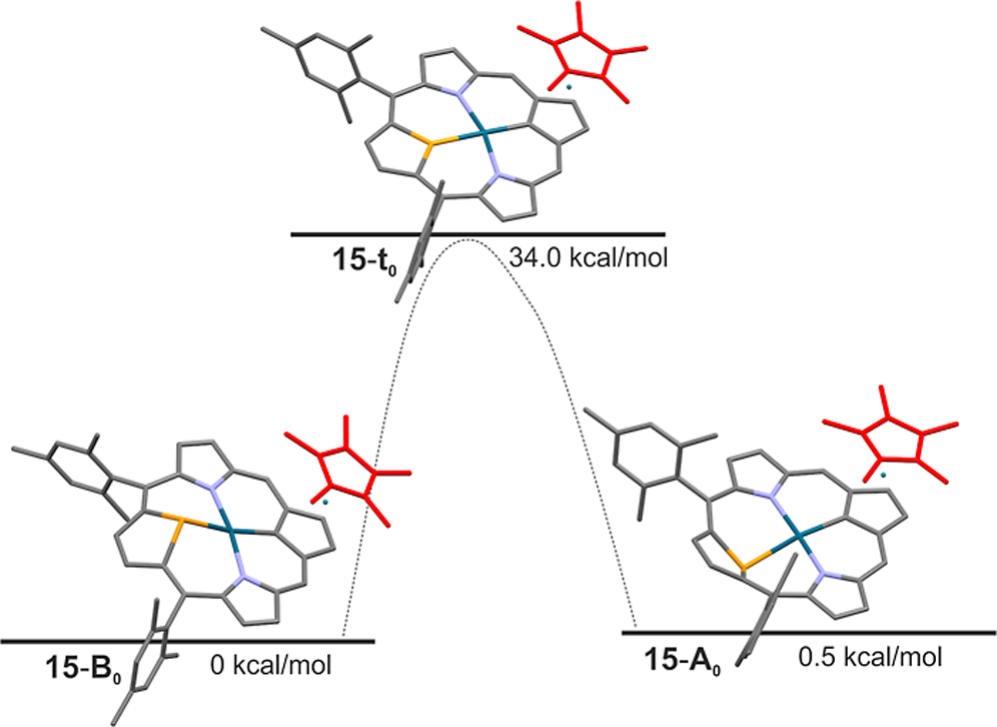
Energy diagram for conformational interconversion of **15**. Provided are calculated relative energy differences.

A comparison of the crystal and DFT-optimized structures of **11** reveals a high degree of agreement. This is evidenced by a detailed comparison of the bond lengths, as shown in Figures S38 and S39. Both isomers of **15** were subjected to DFT studies, and the final geometries are presented in Figure 9. The triheterocyclic braces involving two pyrrole and Cp rings demonstrate a planar geometry. In contrast, selenophene is tilted away from the macrocyclic plane. The geometry of the transient species **15-t**_**0**_ with the selenophene ring located in the carbaporphyrinic plane was also considered. The selenophene tilt angles from the four *meso*-carbon atoms plane equal −37.2° (**15-A**_**0**_) and 35.3° (**15-B**_**0**_), for the transient species **15-t**_**0**_, is close to 0°. The energy difference between isomers **15-A** and **15-B** is negligible (Δ*E* = 0.5 kcal/mol). The transient form **15-t**_**0**_, with the selenophene ring in the macrocyclic plane, has an energy much higher than that of **15-B**_**0**_, with a barrier of 34.0 kcal/mol.

**Scheme 4:**
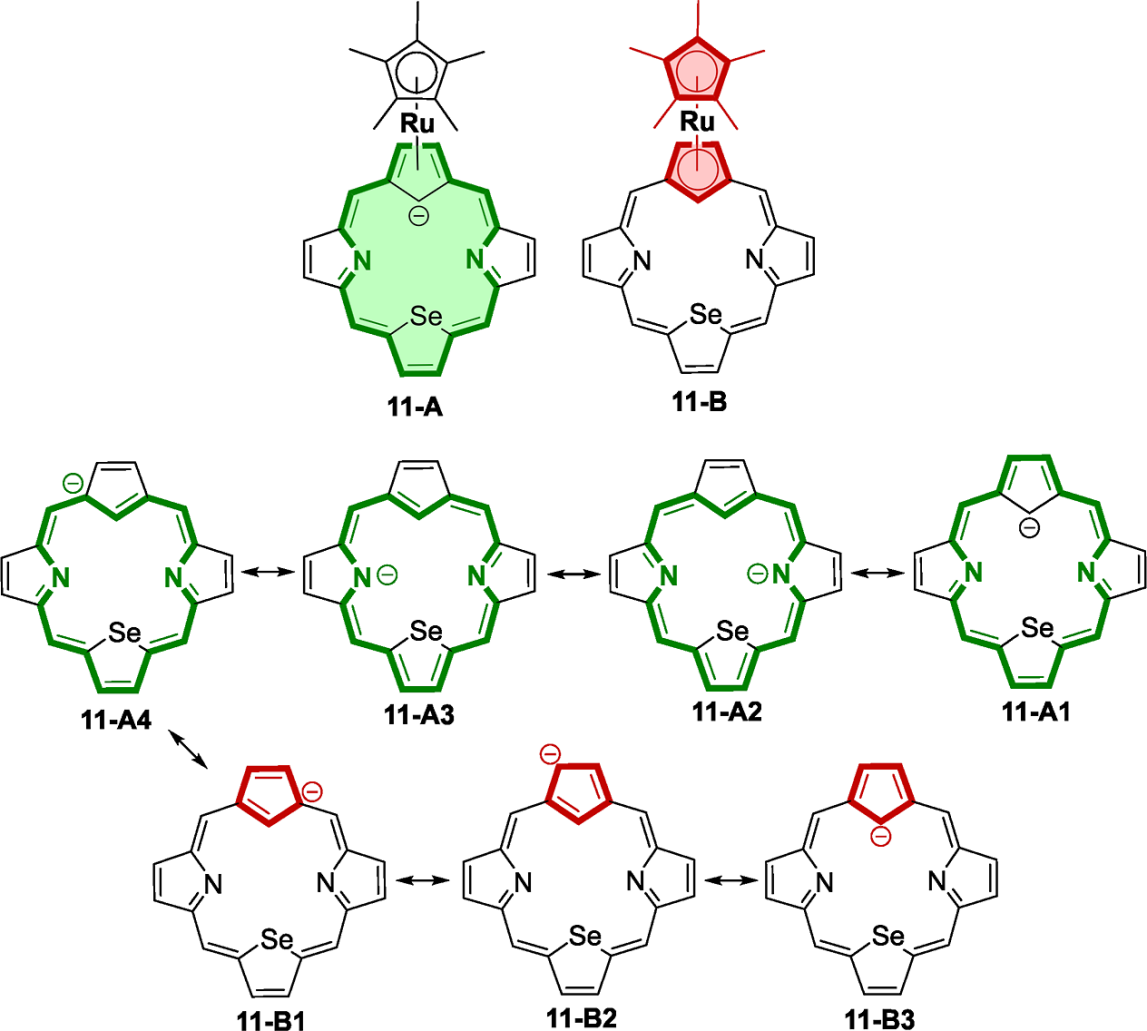
Resonance Structures of Anion **11**

The aromaticity of the carbaselenaporphyrin unit has been analyzed in detail after the coordination of the [RuCp*]^+^ cation to the cyclopentadiene ring in **11** and **15**. The significant resonance contributors for the carbaselenapophyrin anion in **11** are illustrated in Scheme 4. These resonance contributors involve the delocalization of 18 π-electrons within the macrocycle (**11-A**) or 6 π-electrons (**11-B**) within a Cp ring. The magnetic properties of π-complex **11** can be considered as a result of a hard-fought struggle for the supremacy of global vs local aromatic delocalization encompassed in resonance structures **11-A** vs **11-B**.

The comparison of bond lengths in the DFT-optimized geometries of the selenatripyrrin fragments of **11** and **9-I** indicates a strong bond equalization in the aromatic 21-carbaselena-porphyrin **9-I** (Figure S40). Conversely, a significant alternation in bond lengths is observed in **11** after coordination of the [RuCp*]^+^ fragment due to a less efficient π delocalization effect.

**Figure 10:**
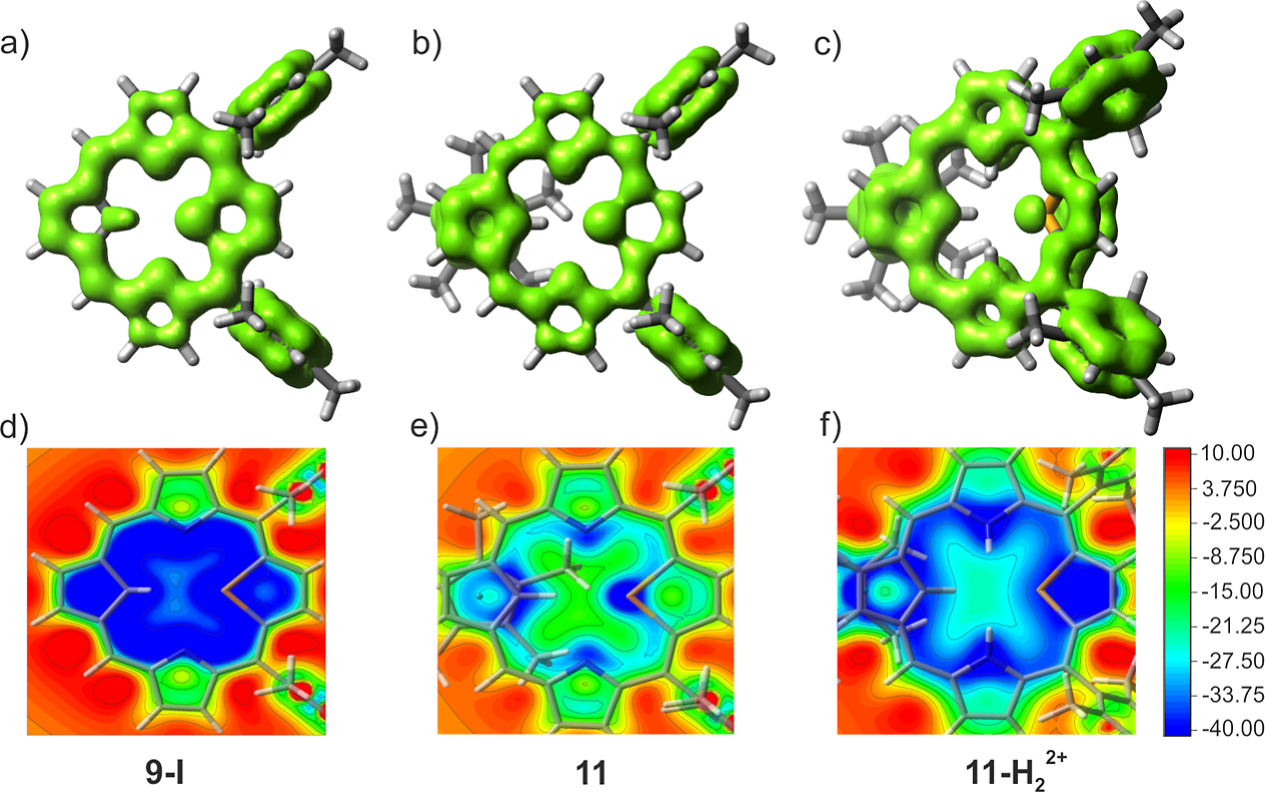
EDDB plots (a–c) and NICS(1)zz 2D maps (d–f) of 21-carba-23-selenaporphyrin **9-I** (a,d), ruthenium(II) π complex **11** (b,e), and **11-H**_**2**_^**2+**^ (c,f). In EDDB plots, the localized and delocalized cyclic π-conjugation is shown with the green surface with an isovalue of 0.014, while NICS maps are estimated 1 Å above the mean plane. RuCp* located behind the carbaporphyrinic plane.

The electron density of delocalized bonds (EDDB)^25^ was employed to investigate cyclic π delocalization across conjugated bonds in **11** and **15**, with **9** and **14** serving as comparative controls (Figures 10a–c and S41). The EDDB isocontour maps of **11** and **11-H**_**2**_^**2+**^ reveal the presence of localized π-conjugation on the Cp ring, which does not disrupt the π delocalization throughout the carbaporphyrin structure (Figure 10b,c). It is observed that effective π-conjugation occurs through the 1,3-substituted ruthenocene moiety.

The aromaticity of ruthenocenoselenaporphyrin **11** and its dication **11-H**_**2**_^**2+**^ was further analyzed using nucleus-independent chemical shift (NICS) calculations.^26^ The analysis of the NICS two-dimensional (2D) map of **11** revealed negative values within the inner cavity of the macrocyclic ring (Figure 10e). However, these values were significantly less negative than those observed for control **9-I** (Figure 10d). After protonation in **11-H**_**2**_^**2+**^, the aromaticity features became more visible (Figure 10f). The NICS(0) values were calculated for molecules **11** and **11-H**_**2**_^**2+**^ at the centers of the macrocycles (Figure S37). After binding of the [RuCp*]^+^ moiety to **9**, the corresponding NICS(0) values are noticeably less negative −8.5 in **11** and −10.0 in **11-H**_**2**_^**2+**^ compared to **9** (−16.7 in **9-I**, −14.8 in **9-II**).

These observations are consistent with the ^1^H NMR spectroscopic patterns. The differences in the efficiency of π-delocalization in 21-carba-23-selenaporphyrin **9-I**, ruthenocenoselenaporphyrin **11**, and its dication **11-H**_**2**_^**2+**^ are readily apparent in NICS 2D maps. The EDDB contour plots and NICS 2D maps demonstrate π-delocalization across the 1,3-ruthenocene fragment. Analogous observations have been made for the bimetallic complex **15** and the control palladium complex **14** (Figure S41). However, in this particular case, the strong deviation of the selenophene ring from the macrocyclic plane defined by the *meso*-carbon atoms contributes to a decrease in the π-conjugation efficiency.

^1^H and ^13^C NMR chemical shifts calculated for **11**, **11-H**_**2**_^**2+**^, **15-A**, and **15-B** are presented in the Supporting Information (Tables S3–S6). The theoretical results correlate well with the experimental shifts obtained from ^1^H and ^13^C NMR spectroscopy, except for the upfield relocated inner proton H(21) in **11** and **11-H**_**2**_^**2+**^ (Figures S42 and S43) and C(21) in **15** (Figures S44 and S45).

## Conclusions

In conclusion, the π coordination of the [RuCp*]^+^ fragment to the Cp ring incorporated into 21-carba-23-selenaporphyrin was developed. Consequently, the incorporation of ruthenocene subunits at the 1,3-positions into fully π-delocalized macrocycle—21-carbaselenaporphyrin led to the formation of a hybrid molecule—aromatic ruthenocenoselenaporphyrin. The evidence for direct transmission of π-electron conjugation across a d-electron ferrocene or ruthenocene was previously provided by the exploration of ferroceno- and ruthenocenoporphyrinoids incorporating 1,1′-metallocene. The present work reveals a hitherto unknown feature of metallocenoporphyrinoids. The relevant ^1^H NMR spectra of ruthenocenoselenaporphyrin and its protonated forms confirmed the presence of a macrocyclic aromaticity. The aromaticity and π-delocalization patterns were visualized using EDDB plots and NICS 2D maps. Remarkably, ruthenocenoselenaporphyrin acts as a monoanionic organometallic macrocyclic ligand, as evidenced by the formation of a heterometallic ruthenium(II)–palladium(II) derivative, providing the rear example of M–C σ-coordination of the metallocene unit in the macrocyclic surroundings.

Further investigation into macrocyclic organometallic chemistry may entail the synthesis of analogous metallocenoporphyrins, utilizing a range of metallocenes and 21-carba-23-heteroporphyrins as fundamental building subunits. The feasible interplay between two metal cations locked in the firm molecular architecture offers a novel route for organometallic and metallocene-focused investigations, combining the benefits of metallocene and carbaporphyrin.

## Experimental Section

### Solvents and Reagents

If not indicated differently, all solvents (chloroform, dichloromethane, ethyl acetate, *n*-hexane, and methanol) were used without purification. Dry dichloromethane was used as received (sure/seal system, stored in the glovebox). CDCl_3_ was prepared directly before use by running it through a basic alumina column. Reagents not listed here were used as received. 21-Carba-23-selenaporphyrinoids **1** and **2** were synthesized as described in the literature.^16^ The ruthenium π complexes **11** and **15** were synthesized according to the established procedures, with modifications to the reaction conditions (see below).^10,11^

### Synthesis of 11

9.4 mg (0.015 mmol) of **9** was placed in a 5 mL vial and introduced into the glovebox. The 4 mg (0.008 mmol) of [RuCp*(CH_3_CN)_3_](PF_6_) and 2 mL of dry CH_2_Cl_2_ were added. The mixture was stirred for 10 min and removed from the glovebox. The solvent was evaporated with nitrogen, and the residue was separated on a silica gel column (mesh 70–230) with CH_2_Cl_2_. Product **11** was identified in the first fraction. After subsequent chromatography (silica gel, mesh 70–230; CH_2_Cl_2_), compound **11** was eluted in the first fraction with a 62% yield (4.2 mg). An increase in the amount of [RuCp*(CH_3_CN)_3_](PF_6_) (1–2 equiv) has been observed to result in a reduction in the efficiency of the synthesis of compound **11**. **UV–vis** (CH_2_Cl_2_): λ_max_ (logε) = 299 (4.2), 385 (4.3), 449 (4.5), 692 nm (3.3). ^**1**^**H NMR** (600 MHz, CD_2_Cl_2_, 300 K): δ 7.96 (s, 2H, H5,20); 7.78 (d, ^3^*J* = 4.3 Hz, 2H, H7,18); 7.61 (s, 2H, H12,13); 7.12 (s, 2H, *m*-Mes); 7.05 (s, 2H, *m*-Mes); 6.97 (d, ^3^*J* = 4.3 Hz, 2H, H8,17); 5.35 (d, ^4^*J* = 1.2 Hz, 2H, H2,3); 3.47 (s, 1H, H21); 2.44 [s, 6H, *p*-CH_3_(Mes)]; 2.18 [s, 6H, *o*-CH_3_(Mes)]; 1.82 [s, 6H, *o*-CH_3_(Mes)]; 0.98 ppm [s, 15H, CH_3_(Cp*)]. ^**13**^**C NMR** (150.9 MHz, CD_2_Cl_2_, 300 K): δ 166.5; 153.7; 151.8; 138.3; 138.2; 137.7; 137.0 (C7,18); 136.52 (C12,13); 136.47; 134.9 (C5,20); 130.7; 128.5 [C(*m*-Mes), C(*m*′-Mes)]; 127.1 (C8,17); 89.1 (C1,4); 86.5; 82.4 (C2,3); 77.3 (C21); 21.5; 21.3; 20.7; 10.1 ppm [CH_3_(Cp*)]. ^**77**^**Se NMR** (CDCl_3_, 300 K, data from HMBC): δ 598 ppm. **HR-MS** (ESI): *m*/*z* calcd for C_49_H_49_N_2_RuSe^+^ [M + H]^+^, 847.2119; found, 847.2105.

### Synthesis of 12 and 13

Macrocycle **10** (12.8 mg, 0.021 mmol) and 5 mL of DMF were placed in a two-neck 25 mL flask. Nitrogen was bubbled through the solution for 10 min, and then K_2_CO_3_ (a few mg) and PdCl_2_ (37.2 mg, 0.21 mmol) were added, and the mixture was stirred in reflux under nitrogen for 10 min. The residue was separated on a silica gel column (mesh 70–230). Complex **13** was removed in the first fraction with CH_2_Cl_2_ as an eluant, while complex **12** was eluted with 1% MeOH in CH_2_Cl_2_ in the second fraction. After subsequent chromatography of both complexes **12** and **13** (basic alumina, Brockmann III grade; 50% hexane in CH2Cl2), the compounds were obtained in 5% (0.8 mg) and 39% yields (5.8 mg), respectively. The selective formation of complex **13** was observed with a yield of 58% (14.1 mg) when the reaction of **10** (21 mg, 0.034 mmol) with 10 equiv of PdCl_2_ in the presence of K_2_CO_3_ proceeded in a mixture of CHCl_3_/CH_3_CN (16 mL, 1:1, *V*:*V*) at reflux (reaction time 0.5 h).

1**2: UV–vis** (CH_2_Cl_2_): λ_max_ (logε) = 348 (4.3), 489 (4.7), 714 nm (4.0). ^**1**^**H NMR** (600 MHz, CDCl_3_, 300 K): δ 10.10 (s, 1H, H5); 9.75 (s, 1H, H20); 9.06 (d, ^3^*J* = 4.6 Hz, 1H, H7); 8.98 (d, ^3^*J* = 4.6 Hz, 1H, H18); 8.88 (d, ^3^*J* = 5.2 Hz, 1H, H12); 8.81 (d, ^3^*J* = 5.2 Hz, 1H, H13); 8.60 (d, ^3^*J* = 4.6 Hz, 1H, H17); 8.55 (d, ^3^*J* = 5.2 Hz, 1H, H8); 7.45 (s, 2H, *m*-Mes); 6.95 (s, 1H, *m*-Mes); 6.94 (s, 1H, *m*-Mes); 5.40 (d, ^2^*J* = 20.0 Hz, 1H, H3′); 5.20 (d, ^2^*J* = 20.0 Hz,1H, H3); 2.94 [s, 3H, *o*-CH_3_Mes)]; 2.93 [s, 3H, *o*-CH_3_(Mes)]; 2.55 [s, 3H, *p*-CH_3_(Mes)]; 2.54 [s, 3H, *p*-CH_3_(Mes)]; 0.39 [s, 3H, *o*-CH_3_(Mes)]; 0.38 ppm [s, 3H, *o*-CH_3_(Mes)]. ^**13**^**C NMR** (CDCl_3_, 300 K, data from HSQC and HMBC): δ 205.2 (C2); 150.2 (C14); 148.0 (C11); 138.0 (C13); 136.6 (C12); 134,4 (C18); 132.1 (C7); 130.9 (C17); 130.0 (C8); 128.2 (*m*-Mes); 119.4 (C5); 114.5 (C20); 47.5 (C3); 22.2 [*o*-CH_3_(Mes)]; 21.3 [*p*-CH_3_(Mes)]; 19.0 [*o*-CH_3_(Mes)] ppm. **HR-MS** (ESI): *m*/*z* calcd for C_39_H_32_N_2_OPdSe^+^ [M]^+^, 730.0728; found, 730.0756.

**13: UV–vis** (CH_2_Cl_2_): λ_max_ (logε) = 335 (4.4), 386 (4.2), 487 (4.8), 718 nm (4.1). ^**1**^**H NMR** (600 MHz, CDCl_3_, 300 K): δ 9.62 (s, 2H, H5,20); 8.93 (d, ^3^*J* = 4.6 Hz, 2H, H7,18); 8.75 (s, 2H, H12,13); 8.62 (d, ^3^*J* = 4.6 Hz, 2H, H8,17); 7.43 (s, 2H, *m*-Mes); 6.96 (s, 2H, *m*-Mes); 5.02 (dd, ^2^*J* = 16.2 Hz, ^3^*J* = 3.5 Hz, 2H, H2,3); 4.80 (dd, ^2^*J* = 16.2 Hz, ^3^*J* = 3.5 Hz, 2H, H2′,3′); 2.89 [s, 6H, *o*-CH_3_(Mes)]; 2.56 [s, 6H, *p*-CH_3_(Mes)]; 0.46 ppm [s, 6H, *o*-CH_3_(Mes)]. ^**13**^**C NMR** (150.9 MHz, CDCl_3_, 300 K): δ 150.5 (C6,19); 148.3 (C11,14); 147.5 (C1,4); 144.6 (C9,16); 142.7 (C21); 139.1; 139.0; 138.1; 137.8; 135.0 (C12,13); 131.6 (C7,18); 130.0 (C8,17); 128.2 (*m*-Mes); 128.0 (*m*-Mes); 115.3 (C5,20); 35.3 (C2,3); 22.3 [*o*-CH_3_(Mes)]; 21.4 [*p*-CH_3_(Mes)]; 19.1 ppm [*o*-CH_3_(Mes)]. **HR-MS** (ESI): *m*/*z* calcd for C_39_H_34_N_2_PdSe^+^ [M]^+^, 716.0935; found, 716.0981.

### Synthesis of 14

Complex **14** was prepared on an NMR scale. **13** (3.8 mg, 0.0053 mmol) was dissolved in CDCl_3_ and transferred to an NMR tube. The solution of **13** was titrated with a saturated solution of DDQ in CDCl_3_. The product was purified by column chromatography on a silica gel column (mesh 70–230) with CH_2_Cl_2_ as an eluant. Compound **14** was obtained in 55% (2.1 mg) yield. **UV–vis** (CH_2_Cl_2_): λ_max_ (logε) = 354 (4.6), 410 (4.5), 455 (4.5), 497 (4.3), 527 (4.3), 570 (4.3), 692 (3.9), 788 nm (3.7). ^**1**^**H NMR** (600 MHz, CDCl_3_, 300 K): δ 9.25 (s, 2H, H5,20); 8.50 (d, ^3^*J* = 4.5 Hz, 2H, H7,18); 8.49 (s, 2H, H12,13); 7.97 (d, ^3^*J* = 4.5 Hz, 2H, H8,17); 7.56 (s, 2H, H2,3); 7.34 (s, 2H, *m*-Mes); 6.85 (s, 2H, *m*-Mes); 2.90 [s, 6H, *o*-Me(Mes)]; 2.48 [s, 6H, *p*-CH_3_(Mes)]; 0.44 ppm [s, 6H, *o*-CH_3_(Mes)]. ^**13**^**C NMR** (150.9 MHz, CDCl_3_, 300 K): δ 153.1 (C6,19/C9,16); 148.2 (C11,14); 142.9 (C6,19/C9,16); 138.9; 138.7 (C12,13); 138.3; 138.0; 137.4 (C1,4); 136.7; 135.9 (C21); 135.1; 133.3 (C7,18); 132.9 (C2,3); 128.3 (*m*-Mes); 128.2 (*m*-Mes); 127.8 (C8,17); 126.9 (C5,20); 21.9 [*o*-CH_3_(Mes)]; 21.3 [*p*-CH_3_(Mes)]; 19.0 ppm [*o*-CH_3_(Mes)]. **HR-MS** (ESI): *m*/*z* calcd for C_39_H_32_N_2_PdSeCl^+^ [M + Cl]^+^, 749.0463; found, 749.0490.

### Synthesis of 15

3.9 mg (0.0055 mmol) portion of **14** was transferred to a 5 mL vial and placed in the glovebox. The 3.2 mg (0.0069 mmol) of [RuCp*(CH_3_CN)_3_](PF_6_) and 1.5 mL of dry CH_2_Cl_2_ were added. The mixture was stirred for 10 min and removed from the glovebox. The residue was separated on a silica gel column (mesh 70–230) without evaporation of the solvent. Product **15** was eluted in the second fraction with 5% ethyl acetate in CH_2_Cl_2_. **15** was obtained with a 96% (5 mg) yield. Finally, two **15** (**A** and **B**) stereoisomer resolutions were performed using HPLC on an achiral stationary phase analytical column (5u, silica gel, 25 × 0.46 cm) with 5% methanol in CH_2_Cl_2_ as the eluant. The isomer **15-B** was identified in the first fraction, while the isomer **15-A** was identified in the third fraction. The principal second fraction contained a mixture of two isomers. Complexes **15** are stable under anaerobic conditions. **UV–vis** for a mixture of two isomers of **15** (CH_2_Cl_2_): λ_max_ (logε) = 302 (4.2), 404 (4.2), and 497 nm (4.3). **HR-MS** (ESI): *m*/*z* calcd for C_49_H_47_ N_2_PdRuSe^+^ [M – PF_6_]^+^, 951.1010; found, 951.1053.

### **15-A:**^**1**^**H NMR**

(600 MHz, CDCl_3_, 300 K): δ 8.60 (s, 2H, H5,20); 7.93 (d, ^3^*J* = 4.8 Hz, 2H, H7,18); 7.62 (s, 2H, H12,13); 7.20 (s, 2H, *m*-Mes); 7.05 (d, ^3^*J* = 4.8 Hz, 2H, H8,17); 6.84 [s, 2H, *m*-(Mes)]; 5.60 (s, 2H, H2,3); 2.64 [s, 6H, *o*-CH_3_(Mes)]; 2.39 [s, 6H, *p*-CH_3_(Mes)]; 1.17 [s, 15H, CH_3_(Cp*)]; 1.00 ppm [s, 6H, *o*-CH_3_(Mes)]. ^**13**^**C NMR** (CDCl_3_, 300 K, data from HSQC and HMBC): δ 160.4 (C6,19/C9,16); 148.3 (C5,20); 144.8 (C6,19/C9,16); 143.7 (C11,14); 140.7 (C12,13); 137.3 (C7,18); 129.0 (H8,17); 128.8 (*m*-Mes); 128.6 (*m*-Mes); 103.8 (C21); 90.5 (Cp*); 89.0 (C1,4); 84.4 C(2,3); 21.0 [*o*,*p*-CH_3_(Mes)]; 18.8 [*o*-CH_3_(Mes)]; 10.4 ppm [CH_3_(Cp*)]. ^**77**^**Se NMR** (CDCl_3_, 300 K, data from HMBC): δ = 354 ppm.

### **15-B**: ^**1**^**H NMR**

(600 MHz, CDCl_3_, 300 K): δ 8.20 (s, 2H, H5,20); 7.81 (d, ^3^*J* = 4.7 Hz, 2H, H7,18); 7.42 (s, 2H, H12,13); 7.14 (s, 2H, *m*-Mes); 6.90 (d, ^3^*J* = 4.7 Hz, 2H, H8,17); 6.82 [s, 2H, *m*-(Mes)]; 5.46 (s, 2H, H2,3); 2.63 [s, 6H, *o*-CH_3_(Mes)]; 2.36 [s, 6H, *p*-CH_3_(Mes)]; 1.54 [s, 15H, CH_3_(Cp*)]; 1.14 ppm [s, 6H, *o*-CH_3_(Mes)]. ^**13**^**C NMR** (CDCl_3_, 300 K, data from HSQC and HMBC): δ 159.9 (C6,19/C9,16); 143.2 (C5,20); 142.6 (C6,19/C9,16); 141.3 (C11,14); 139.8 (C12,13); 136.2 (C7,18); 128.5 (*m*-Mes); 127.6 (H8,17); 92.6 (Cp*); 90.1 (C1,4); 88.8 (C21); 84.9 C(2,3); 21.0 [*o,p*-CH_3_(Mes)]; 19.2 [*o*-CH_3_(Mes)]; 10.6 ppm (CH_3_(Cp*)). ^**77**^**Se NMR** (CDCl_3_, 300 K, data from HMBC): δ = 346 ppm.

### Instrumentation

All ^1^H and ^13^C NMR spectra were recorded on high-field Bruker ADVANCE III spectrometers (^1^H frequencies 600 and 500 MHz), equipped with broadband inverse or conventional gradient probe heads. Spectra were referenced to the residual solvent signals (CDCl_3_, 7.24 ppm; CD_2_Cl_2_, 5.32 ppm). ^13^C NMR spectra were recorded with ^1^H broadband decoupling and referenced to solvent signals (^13^CDCl_3_, 77.0 ppm, ^13^CD_2_Cl_2_, 54.0 ppm). The ^77^Se–^1^H HMBC spectra were recorded on a JEOL JNM-ECZ500R 500 MHz spectrometer at 300 K and referenced to selenophene (δ = 605 ppm)^24^ used as an internal standard. High-resolution and accurate mass spectra were recorded using the electrospray ionization technique on a Bruker qTOF compact and Bruker micrOTOF-Q. Electronic spectra were recorded on a Varian Carry 60 UV–vis spectrophotometer.

### Computational Details

Geometry optimizations were carried out within a unconstrained *C*_1_ symmetry in vacuo, with starting coordinates derived from preoptimized models or crystal structures using Gaussian software.^27^ Harmonic frequencies were calculated using analytical second derivatives to verify the local minimum achievement, and no negative frequencies were observed. The calculations were performed at B3LYP/6–31G(d,p) level of theory.^28,29^ NICS values^30^ and NMR shifts were calculated using the GIAO method with TMS shieldings as a reference for NMR. For relative energy calculations, values with a zero-point correction were taken. 2D NICS(1)_*zz*_ map points were generated through the py.Aroma 4.0 tool^31^ and then processed in Origin. EDDB plots were obtained from population analysis (at the ωB97XD/def2sVP level of theory). The resulting data were analyzed through the EDDB program,^25,32^ and the output was visualized using Avogadro 1.2.

### X-ray Structure Solution and Refinement

X-ray quality crystals of **11** and **13·C**_**6**_**H**_**14**_ were prepared by slow diffusion of hexane to the solution of **11** and **13** in dichloromethane, respectively. Diffraction data for the crystals **11** and **13·C**_**6**_**H**_**14**_ were collected on a Rigaku κ-geometry XtaLAB Synergy R, DW system four-circle diffractometer (rotating anode X-ray source, ω scan method) with a hybrid HyPix-Arc 150 detector at 200(2) and 100(2) K, respectively. Mo Kα radiation for **11** and Cu Kα radiation for **13·C**_**6**_**H**_**14**_ was used. Data were corrected for Lorentz and polarization effects and absorption (by empirical or analytical methods; see Table S1 for details). Data collection, processing, and analysis were carried out with CrysAlis PRO.^33^ The structures were solved using a dual-space algorithm with the SHELXT program,^34^ and refined on *F*^2^ by a full-matrix least-squares technique using the SHELXL program,^35^ with anisotropic displacement parameters for the ordered (fully occupied) and selected positions of the disordered non-H atoms. Further details can be found in the Supporting Information.

## Supporting Information Available

Experimental details, materials and methods, X-ray data, spectroscopic data including NMR and UV/vis spectra, selected mass spectra, and DFT calculations (PDF)
