# Supplementary material for: Ruthenocenoporphyrinoids—π-Conjugation Transmitted across 1,3-Substituted Ruthenocene
Source: Inorg Chem. 2025 Apr 10;64(15):7552–60. doi: 10.1021/acs.inorgchem.5c00470 (PMC12015811; doi:10.1021/acs.inorgchem.5c00470)
Supplement: Supplementary file 1 — ic5c00470_si_001.pdf [file ic5c00470_si_001.pdf]

Supporting Information

Ruthenocenoporphyrinoids - π-Conjugation Transmitted Across 1,3-Substituted Ruthenocene

Anna Berlicka*, Aleksandra Walczak, Michał J. Białek, Katarzyna Ślepokura, Piotr J. Chmielewski, and Lechosław Latos-Grażyński*

Department of Chemistry
University of Wrocław
14 F. Joliot-Curie, 50-383 Wrocław, Poland
E-mail: anna.berlicka@uwr.edu.pl, lechoslaw.latos-grazynski@uwr.edu.pl

Table of Contents

1. General Information 3
2. Experimental Procedures 5
    2.1. Solvents and reagents 5
    2.2. Synthesis 5
3. NMR spectra 7
4. UV-Vis spectra 22
5. MS spectra 23
6. DFT calculations 26
    6.1. NICS values 26
    6.2. Optimized structures 26
    6.3. The EDDB plots and NICS 2D maps 28
    6.4. Correlation between calculated and experimental NMR values 29
    6.5. Cartesian coordinates 33
7. References 41
8. Author Contributions 41

1. General information

NMR Spectroscopy.
All ^{1}H and ^{13}C NMR spectra were recorded on high-field Bruker Advance III spectrometers (^{1}H frequency 600 and 500 MHz), equipped with broadband inverse or conventional gradient probe heads. Spectra were referenced to the residual solvent signals ($CDCl_3$, 7.24 ppm; CD_2Cl_2, 5.32 ppm). ^{13}C NMR spectra were recorded with ^{1}H broadband decoupling and referenced to solvent signals ($^{13}CDCl_3$, 77.0 ppm, $^{13}CD_2Cl_2$, 54.0 ppm). The ^{77}Se-^{1}H HMBC spectra were recorded on the JEOL JNM-ECZ500R 500 MHz spectrometer at 300 K and referenced to selenophene (δ = 605 ppm)[1,2] used as an internal standard.

Mass Spectrometry. High-resolution and accurate mass spectra were recorded using the electrospray ionization technique on Bruker qTOF compact and Bruker micrOTOF-Q.

UV-vis Spectroscopy. Electronic spectra were recorded on a Varian Carry 60 UV-Vis spectrophotometer.

Theoretical calculations. Geometry optimizations were carried out within unconstrained C_1 symmetry in vacuo, with starting coordinates derived from preoptimized models or crystal structures using Gaussian software.[3] Harmonic frequencies were calculated using analytical second derivatives to verify local minimum achievement, and no negative frequencies were observed. The calculations were performed at B3LYP/6-31G(d,p) level of theory.[4,5] NICS values[6] and NMR shifts were calculated using the GIAO method with TMS shieldings as a reference for NMR. For relative energy calculations, values with zero-point correction were taken. 2D $NICS(1)_{zz}$ map points were generated through the py.Aroma 4.0 tool[7] and then processed in Origin. EDDB plots were obtained from population analysis (at the ωB97XD/def2sVP level of theory). The resulting data were analyzed through the EDDB program,[8-10] and the output was visualized using Avogadro 1.2.

X-ray data. X-ray quality crystals of **11** and **13·C_6H_{14}** were prepared by slow diffusion of hexane to the solution of **11** and **13** in dichloromethane, respectively. Diffraction data for the crystals **11** and **13·C_6H_{14}** were collected on a Rigaku κ-geometry XtaLAB Synergy R, DW system four-circle diffractometer (rotating anode X-ray source, ω scan method) with hybrid HyPix-Arc 150 detector, at 200(2) and 100(2) K, respectively. Mo Kα radiation for **11** and Cu Kα for **13·C_6H_{14}** was used. Data were corrected for Lorentz and polarization effects and absorption (by the empirical or analytical methods; see Table S1 for details). Data collection, processing, and analysis were carried out with *CrysAlis PRO*.[11] The structures were solved using dual-space algorithm with the *SHELXT* program,[12] and refined on F^2 by a full-matrix least-squares technique using the *SHELXL* program,[13] with anisotropic displacement parameters for the ordered (fully occupied) and selected positions of the disordered non-H atoms.

Crystal of **11** was measured at 200 K due to additional weak reflections present in the diffraction pattern below this temperature, which most likely originates from a phase transition that the crystals undergo between 150-200 K (the temperature range determined by analyzing the diffraction pattern at 100, 120, 150, 200, 220 and 240 K). Therefore, to collect diffraction data for **11**, the crystal was mounted on the diffractometer at 240 and cooled to 200 K, and its high-temperature phase is presented here. The crystal is non-centrosymmetric (triclinic space group *P*1) and is disordered in the region of one of the mesityl (Mes) groups and the pentamethylcyclopentadienyl (Cp*) ring. The mesityl group was refined in two positions with site-occupation-factors (SOFs) of 0.839(16) and 0.161(16). The Cp* ring was refined in three positions with SOFs = 0.617(11), 0.211(12) and 0.174(6). Positions with SOF > 0.5 were refined with anisotropic displacement parameters. The remaining ones were refined isotropically. Some geometrical restraints (SAME instructions in *SHELXL*), constraints on the coordinates and displacement parameters (EXYZ and EADP instructions), restraints on the anisotropic displacement parameters (SIMU, ISOR), and restraints on the sum of SOFs (SUMP) were applied to get reasonable models of the disordered fragments.

Crystal of **13·C_6H_{14}** is centrosymmetric (monoclinic space group *C*2/*m*). The asymmetric unit contains half a **13** molecule and half an *n*-hexane molecule (both lying on a mirror plane). The solvent molecule was found to be disordered about the mirror plane and was refined in two positions with SOFs = 0.346(9) (refined anisotropically) and 0.154(9) (refined isotropically). Some geometrical restraints (DFIX, SADI, SAME instructions) and restraints on the anisotropic displacement parameters (SIMU) were applied to the disordered hexane molecule.

The hydrogen atoms in **11** and **13·C_6H_{14}** were included using geometrical considerations and refined using a riding model, with C–H = 0.95-0.99 Å, and with $U_{iso}(H) = 1.2U_{eg}(C)$ for CH and CH_2 or $1.5U_{eg}(C)$ for CH_3.
The details of structure refinements are given in Table S1. The crystallographic information files (CIF) are deposited at the Cambridge Crystallographic Data Centre (CCDC No. 2400821 for **11** and CCDC No. 2400822 for **13·C_6H_{14}**) and provided as Supporting Information.

Table S1. Crystal data for **11** and **13·C_6H_{14}**.

	**11**	**13·C_6H_{14}**
**CCDC No.**	2400821	2400822
**Chemical formula**	$C_{49}H_{48}N_2RuSe$	$C_{45}H_{48}N_2PdSe$
**M_r**	844.92	802.21
**Crystal system, space group**	Triclinic, *P*1	Monoclinic, *C*2/*m*
**Temperature (K)**	200(2)	100(2)
***a*, *b*, *c* (Å)**	7.9320(16), 11.639(2), 12.346(2)	15.540(3), 17.140(3), 14.463(3)
**α, β, γ (°)**	63.86(2), 82.79(2), 77.99(2)	90, 109.11(2), 90
**V (Å^3)**	1000.1(4)	3640.0(13)
**Z**	1	4
**Density calculated (g cm^{-3})**	1.403	1.464
**Radiation type, λ (Å)**	Mo *K*α, 0.71073	Cu *K*α, 1.5418
**θ range (°)**	1.8–30.0	3.2–67.5
**$(\sin \theta/\lambda)_{max}$ (Å^{-1})**	0.703	0.599
**Index ranges**	$-11 \leq h \leq 10$,<br>$-16 \leq k \leq 16$,<br>$-17 \leq l \leq 17$	$-18 \leq h \leq 18$,<br>$-20 \leq k \leq 20$,<br>$-17 \leq l \leq 17$
**F(000)**	434	1648
**Crystal size (mm)**	0.24 × 0.09 × 0.03	0.11 × 0.06 × 0.02
**Diffractometer**	Rigaku, XtaLAB Synergy R, DW system with HyPix-Arc 150 detector	Rigaku, XtaLAB Synergy R, DW system with HyPix-Arc 150 detector
**μ (mm^{-1})**	1.34	5.51
**Absorption correction**	Empirical (multi-scan)	Analytical
**T_{min}, T_{max}**	0.823, 1.000	0.626, 0.906
**No. of measured, independent and observed [$I > 2\sigma(I)$] reflections**	29789, 11130, 9962	15552, 3402, 3137
**R_{int}**	0.028	0.028
**$R[F^2 > 2\sigma(F^2)]$, $wR(F^2)$, S**	0.028, 0.059, 1.04	0.034, 0.096, 1.10
**No. of reflections**	11130	3402
**No. of parameters**	622	284
**No. of restraints**	139	46
**H-atom treatment**	H-atom parameters constrained	H-atom parameters constrained
**$\Delta\rho_{max}$, $\Delta\rho_{min}$ (e Å^{-3})**	0.28, −0.37	1.57, −1.17
**Absolute structure parameter**	0.002(3)	–

Computer programs: *CrysAlis PRO* 1.171.41.80a (Rigaku OD, 2020), *SHELXT*-2014 (Sheldrick, 2015), *SHELXL2014*/7 (Sheldrick, 2015).

2. Experimental procedures

2.1. Solvents and reagents

If not indicated differently, all solvents (chloroform, dichloromethane, ethyl acetate, n-hexane, methanol) were used without purification. Dry dichloromethane was used as received (Sure/Seal™ System, stored in the glovebox). $CDCl_3$ was prepared directly before use by running through a basic alumina column. Reagents not listed here were used as received. 21-Carba-23-selenaporphyrinoids **1** and **2** were synthesized as described in the literature.[14] The ruthenium π complexes **11** and **15** were synthesized according to the established procedures, with modifications to the reaction conditions (see below). [15,16]

2.2. Synthesis

Ruthenium(II) pentamethylcyclopentadienyl π-complex of 21-carba-23-selenaporphyrin 11

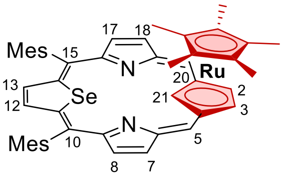

11

9.4 mg (0.015 mmol) of **9** was placed in a 5-mL vial and introduced into the glovebox. The 4 mg (0.008 mmol) of $[RuCp^*(CH_3CN)_3](PF_6)$ and 2 mL of dry CH_2Cl_2 were added. The mixture was stirred for 10 min. and removed from the glove box. The solvent was evaporated with nitrogen and the residue was separated on a silica gel column (mesh 70–230) with CH_2Cl_2. The product **11** was identified in the first fraction. After subsequent chromatography (silica gel, mesh 70-230; CH_2Cl_2) compound **11** was eluted in the first fraction with 62% yield (4.2 mg). An increase in the amount of $[RuCp^*(CH_3CN)_3](PF_6)$ (1–2 equiv) has been observed to result in a reduction in the efficiency of the synthesis of compound **11**.

UV-vis (CH_2Cl_2): λ_{max} ($\log\varepsilon$) = 299 (4.2), 385 (4.3), 449 (4.5), 692 nm (3.3).

^{1}H NMR (600 MHz, CD_2Cl_2, 300 K): δ = 7.96 (s, 2H, H5,20); 7.78 (d, ^3J = 4.3 Hz, 2H, H7,18); 7.61 (s, 2H, H12,13); 7.12 (s, 2H, *m*-Mes); 7.05 (s, 2H, *m*-Mes); 6.97 (d, ^3J = 4.3 Hz, 2H, H8,17); 5.35 (d, ^4J = 1.2 Hz, 2H, H2,3); 3.47 (s, 1H, H21); 2.44 (s, 6H, *p*-CH_3(Mes)); 2.18 (s, 6H, *o*-CH_3(Mes)); 1.82 (s, 6H, *o*-CH_3(Mes)); 0.98 ppm (s, 15H, CH_3(Cp*)).

^{13}C NMR (150.9 MHz, CD_2Cl_2, 300 K): δ = 166.5; 153.7; 151.8; 138.3; 138.2; 137.7; 137.0 (C7,18); 136.52 (C12,13); 136.47; 134.9 (C5,20); 130.7; 128.5 (C(*m*-Mes), C(*m'*-Mes)); 127.1 (C8,17); 89.1 (C1,4); 86.5; 82.4 (C2,3); 77.3 (C21); 21.5; 21.3; 20.7; 10.1 ppm (CH_3(Cp*)).

^{77}Se NMR ($CDCl_3$, 300 K, data from HMBC): δ = 598 ppm.

HR-MS (ESI): *m/z* calcd for $C_{49}H_{49}N_2RuSe^+$ $[M+H]^+$: 847.2119; found: 847.2105.

11-H$^+$ and **11-H$_2^{2+}$** were obtained by titrating **11** dissolved in CD_2Cl_2 with $HBF_4{:}Et_2O$ solution (^{1}H NMR) or by titrating **11** dissolved in CH_2Cl_2 with TFA solution (UV/Vis).

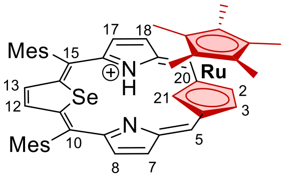

11-H$^+$

11-H$^+$: UV-vis (CH_2Cl_2): λ_{max} ($\log\varepsilon$) = 306 (4.1), 395 (4.3), 473 (4.3), 768 nm (3.4).

^{1}H NMR (600 MHz, CD_2Cl_2, 190 K): δ = 8.76 (s, 1H, H5/20); 8.35 (s, 1H, H5/20); 8.27 (s, 1H, H7/H18); 8.02 (s, 2H, H7/H18, H12/H13); 7.92 (s, 2H, H12/H13); 7.25 (s, 1H, H8/H17); 7.22 (s, 1H, H8/H17); 7.13 (s, 2H, *m*-Mes); 7.08 (s, 2H, *m*-Mes); 6.13 (s, 1H, H2/H3); 5.56 (s, 1H, H2/H3); 4.25 (s, 2H, NH); 2.41 (s, 6H, *p*-CH_3(Mes)); 2.04 (s, 6H, *o*-CH_3(Mes)); 1.78 (s, 3H, *o*-CH_3(Mes)); 1.72 ppm (s, 3H, *o*-CH_3(Mes)).

NH signal was identified in the ^{1}H NMR spectrum after adding D_2O.

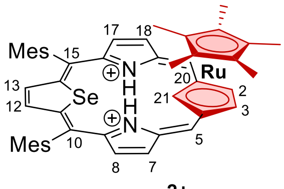

11-H$_2^{2+}$

11-H$_2^{2+}$: UV-vis (CH_2Cl_2): λ_{max} ($\log\varepsilon$) = 320 (4.2), 397 (4.2), 494 (4.4), 715 (3.5), 984 nm (3.2).

^{1}H NMR (600 MHz, CD_2Cl_2, 300 K): δ = 8.84 (s, 2H, H5,20); 8.37 (d, ^3J = 4.6 Hz, 2H, H7,18); 8.20 (s, 2H, H12,13); 7.49 (d, ^3J = 4.6 Hz, 2H, H8,17); 7.23 (s, 2H, *m*-Mes); 7.14 (s, 2H, *m*-Mes); 5.83 (s, 2H, NH); 5.79 (s, 2H, H2,3); 2.47 (s, 6H, *p*-CH_3(Mes)); 2.26 (s, 6H, *o*-CH_3(Mes)); 1.89 (s, 6H, *o*-CH_3(Mes)); 1.46 ppm(s, 1H, H21).

Palladium(II) 2-oxo-21-carba-23-selenachlorin 12 and palladium(II) 21-carba-23-selenachlorin 13

Macrocycle **10** (12.8 mg, 0.021 mmol) and 5 mL of DMF were placed in a 2-neck 25-mL flask. Nitrogen was bubbled through the solution for 10 min, then K_2CO_3 (a few mg) and $PdCl_2$ (37.2 mg, 0.21 mmol) were added, and the mixture was stirred in reflux under nitrogen for 10 min. The residue was separated on a silica gel column (mesh 70–230). Complex **13** was removed in the first fraction with CH_2Cl_2 as eluant, while complex **12** was eluted with 1% MeOH in CH_2Cl_2 in the second fraction. After subsequent chromatography of both complexes **12** and **13** (basic alumina, Brockmann III grade; 50% hexane in CH_2Cl_2), the compounds were obtained with 5% (0.8 mg) and 39% yield (5.8 mg), respectively. The selective formation of complex **13** was observed with a yield of 58% (14.1 mg) when the reaction of **10** (21 mg, 0.034 mmol) with 10 equiv of $PdCl_2$ in the presence of K_2CO_3 proceeded in a mixture of $CHCl_3/CH_3CN$ (16 ml, 1:1, *V*:*V*) at reflux (reaction time 0.5 h).

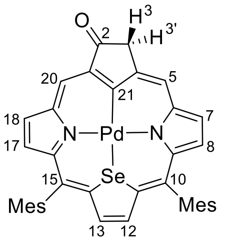

12

12: UV-vis (CH_2Cl_2): λ_{max} (logε) = 348 (4.3), 489 (4.7), 714 nm (4.0).
^{1}H NMR (600 MHz, $CDCl_3$, 300 K): δ = 10.10 (s, 1H, H5); 9.75 (s, 1H, H20); 9.06 (d, ^3J = 4.6 Hz, 1H, H7); 8.98 (d, ^3J = 4.6 Hz, 1H, H18); 8.88 (d, ^3J = 5.2 Hz, 1H, H12); 8.81 (d, ^3J = 5.2 Hz, 1H, H13); 8.60 (d, ^3J = 4.6 Hz, 1H, H17); 8.55 (d, ^3J = 5.2 Hz, 1H, H8); 7.45 (s, 2H, *m*-Mes); 6.95 (s, 1H, *m*-Mes); 6.94 (s, 1H, *m*-Mes); 5.40 (d, ^2J = 20.0 Hz, 1H, H3'); 5.20 (d, ^2J = 20.0 Hz,1H, H3); 2.94 (s, 3H, *o*-CH_3Mes)); 2.93 (s, 3H, *o*-CH_3(Mes)); 2.55 (s, 3H, *p*-CH_3(Mes)); 2.54 (s, 3H, *p*-CH_3(Mes)); 0.39 (s, 3H, *o*-CH_3(Mes)); 0.38 ppm (s, 3H, *o*-CH_3(Mes)).
^{13}C NMR ($CDCl_3$, 300 K, data from HSQC and HMBC): δ = 205.2 (C2); 150.2 (C14); 148.0 (C11); 138.0 (C13); 136.6 (C12); 134,4 (C18); 132.1 (C7); 130.9 (C17); 130.0 (C8); 128.2 (*m*-Mes); 119.4 (C5); 114.5 (C20); 47.5 (C3); 22.2 (*o*-CH_3(Mes)); 21.3 (*p*-CH_3(Mes)); 19.0 (*o*-CH_3(Mes)) ppm.
HR-MS (ESI): *m/z* calcd for $C_{39}H_{32}N_2OPdSe^+$ $[M]^+$: 730.0728; found: 730.0756.

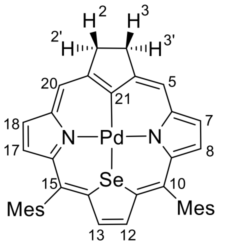

13

13: UV-vis (CH_2Cl_2): λ_{max} (logε) = 335 (4.4), 386 (4.2), 487 (4.8), 718 nm (4.1).
^{1}H NMR (600 MHz, $CDCl_3$, 300 K): δ = 9.62 (s, 2H, H5,20); 8.93 (d, ^3J = 4.6 Hz, 2H, H7,18); 8.75 (s, 2H, H12,13); 8.62 (d, ^3J = 4.6 Hz, 2H, H8,17); 7.43 (s, 2H, *m*-Mes); 6.96 (s, 2H, *m*-Mes); 5.02 (dd, ^2J = 16.2 Hz, ^3J = 3.5 Hz, 2H, H2,3); 4.80 (dd, ^2J = 16.2 Hz, ^3J = 3.5 Hz, 2H, H2',3'); 2.89 (s, 6H, *o*-CH_3(Mes)); 2.56 (s, 6H, *p*-CH_3(Mes)); 0.46 ppm (s, 6H, *o*-CH_3(Mes)).
^{13}C NMR (150.9 MHz, $CDCl_3$, 300 K): δ = 150.5 (C6,19); 148.3 (C11,14); 147.5 (C1,4); 144.6 (C9,16); 142.7 (C21); 139.1; 139.0; 138.1; 137.8; 135.0 (C12,13); 131.6 (C7,18); 130.0 (C8,17); 128.2 (*m*-Mes); 128.0 (*m*-Mes); 115.3 (C5,20); 35.3 (C2,3); 22.3 (*o*-CH_3(Mes)); 21.4 (*p*-CH_3(Mes)); 19.1 ppm (*o*-CH_3(Mes)).
HR-MS (ESI): *m/z* calcd for $C_{39}H_{34}N_2PdSe^+$ $[M]^+$: 716.0935; found: 716.0981.

Palladium(II) 21-carba-23-selenaporphyrin 14

Complex **14** was prepared on an NMR scale. **13** (3.8 mg, 0.0053 mmol) was dissolved in $CDCl_3$ and transferred to an NMR tube. The solution of **13** was titrated with a saturated solution of DDQ in $CDCl_3$. The product was purified by column chromatography on a silica gel column (mesh 70-230) with CH_2Cl_2 as eluant. Compound **14** was obtained with a 55% (2.1 mg) yield.

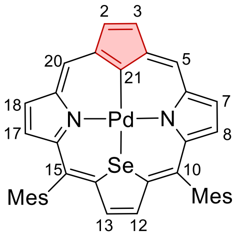

14

UV-vis (CH_2Cl_2): λ_{max} (logε) = 354 (4.6), 410 (4.5), 455 (4.5), 497 (4.3), 527 (4.3), 570 (4.3), 692 (3.9), 788 nm (3.7).
^{1}H NMR (600 MHz, $CDCl_3$, 300 K): δ = 9.25 (s, 2H, H5,20); 8.50 (d, ^3J = 4.5 Hz, 2H, H7,18); 8.49 (s, 2H, H12,13); 7.97 (d, ^3J = 4.5 Hz, 2H, H8,17); 7.56 (s, 2H, H2,3); 7.34 (s, 2H, *m*-Mes); 6.85 (s, 2H, *m*-Mes); 2.90 (s, 6H, *o*-Me(Mes)); 2.48 (s, 6H, *p*-CH_3(Mes)); 0.44 ppm (s, 6H, *o*-CH_3(Mes)).
^{13}C NMR (150.9 MHz, $CDCl_3$, 300 K): δ = 153.1 (C6,19/C9,16); 148.2 (C11,14); 142.9 (C6,19/C9,16); 138.9; 138.7 (C12,13); 138.3; 138.0; 137.4 (C1,4); 136.7; 135.9 (C21); 135.1; 133.3 (C7,18); 132.9 (C2,3); 128.3 (*m*-Mes); 128.2 (*m*-Mes); 127.8 (C8,17); 126.9 (C5,20); 21.9 (*o*-CH_3(Mes)); 21.3 (*p*-CH_3(Mes)); 19.0 ppm (*o*-CH_3(Mes)).
HR-MS (ESI): *m/z* calcd for $C_{39}H_{32}N_2PdSeCl^+$ $[M+Cl]^+$: 749.0463; found: 749.0490.

Ruthenium(II) pentamethylcyclopentadienyl π-complex of palladium(II) 21-carba-23-selenaporphyrin 15

3.9 mg (0.0055 mmol) of **14** was transferred to a 5-mL vial and placed in the glove box. The 3.2 mg (0.0069 mmol) of $[RuCp^*(CH_3CN)_3](PF_6)$ and 1.5 mL of dry CH_2Cl_2 were added. The mixture was stirred for 10 min. and removed from the glove box. The residue was separated on a silica gel column (mesh 70–230) without evaporating the solvent. Product **15** was eluted in the second fraction with 5% ethyl acetate in CH_2Cl_2. **15** was obtained with a 96% (5 mg) yield.
Finally, two **15** (**A** and **B**) stereoisomers resolutions were performed using HPLC on an achiral stationary phase analytical column (5u, silica gel, 25x0.46 cm) with 5% methanol in CH_2Cl_2 as the eluant. The isomer **15-B** was identified in the first fraction, while the isomer **15-A** was identified in the third fraction. The principal second fraction contained a mixture of two isomers. Complexes **15** are stable in anaerobic conditions.

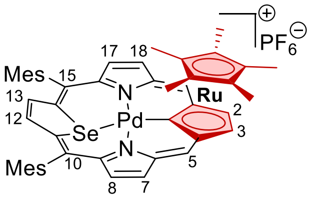

15-A

UV-vis for a mixture of two isomers of **15** (CH_2Cl_2): λ_{max} (logε) = 302 (4.2), 404 (4.2), 497 nm (4.3).
15-A: ^{1}H NMR (600 MHz, $CDCl_3$, 300 K): δ = 8.60 (s, 2H, H5,20); 7.93 (d, ^3J = 4.8 Hz, 2H, H7,18); 7.62 (s, 2H, H12,13); 7.20 (s, 2H, *m*-Mes); 7.05 (d, ^3J = 4.8 Hz, 2H, H8,17); 6.84 (s, 2H, *m*-(Mes)); 5.60 (s, 2H, H2,3); 2.64 (s, 6H, *o*-CH_3(Mes)); 2.39 (s, 6H, *p*-CH_3(Mes)); 1.17 (s, 15H, CH_3(Cp*)); 1.00 ppm (s, 6H, *o*-CH_3(Mes)).
^{13}C NMR ($CDCl_3$, 300 K, data from HSQC and HMBC): δ = 160.4 (C6,19/C9,16); 148.3 (C5,20); 144.8 (C6,19/C9,16); 143.7 (C11,14); 140.7 (C12,13); 137.3 (C7,18); 129.0 (H8,17); 128.8 (*m*-Mes); 128.6 (*m*-Mes); 103.8 (C21); 90.5 (Cp*); 89.0 (C1,4); 84.4 C(2,3); 21.0 (*o*,*p*-CH_3(Mes)); 18.8 (*o*-CH_3(Mes)); 10.4 ppm (CH_3(Cp*)).
^{77}Se NMR ($CDCl_3$, 300 K, data from HMBC): δ = 354 ppm.

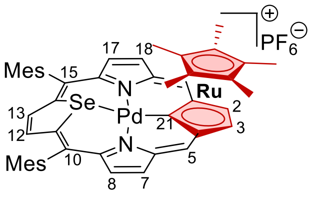

15-B

15-B: **^{1}H NMR** (600 MHz, $CDCl_3$, 300 K): δ = 8.20 (s, 2H, H5,20); 7.81 (d, ^3J = 4.7 Hz, 2H, H7,18); 7.42 (s, 2H, H12,13); 7.14 (s, 2H, *m*-Mes); 6.90 (d, ^3J = 4.7 Hz, 2H, H8,17); 6.82 (s, 2H, *m*-(Mes)); 5.46 (s, 2H, H2,3); 2.63 (s, 6H, *o*-CH_3(Mes)); 2.36 (s, 6H, *p*-CH_3(Mes)); 1.54 (s, 15H, CH_3(Cp*)); 1.14 ppm (s, 6H, *o*-CH_3(Mes)).

^{13}C NMR ($CDCl_3$, 300 K, data from HSQC and HMBC): δ = 159.9 (C6,19/C9,16); 143.2 (C5,20); 142.6 (C6,19/C9,16); 141.3 (C11,14); 139.8 (C12,13); 136.2 (C7,18); 128.5 (*m*-Mes); 127.6 (H8,17); 92.6 (Cp*); 90.1 (C1,4); 88.8 (C21); 84.9 C(2,3); 21.0 (*o,p*-CH_3(Mes)); 19.2 (*o*-CH_3(Mes)); 10.6 ppm (CH_3(Cp*)).

^{77}Se NMR ($CDCl_3$, 300 K, data from HMBC): δ = 346 ppm.

HR-MS (ESI): *m/z* calcd for $C_{49}H_{47}$ $N_2PdRuSe^+$ [M-PF_6]$^+$: 951.1010; found: 951.1053.

Alternatively, complex **15** (one stereoisomer, probably **15-B** with chloride as an anion) was obtained using **11** as a ligand. 2.6 mg of **11** (0.0031 mmol) and 4 mL of $CHCl_3$/MeCN (1:1) were placed in a 2-neck 25-mL flask. Nitrogen was bubbled through the solution for 5 min, then K_2CO_3 (a few mg) and $PdCl_2$ (6.2 mg, 0.035 mmol) were added, and the mixture was stirred in reflux under nitrogen for 10 min. The residue was separated on a silica gel column (mesh 70-230). Complex **15** was removed in the last fraction with 5% MeOH in CH_2Cl_2 as eluant.

^{1}H NMR (600 MHz, $CDCl_3$, 300 K): δ = 8.40 (s, 2H, H5,20); 7.18 (d, ^3J = 4.5 Hz, 2H, H7,18); 7.37 (s, 2H, H12,13); 7.12 (s, 2H, *m*-Mes); 6.85 (d, ^3J = 4.5 Hz, 2H, H8,17); 6.81 (s, 2H, *m*-(Mes)); 5.71 (s, 2H, H2,3); 2.61 (s, 6H, *o*-CH_3(Mes)); 2.35 (s, 6H, *p*-CH_3(Mes)); 1.61 (s, 15H, CH_3(Cp*)); 1.16 ppm (s, 6H, *o*-CH_3(Mes)).

3. NMR spectra

The NMR data are available at ZENODO at doi.org/10.5281/zenodo.14099129.

NMR spectra of 11

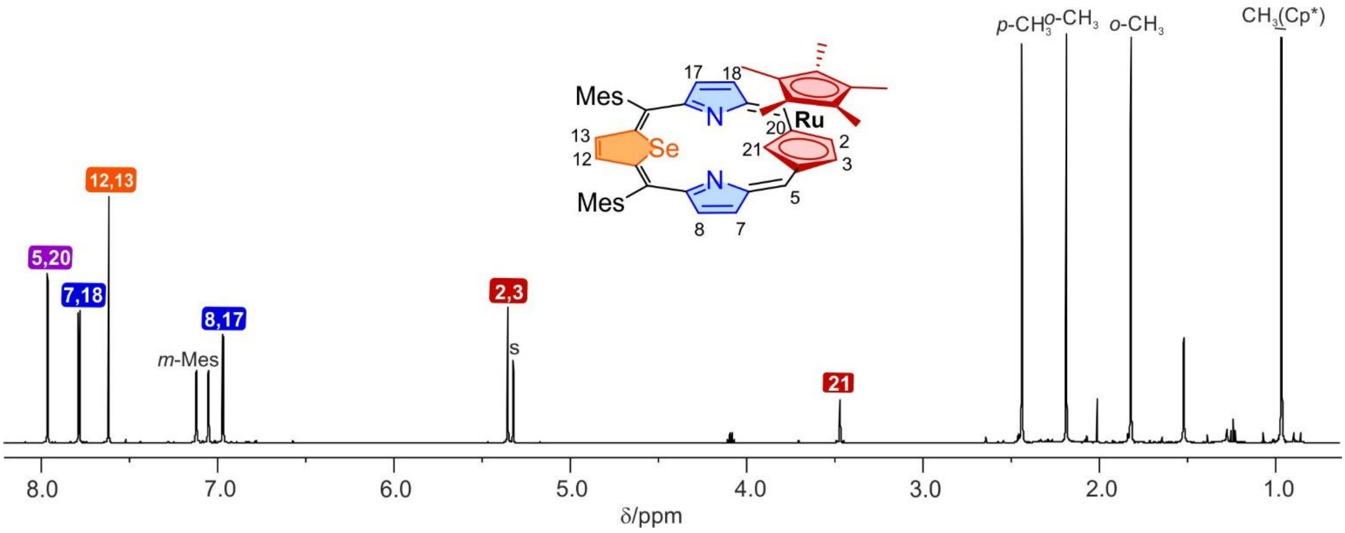

Figure S1. ^{1}H NMR spectrum of **11** (600 MHz, CD_2Cl_2, 300 K).

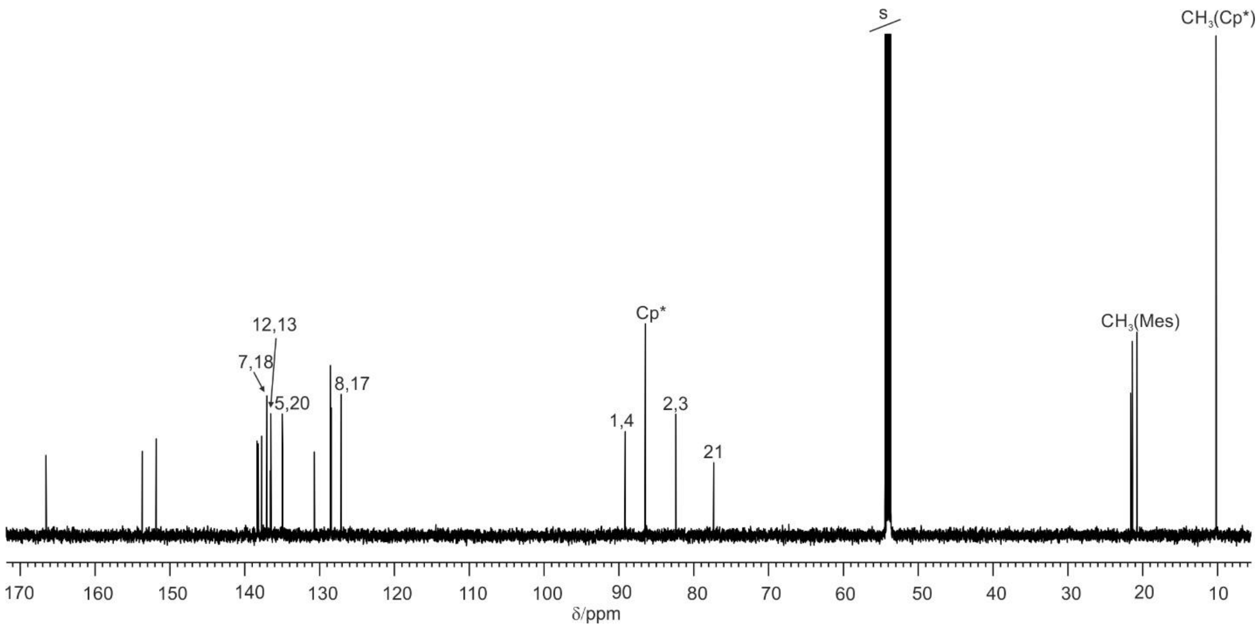

Figure S2. ^{13}C NMR spectrum of **11** (150.9 MHz, CD_2Cl_2, 300 K).

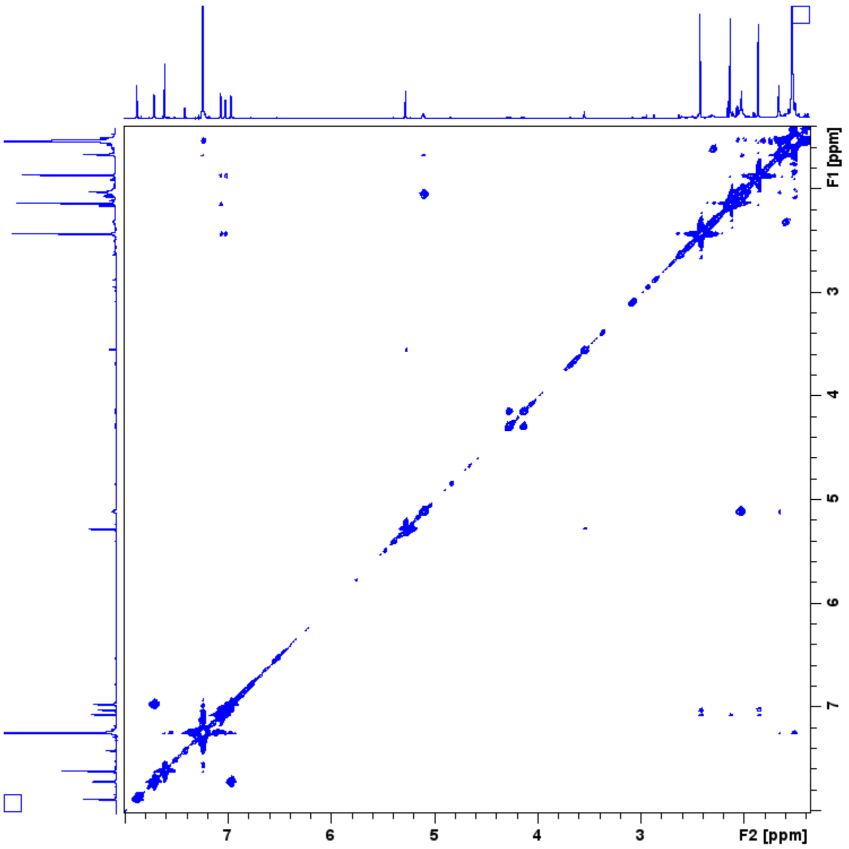

Figure S3. The ^{1}H-^{1}H COSY spectrum of **11** (600 MHz, $CDCl_3$, 300 K).

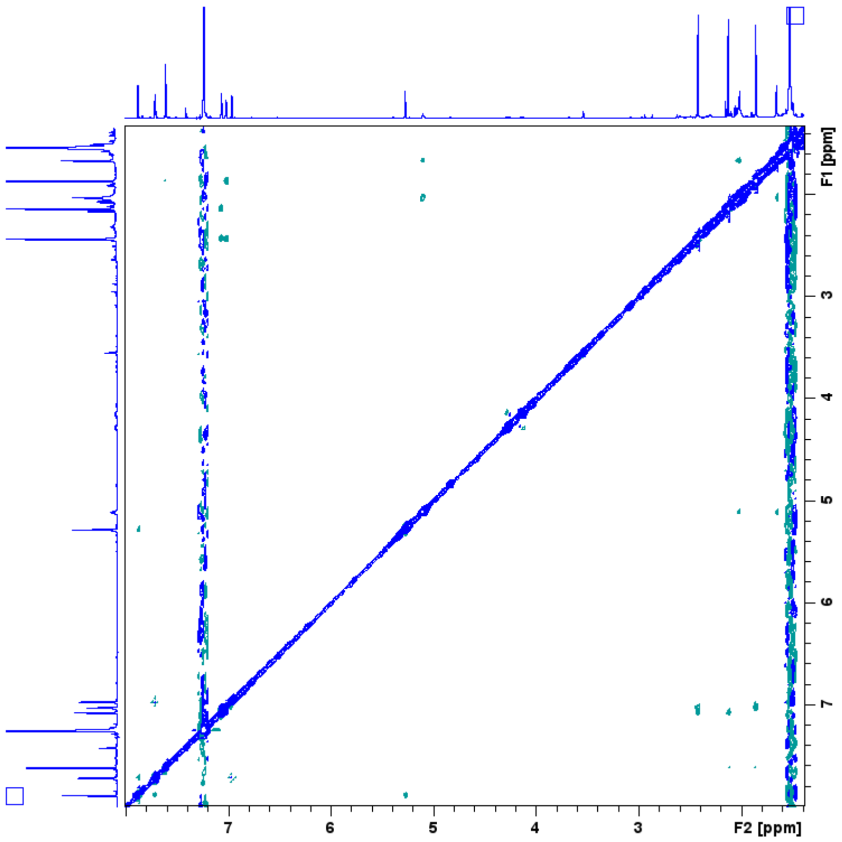

Figure S4. The ^{1}H-^{1}H NOESY spectrum of **11** (600 MHz, $CDCl_3$, 300 K).

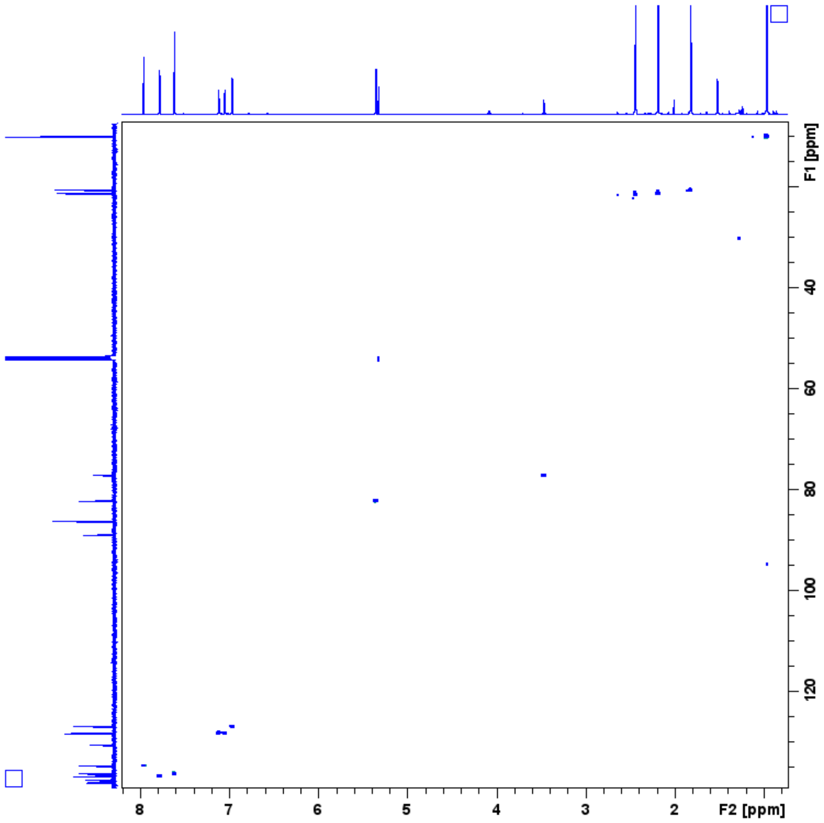

Figure S5. The ^{1}H-^{13}C HSQC spectrum of **11** (600 MHz, CD_2Cl_2, 300 K).

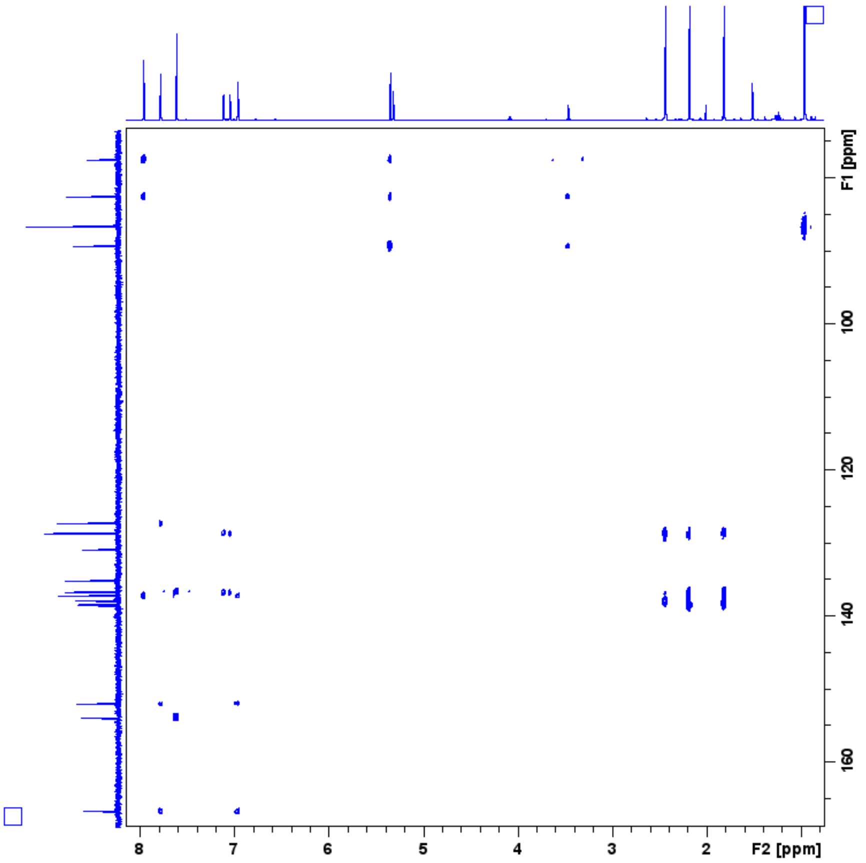

Figure S6. The ^{1}H-^{13}C HMBC spectrum of **11** (600 MHz, CD_2Cl_2, 300 K).

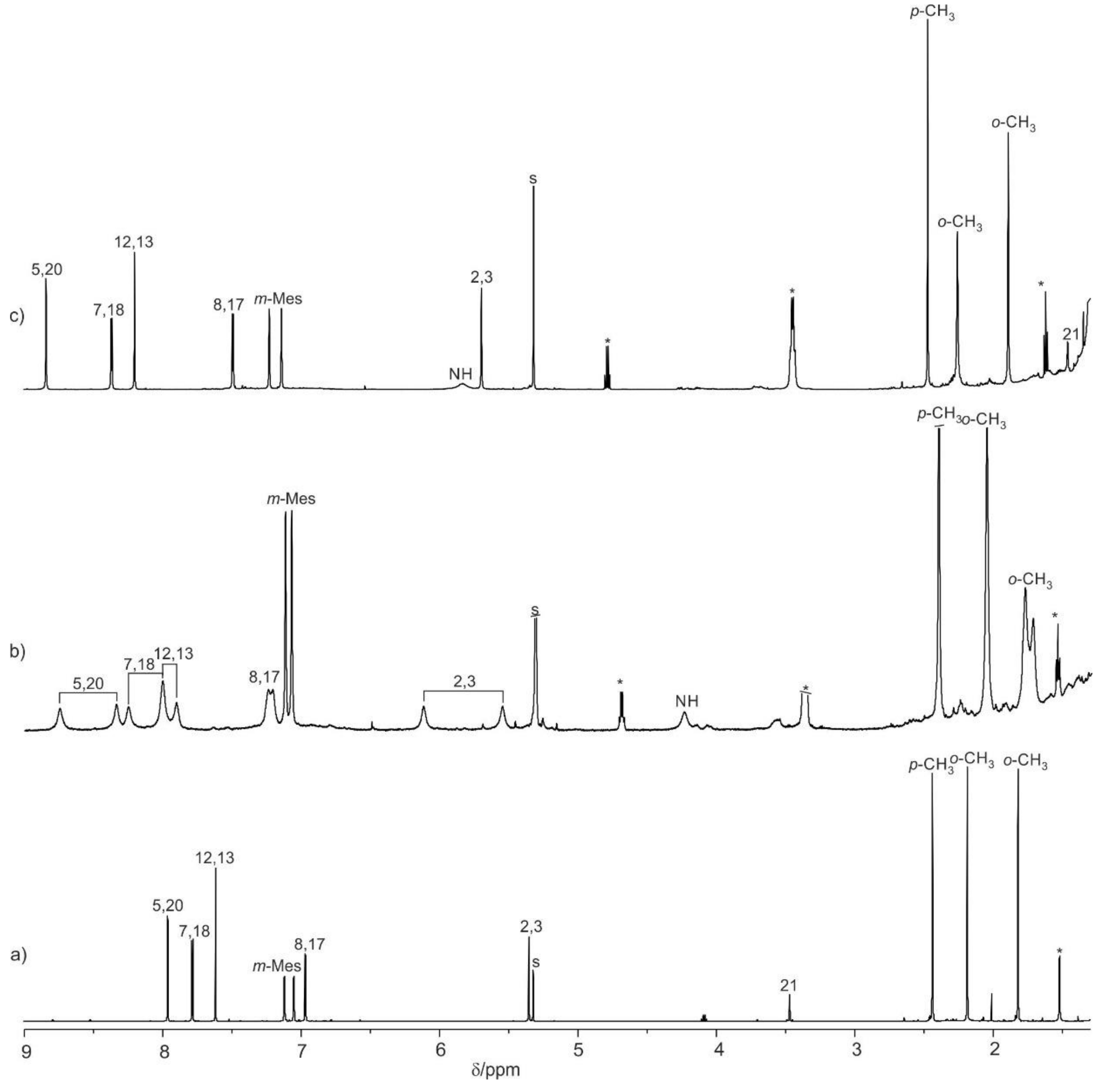

Figure S7. ^{1}H NMR spectra of a) **11** (300 K); b) **11-H⁺** (190 K), and c) **11-H$_2^{2+}$** (300 K) (600 MHz, CD_2Cl_2).

Table S2 Comparison of ^{1}H NMR chemical shifts for **9**, **11, 11-H$^+$**, and **11-H$_2^{2+}$**.

Position	^{1}**H NMR,** δ(ppm)			
	**9-I** 300 K, $CDCl_3$	**11** 300 K, CD_2Cl_2	**11-H$^+$** 190 K, CD_2Cl_2	**11-H$_2^{2+}$** 300 K, CD_2Cl_2
**5,20**	10.43	7.96	8.76, 8.35	8.84
**2,3**	10.14	5.35	6.13, 5.56	5.70
**12,13**	10.02	7.61	8.02, 7.92	8.20
**7,18**	8.95	7.78	8.27, 8.02	8.37
**8,17**	8.76	6.97	7.25, 7.22	7.49
**21**	−6.65	3.47	not identified	1.47
***m*-Mes**	7.27	7.12, 7.05	7.13, 7.08	7.23, 7.14
***p*-CH$_3$**	2.60	2.44	2.41	2.47
***o*-CH$_3$**	1.91	2.18, 1.82	2.04, 1.78, 1, 72	2.26, 1.89
**NH**	-	-	4.25	5.83

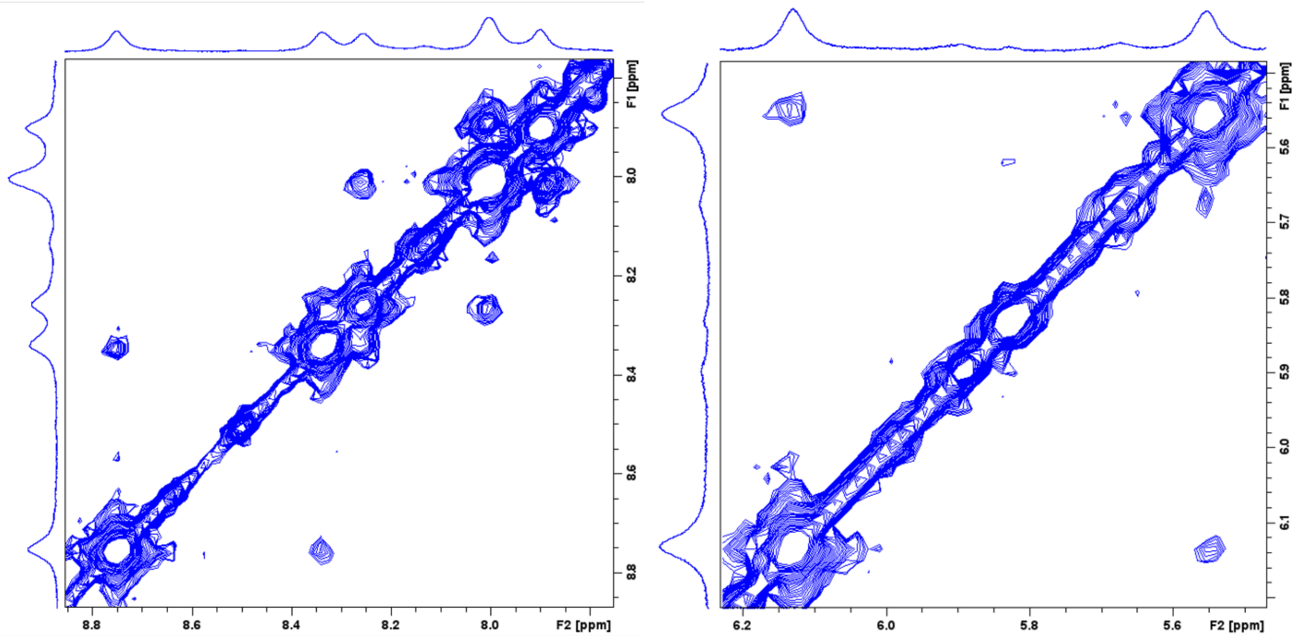

Figure S8. Fragments of the ^1H-^1H COSY spectrum of **11-H$^+$** (600 MHz, CD_2Cl_2, 190 K).

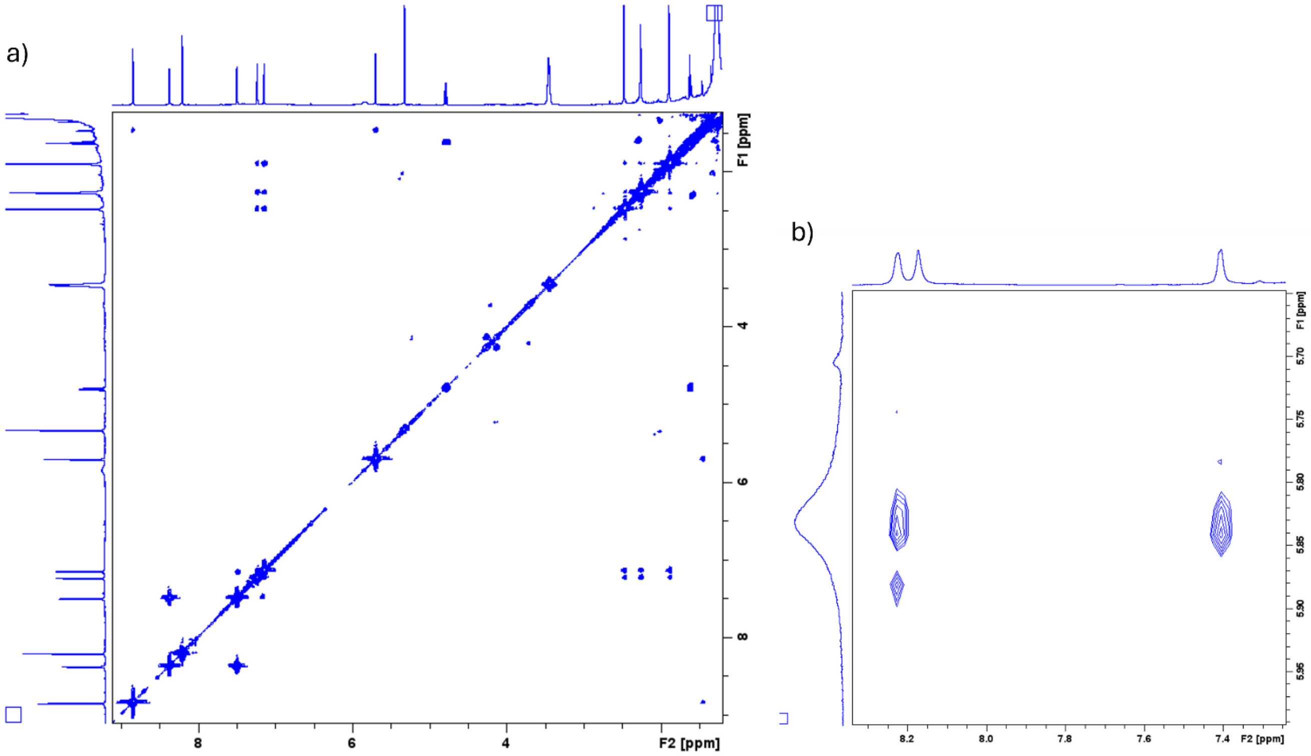

Figure S9. The ^1H-^1H COSY spectra of **11-H$_2^{2+}$**: a) 600 MHz, CD_2Cl_2, 300 K; b) 600 MHz, CD_2Cl_2, 190 K.

NMR spectra of 12

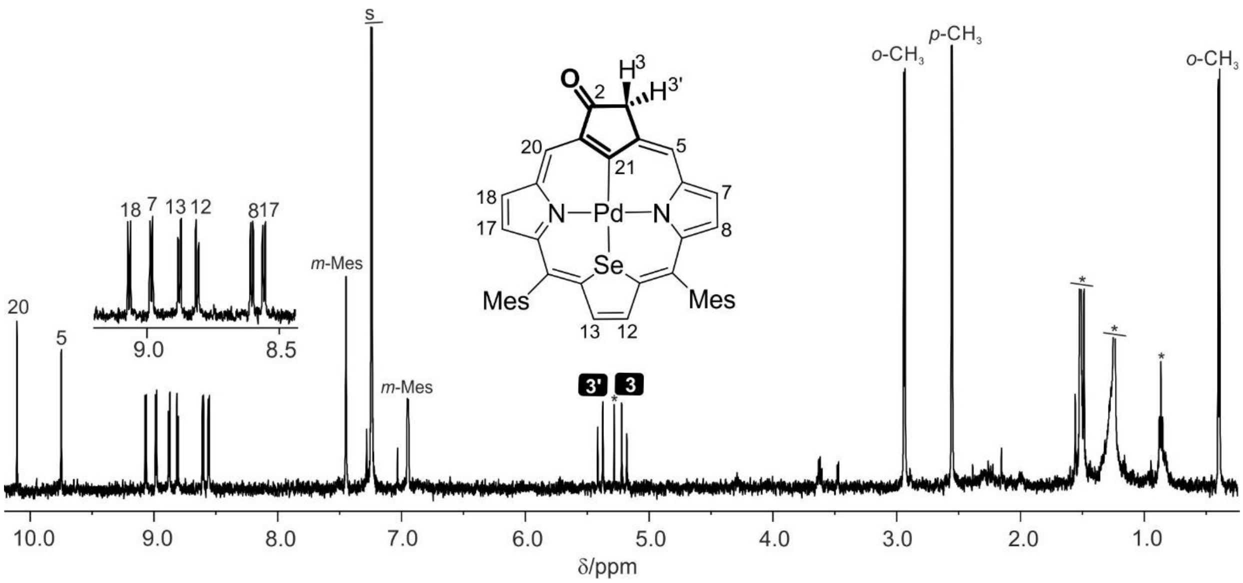

Figure S10. ^{1}H NMR spectrum of **12** (600 MHz, $CDCl_3$, 300 K).

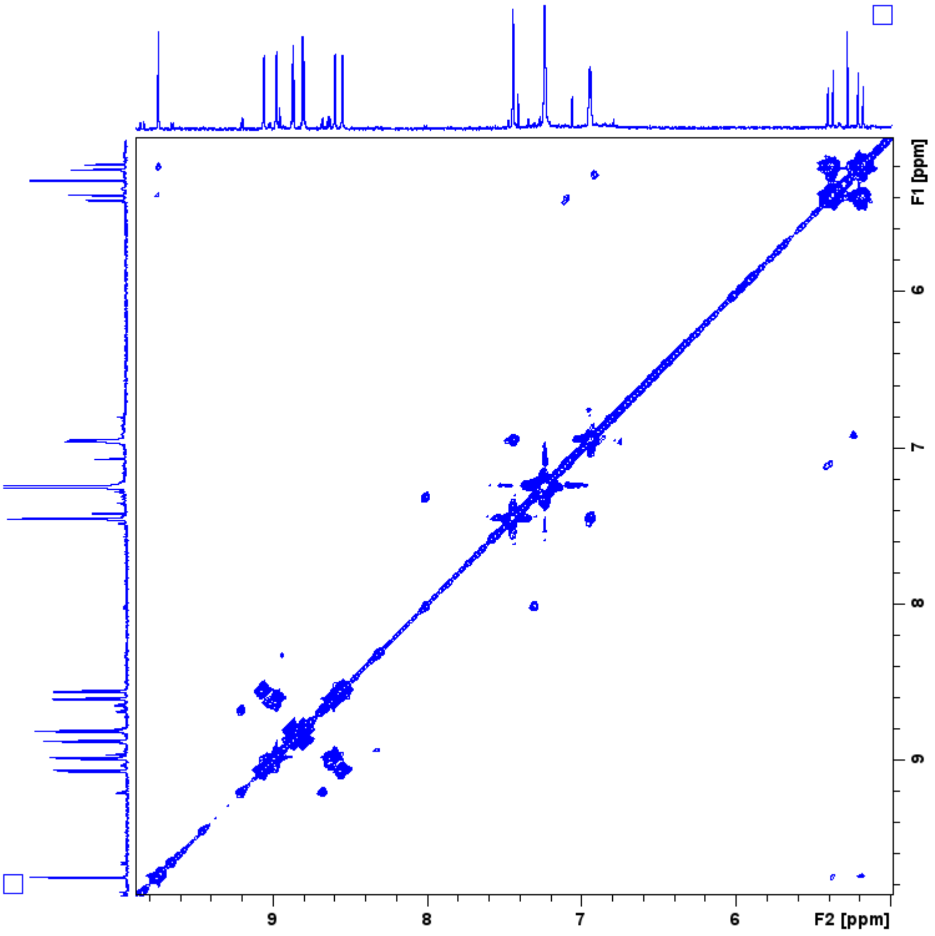

Figure S11. The ^{1}H-^{1}H COSY spectrum of **12** (600 MHz, $CDCl_3$, 300 K).

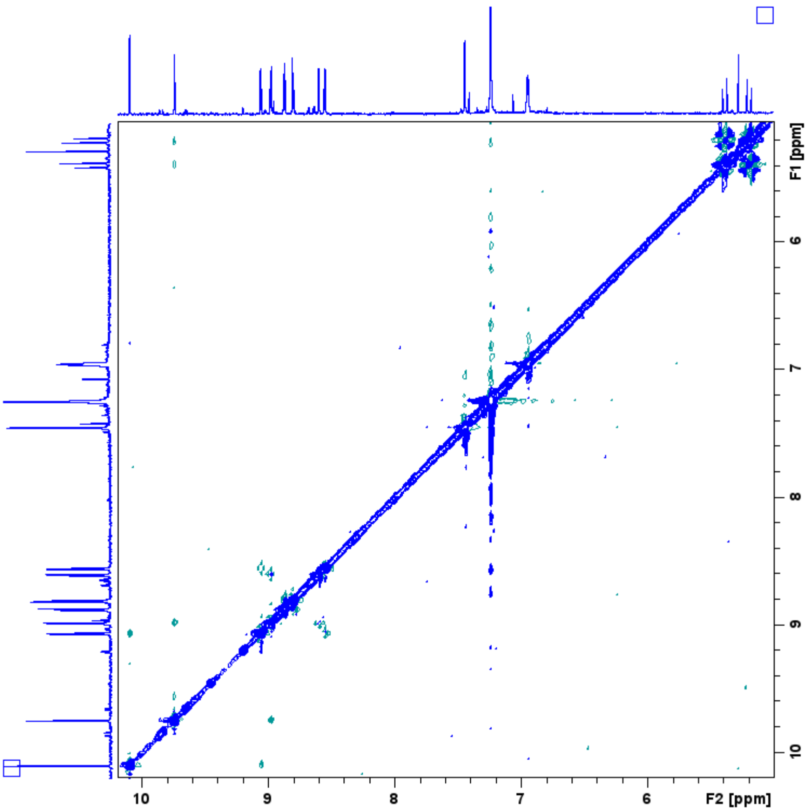

Figure S12. The ^{1}H-^{1}H NOESY spectrum of **12** (600 MHz, $CDCl_3$, 300 K).

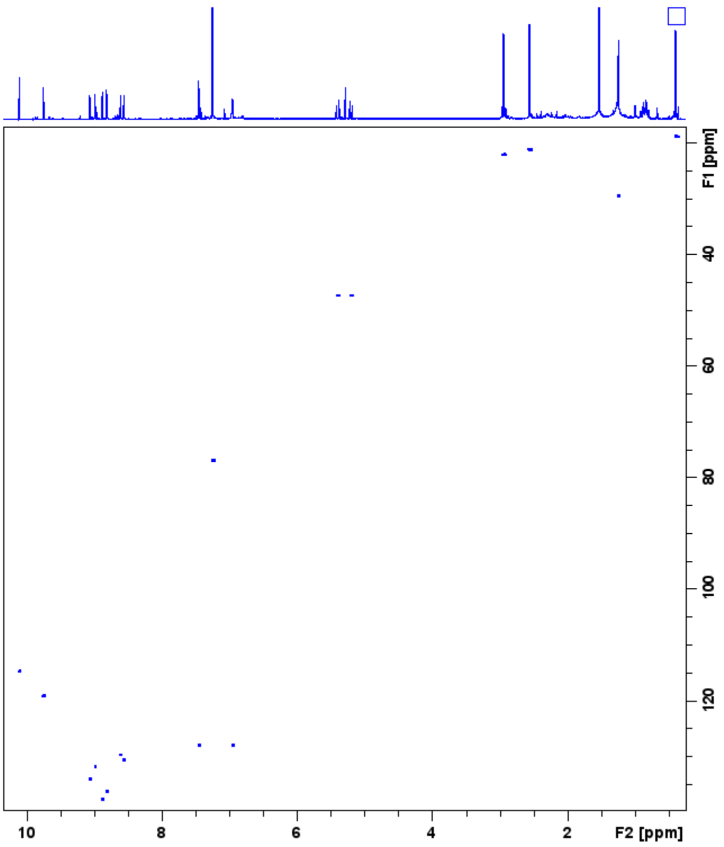

Figure S13. The ^{1}H-^{13}C HSQC spectrum of **12** (600 MHz, $CDCl_3$, 300 K).

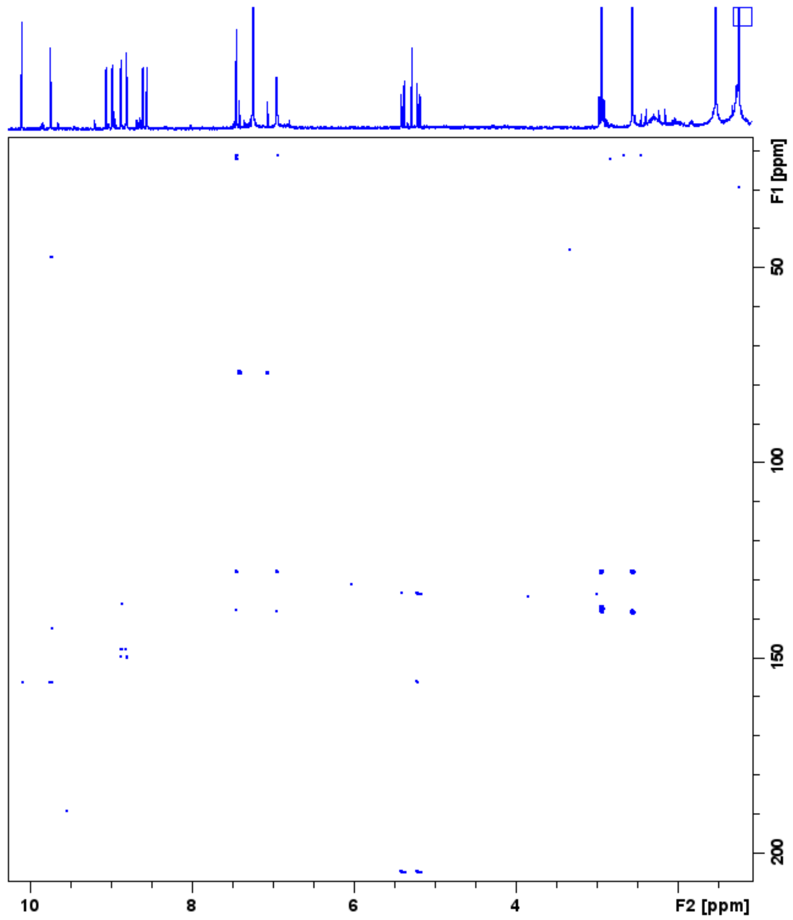

Figure S14. The ^{1}H-^{13}C HMBC spectrum of **12** (600 MHz, $CDCl_3$, 300 K).

NMR spectra of 13

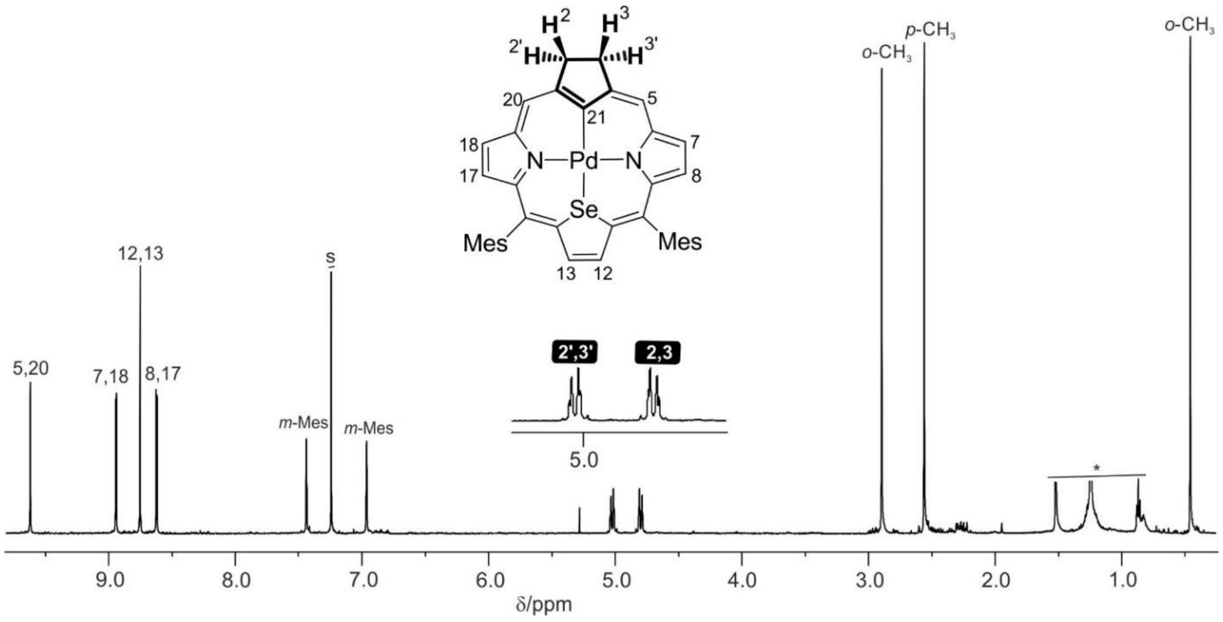

Figure S15. ^{1}H NMR spectrum of **13** (600 MHz, $CDCl_3$, 300 K).

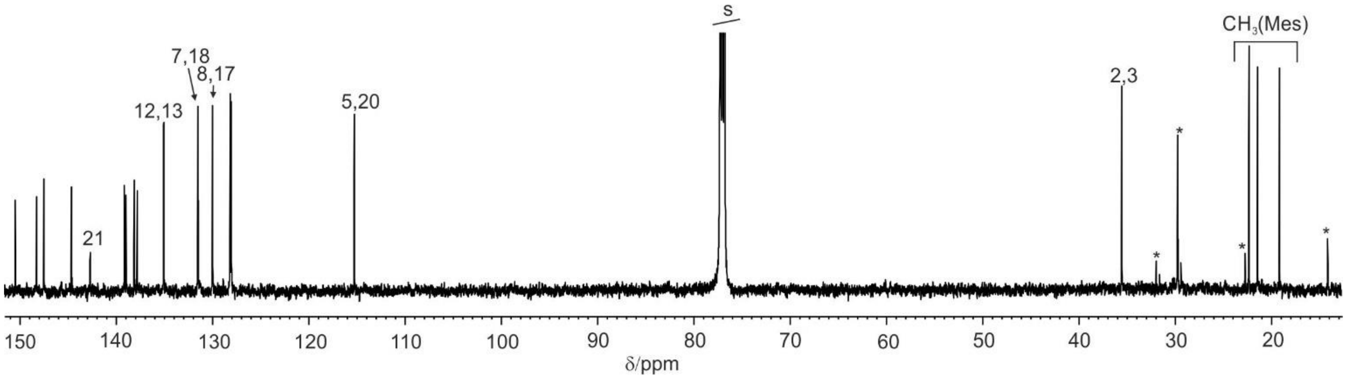

Figure S16. ^{13}C NMR spectrum of **13** (150.9 MHz, $CDCl_3$, 300 K).

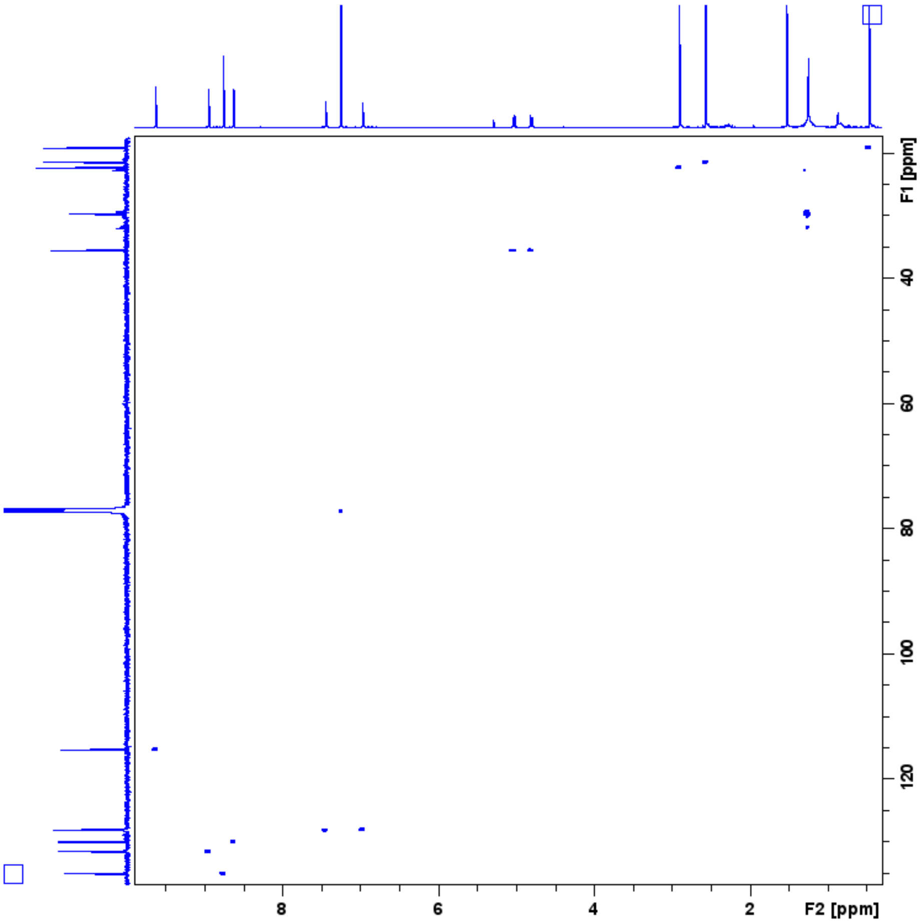

Figure S17. The ^{1}H-^{13}C HSQC spectrum of **13** (600 MHz, $CDCl_3$, 300 K).

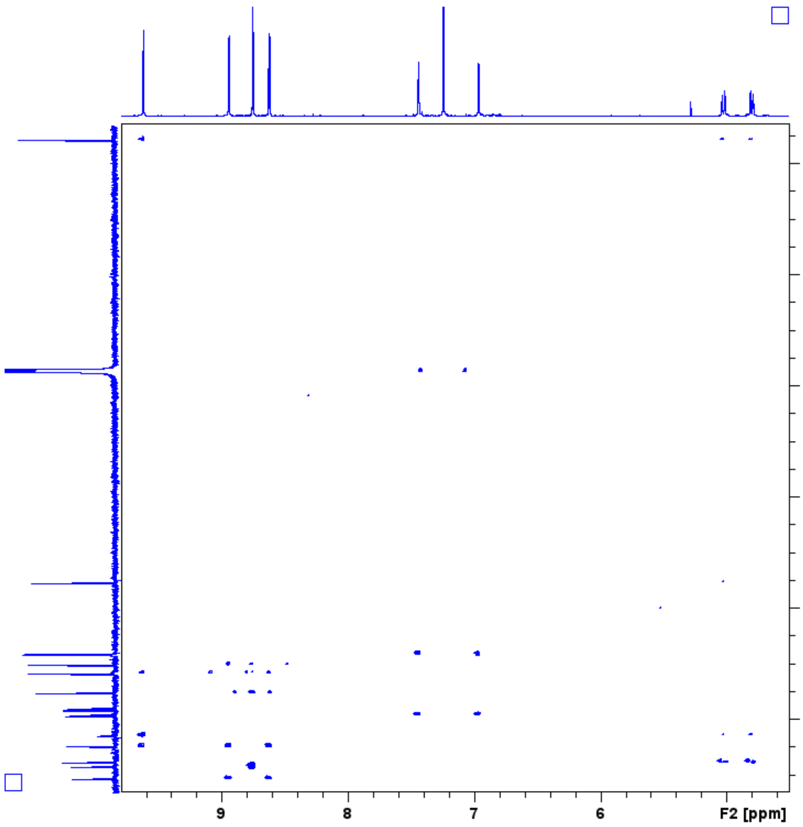

Figure S18. The ^{1}H-^{13}C HMBC spectrum of **13** (600 MHz, $CDCl_3$, 300 K).

NMR spectra of 14

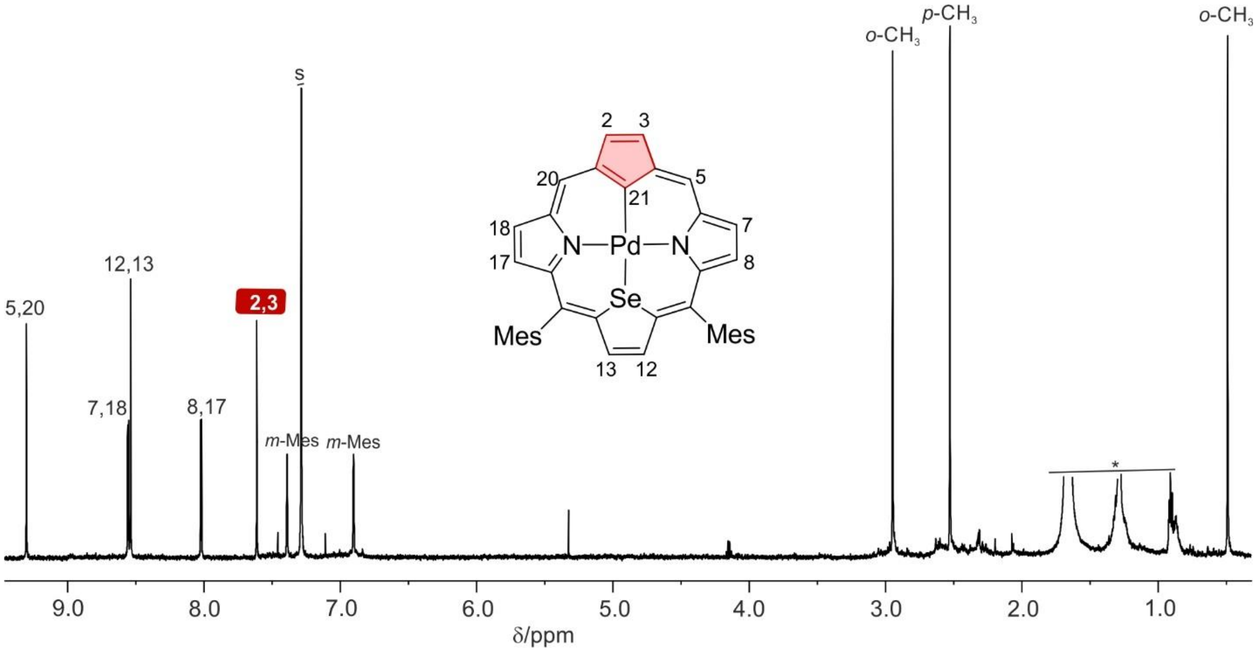

Figure S19. ^{1}H NMR spectrum of **14** (600 MHz, $CDCl_3$, 300 K).

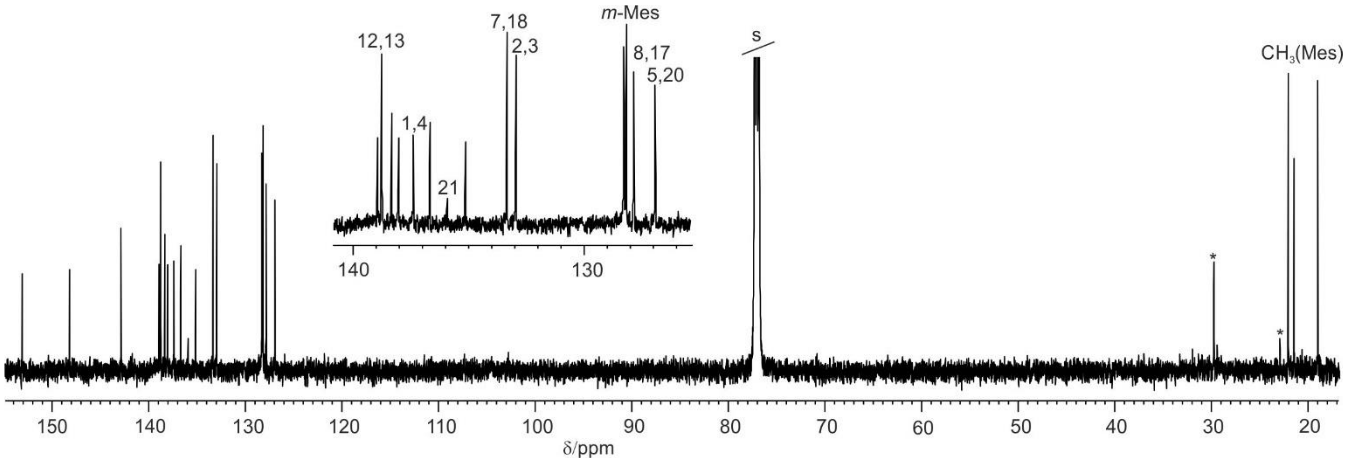

Figure S20. ^{13}C NMR spectrum of **14** (150.9 MHz, $CDCl_3$, 300 K).

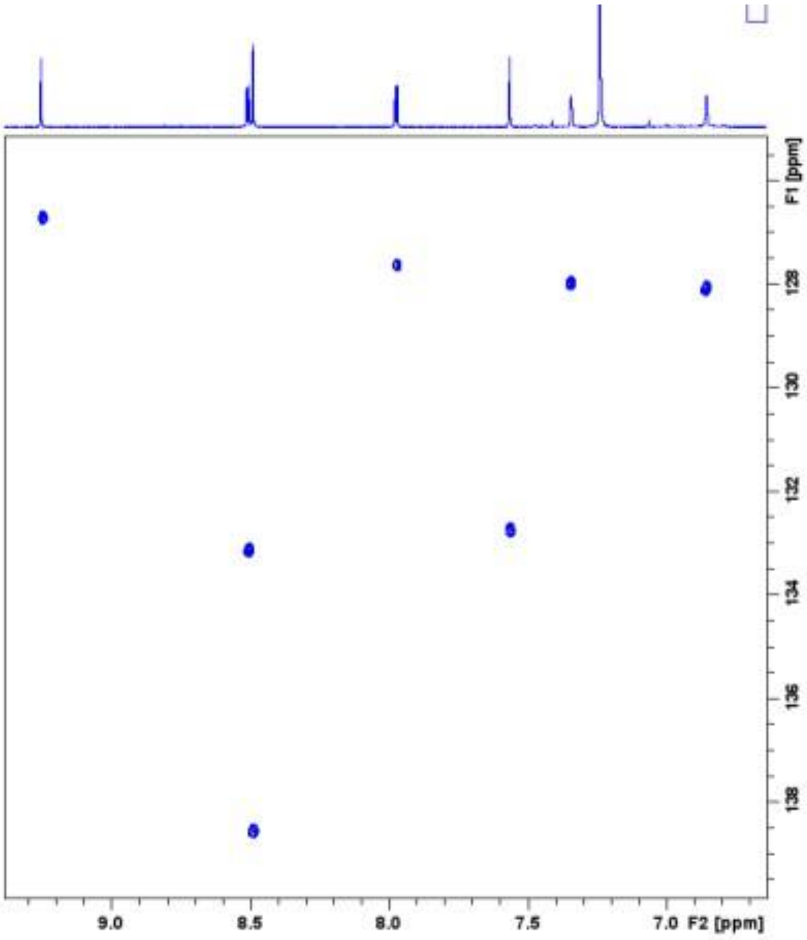

Figure S21. The ^{1}H-^{13}C HSQC spectrum of **14** (600 MHz, $CDCl_3$, 300 K).

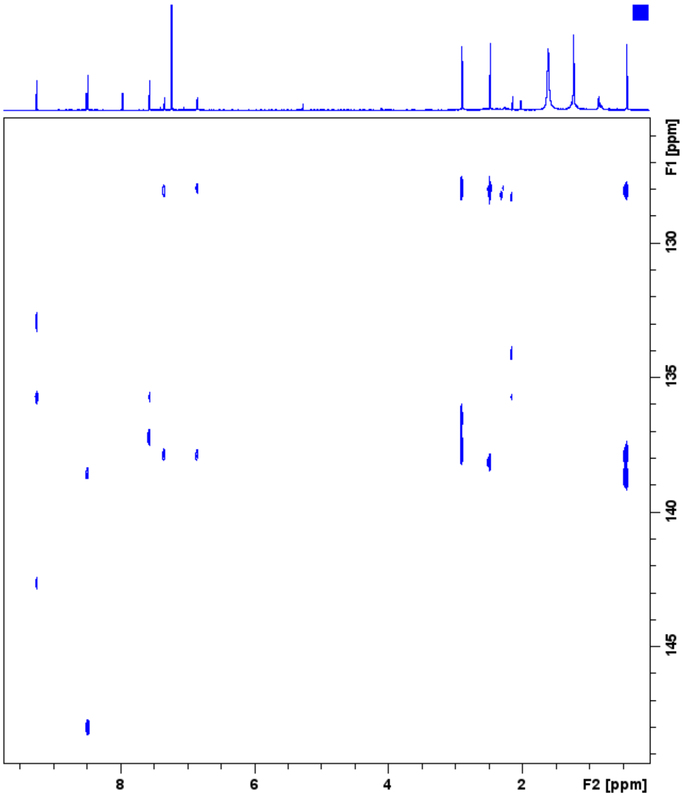

Figure S22. The ^{1}H-^{13}C HMBC spectrum of **14** (600 MHz, $CDCl_3$, 300 K).

NMR spectra of 15

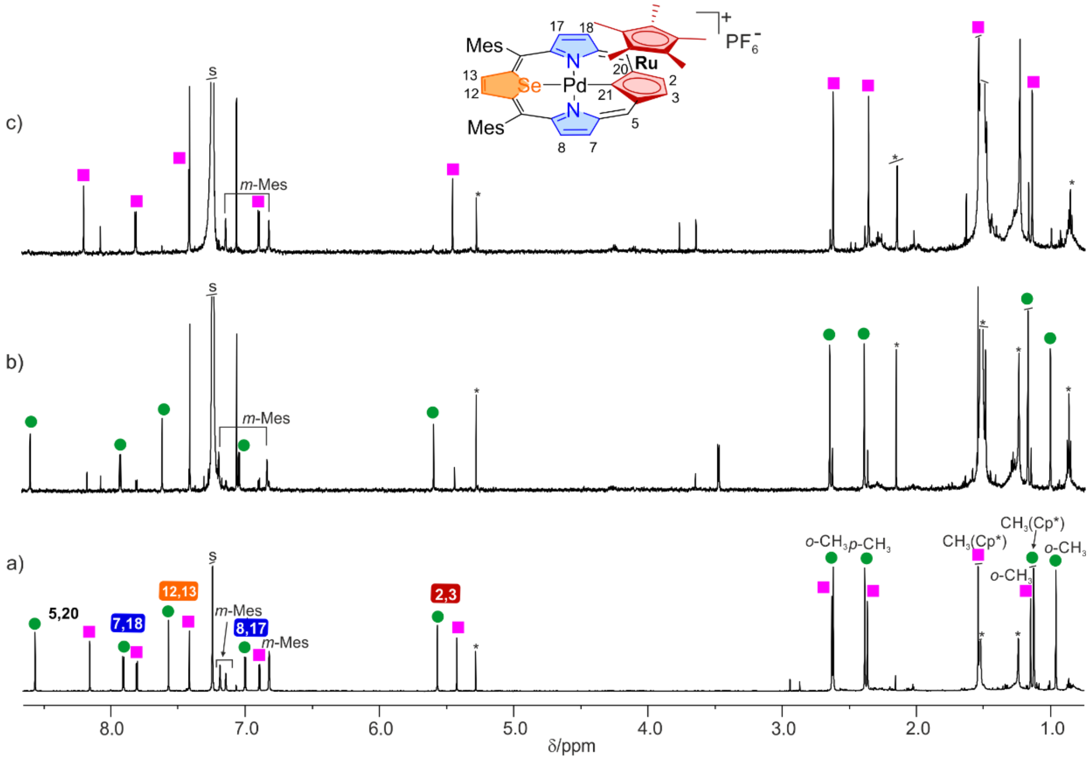

Figure S23. ^{1}H NMR spectra of a) both isomers of **15**, b) **15-A** (green circles), and c) **15-B** (pink circles) (600 MHz, $CDCl_3$, 300 K).

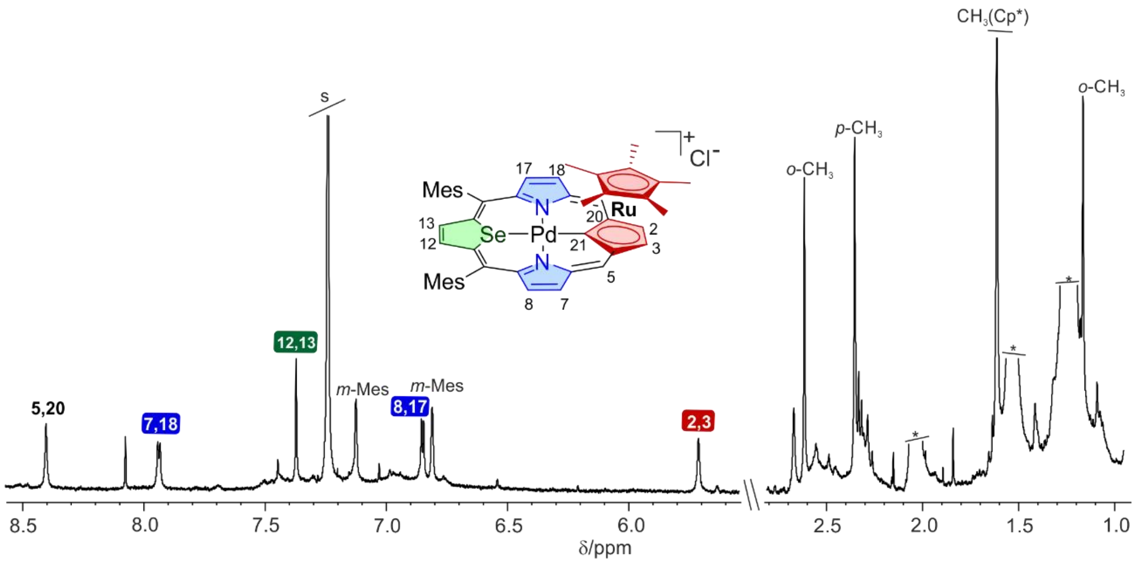

Figure S24. ^{1}H NMR spectrum of **15** directly after synthesis using ruthenocenoselenaporphyrin **11** as a ligand (600 MHz, $CDCl_3$, 300 K).

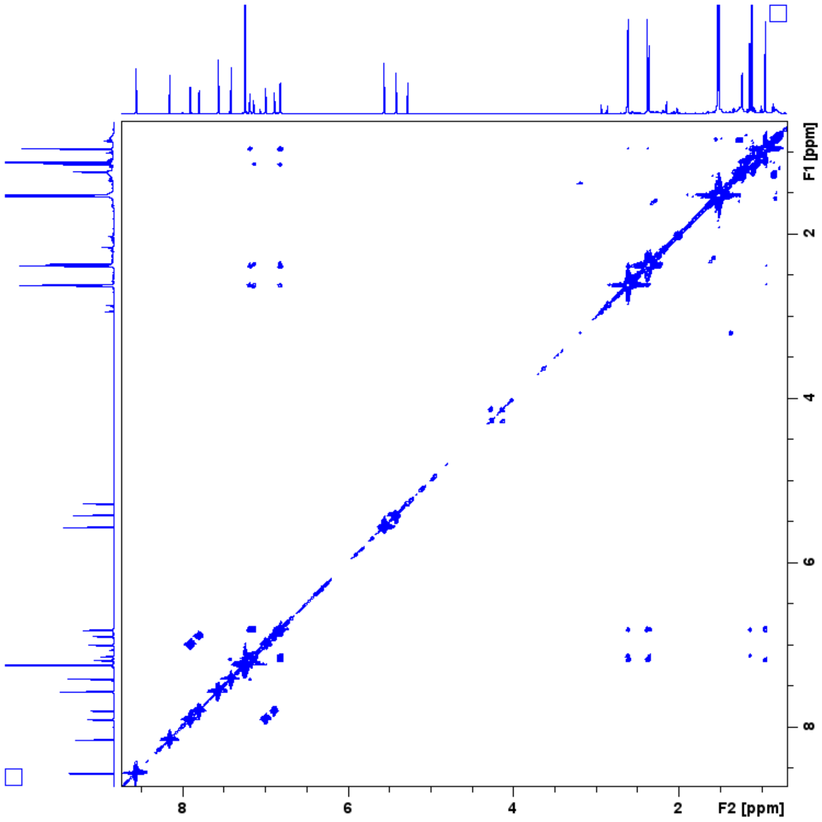

Figure S25. The ^{1}H-^{1}H COSY spectrum of **15** (600 MHz, $CDCl_3$, 300 K).

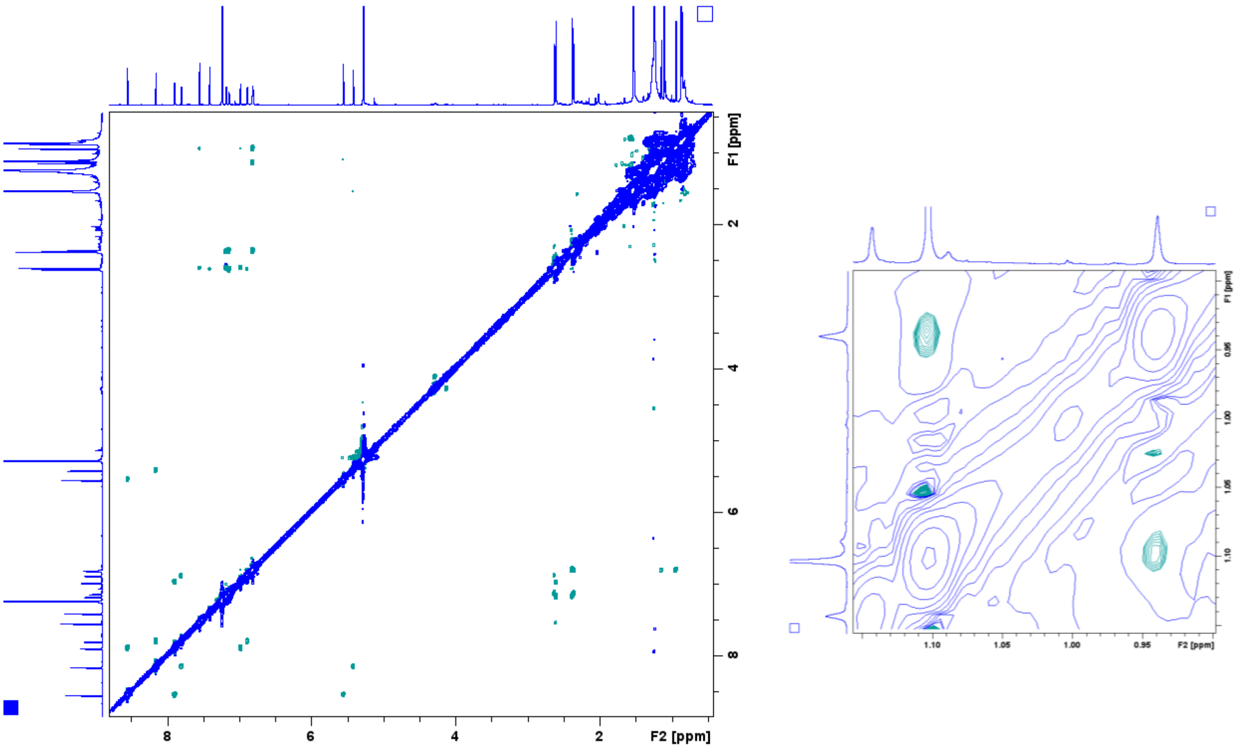

Figure S26. The ^{1}H-^{1}H ROESY spectrum of **15** (600 MHz, $CDCl_3$, 300 K).

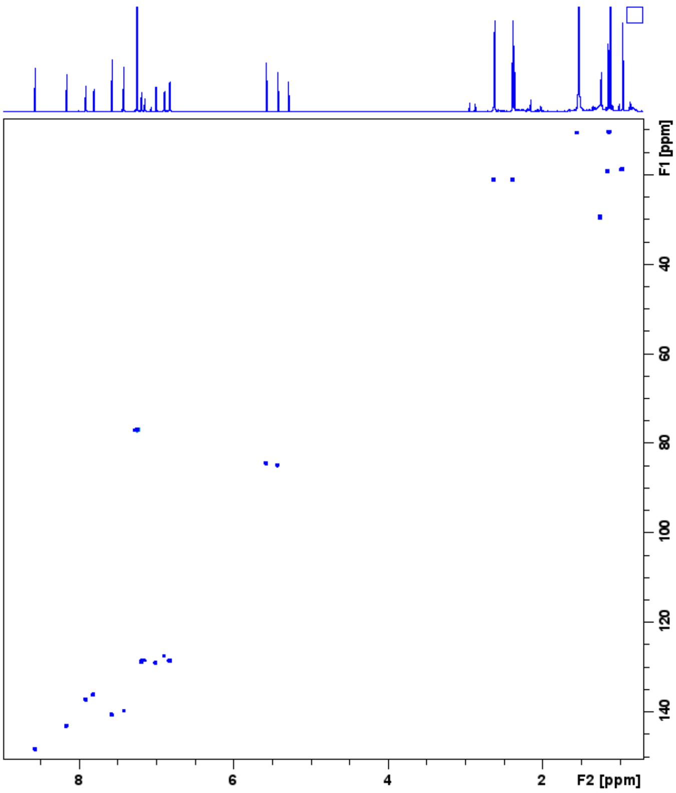

Figure S27. The ^{1}H-^{13}C HSQC spectrum of **15** (600 MHz, $CDCl_3$, 300 K).

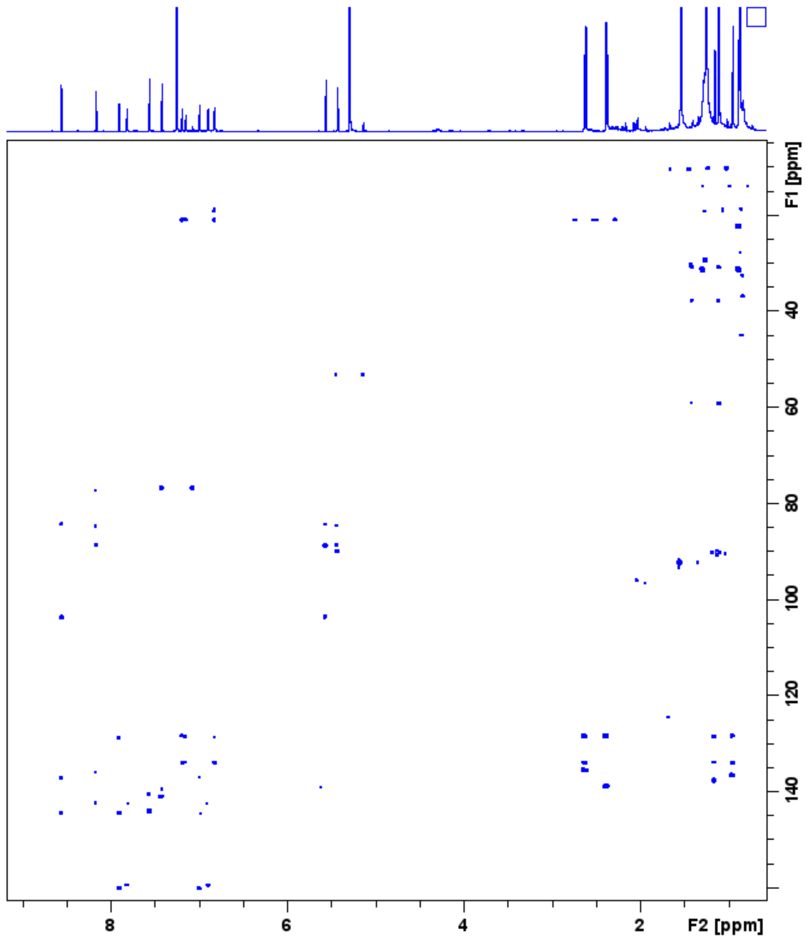

Figure S28. The ^{1}H-^{13}C HMBC spectrum of **15** (600 MHz, $CDCl_3$, 300 K).

4. UV-vis spectra

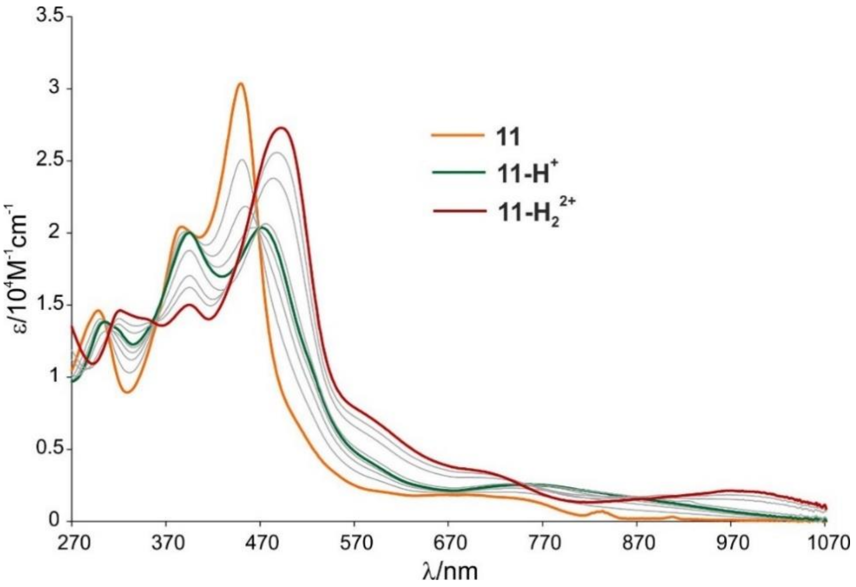

Figure S29. UV-vis titration of **11** with TFA in dichloromethane.

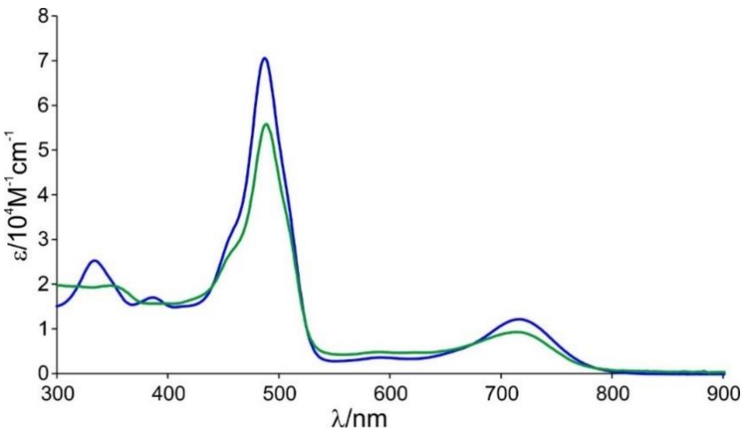

Figure S30. UV-vis spectra of palladium(II) 21-carbaselenachlorins **12** (green) and **13** (blue), in dichloromethane.

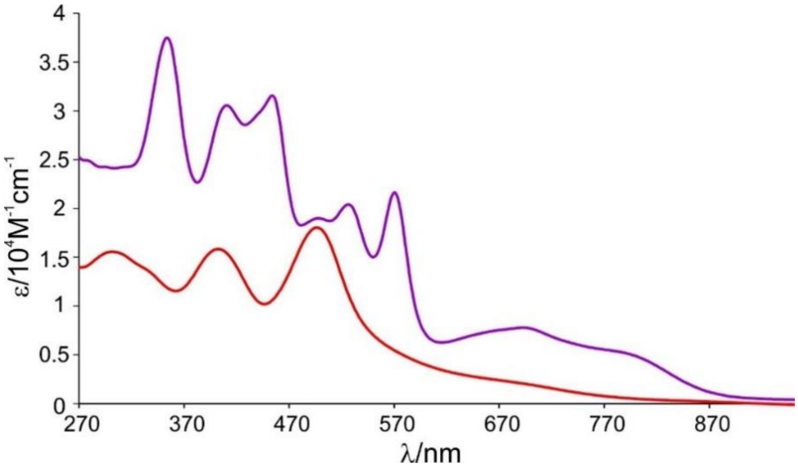

Figure S31. UV-vis spectra of **14** (purple) and **15** (red) as a mixture of both isomers in dichloromethane.

5. MS spectra

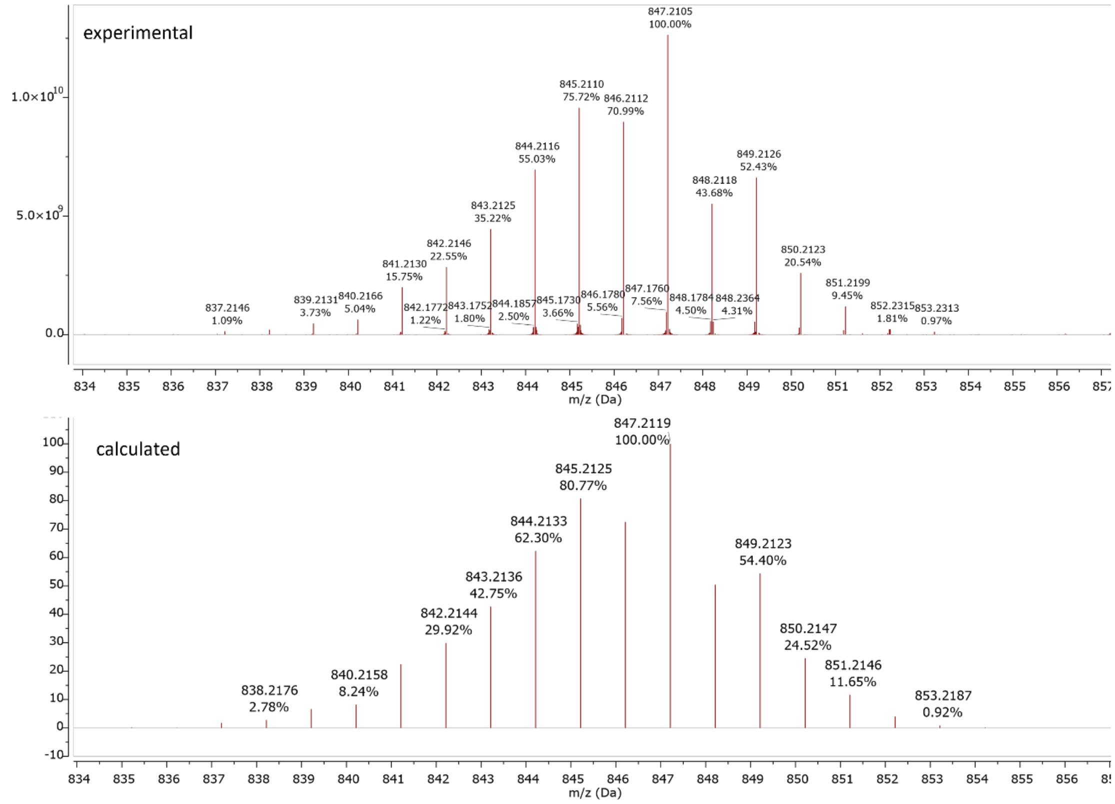

Figure S32. HR-MS spectrum of **11**.

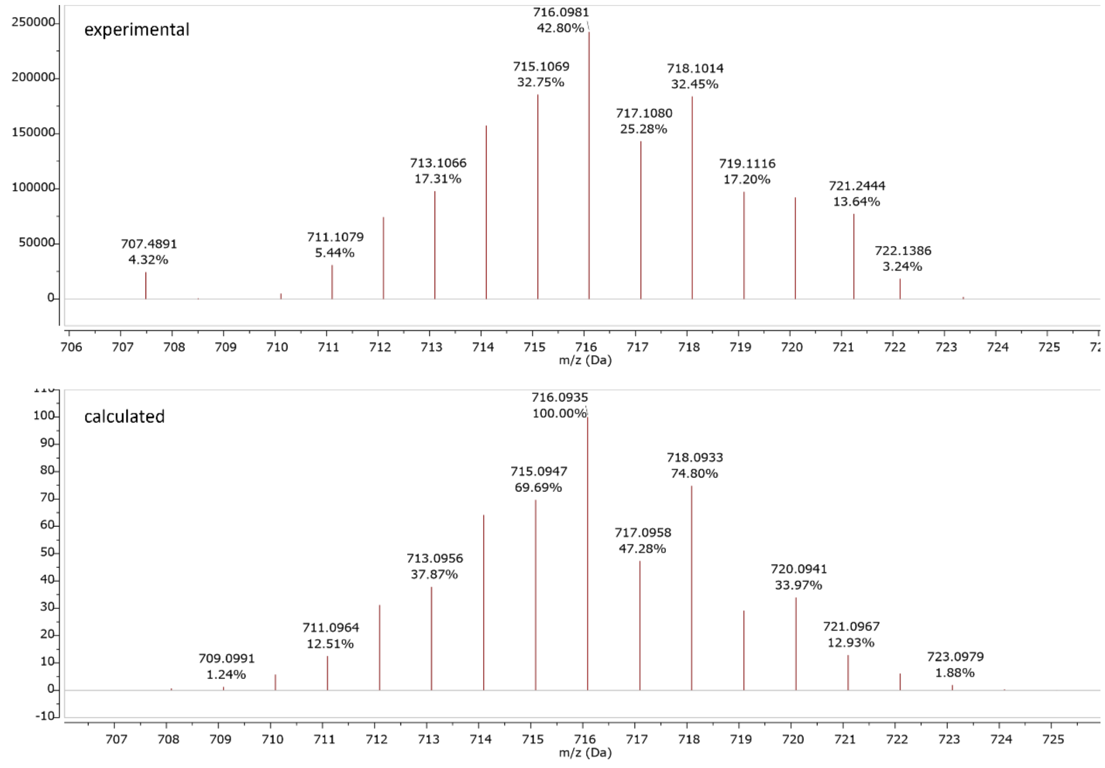

Figure S33. HR-MS spectrum of **12**.

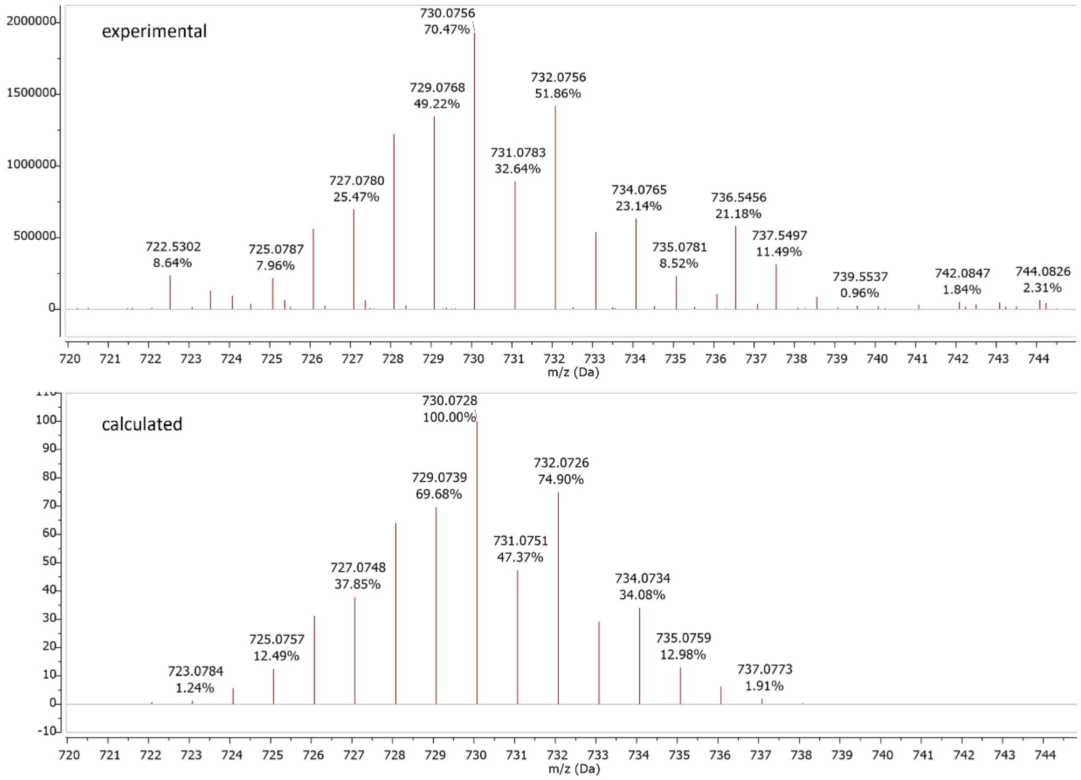

Figure S34. HR-MS spectrum of **13**.

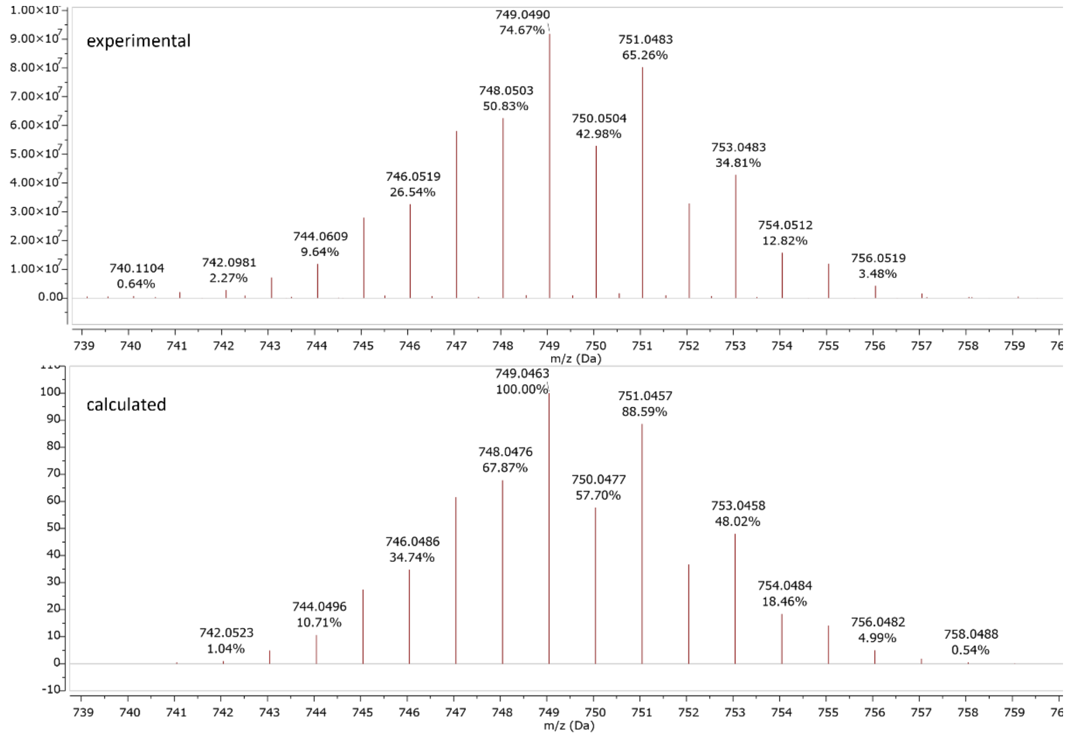

Figure S35. HR-MS spectrum of **14**.

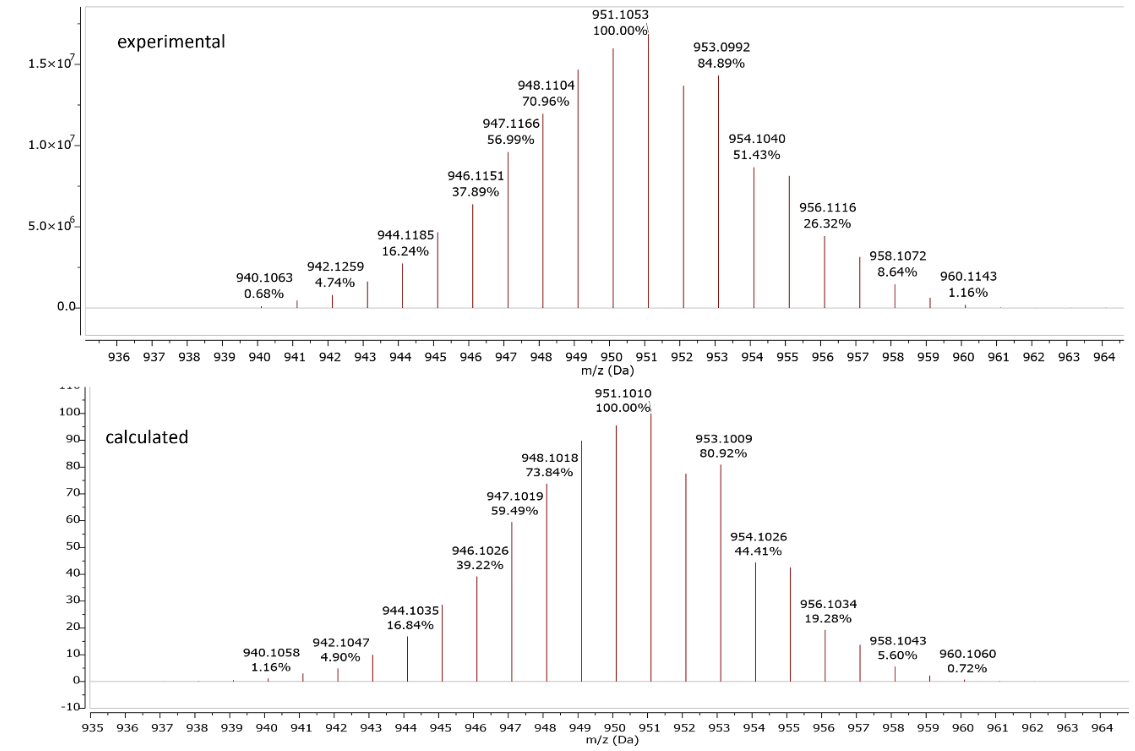

Figure S36. HR-MS spectrum of **15**.

6. DFT calculations

6.1. NICS values

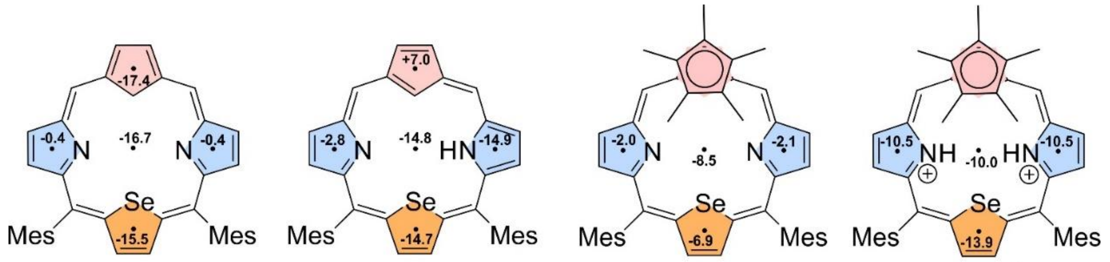

9-I **9-II** **11** **$11\text{-}H_2^{2+}$**

Figure S37. NICS values calculated at the center of each ring for **9-I**,[16] **9-II**,[16] **11** and **$11\text{-}H_2^{2+}$**.

6.2. Optimized structures

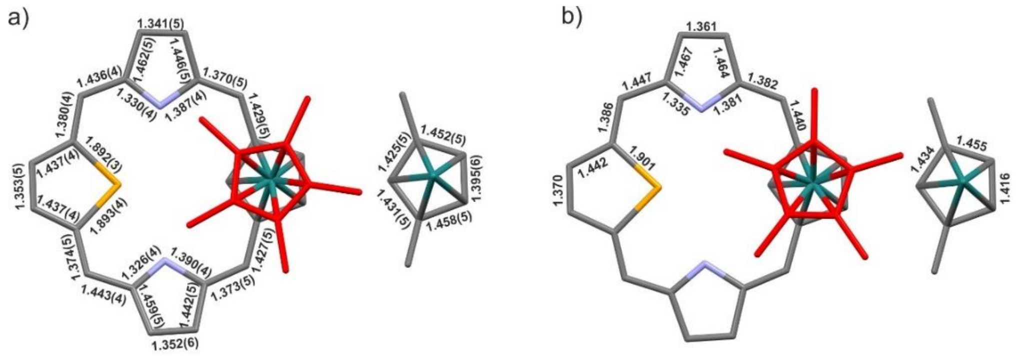

Figure S38. Bond lengths (in Å) for the crystal structure (a) and the DFT-optimized structure (b) of **11**.

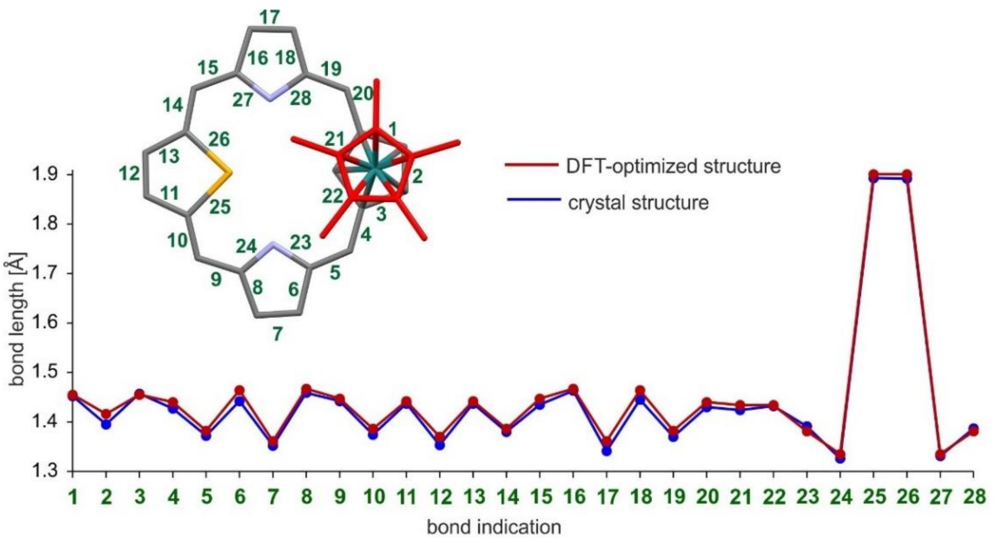

Figure S39. Comparison of bond length in the crystal structure (blue) and the DFT-optimized structure (red) of **11**.

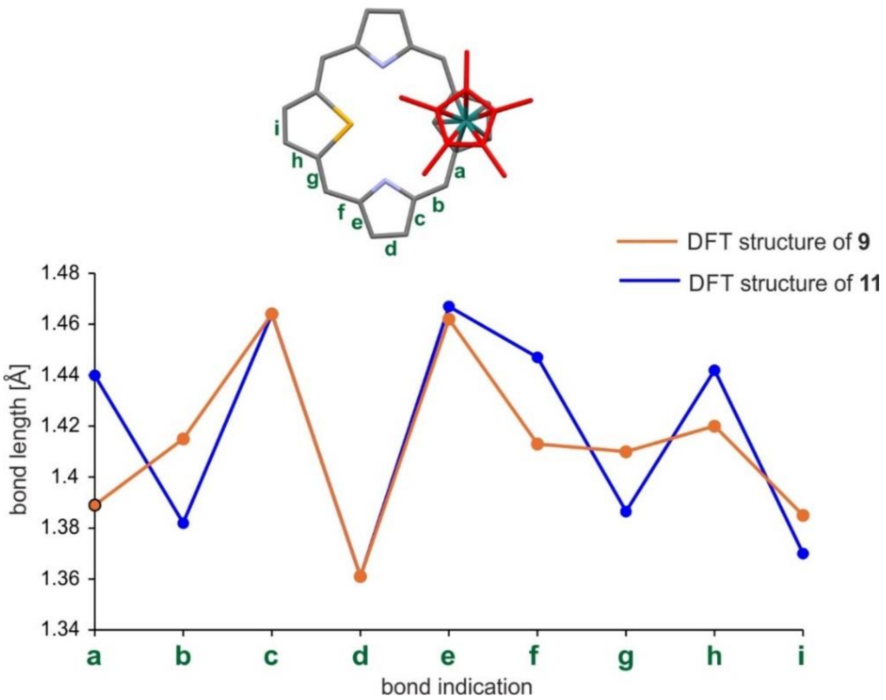

Figure S40. Comparison of bond length in the DFT structures of **9-I** (orange)[16] and **11** (blue).

6.3. The EDDB plots and NICS 2D maps

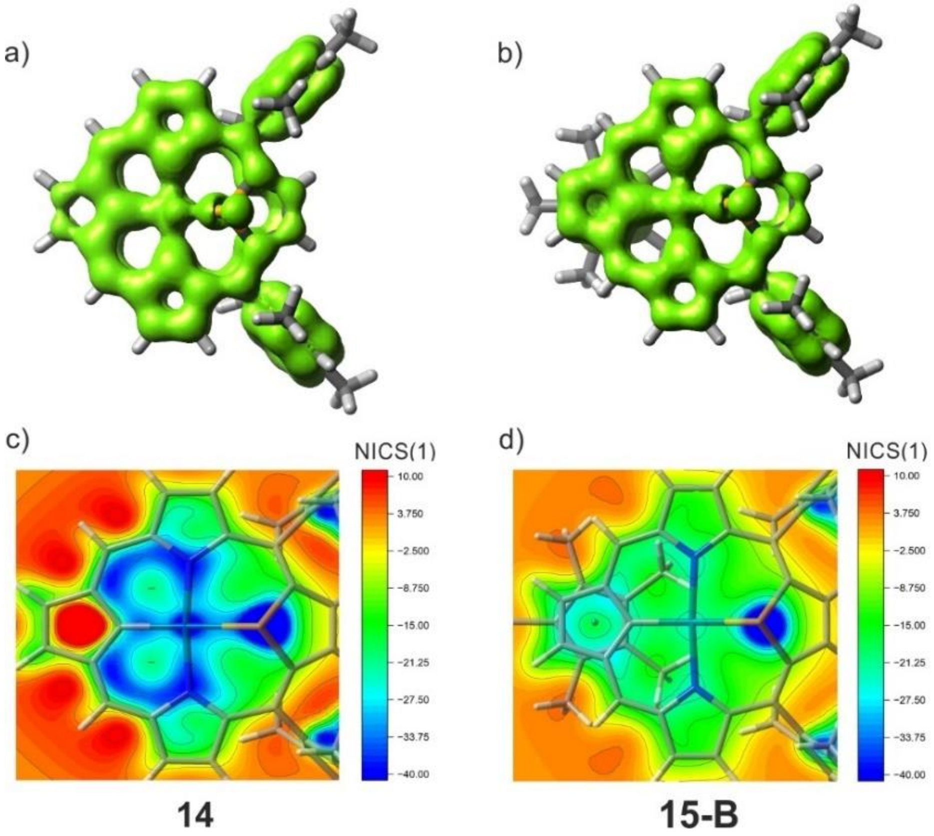

Figure S41. EDDB plots (a,b) and NICS(1)zz 2D maps (c,d) of palladium(II) 21-carba-23-selenaporphyrin **14** (a,c) and ruthenium(II) π complex **15-B** (b,d). In EDDB plots the localized and delocalized cyclic π-conjugation is shown with the green surface with an isovalue of 0.014, while NICS maps are estimated 1 Å above the mean meso plane.

6.4. Correlation between calculated and experimental NMR values

Table S3 ^1H and ^{13}C NMR (selected) chemical shifts experimental and calculated using the GIAO method for **11**.

**Position**	**^1H NMR**		**^{13}C NMR**	
	δ_{exp}(ppm) 300 K, $CDCl_3$	δ_{calc}(ppm)[a]	δ_{exp}(ppm) 300 K, $CDCl_3$	δ_{calc}(ppm)[a]
**1,4**	-	-	89.1	92.5
**2,3**	5.35	5.48	82.4	83.7
**5,20**	7.96	8.15, 8.13 (8.14)	134.9	128.5, 127.7 (128.1)
**7,18**	7.78	7.77, 7.75 (7.76)	137.0	132.3, 131.9 (132.1)
**8,17**	6.97	7.33, 7.31 (7.32)	127.1	122.6
**12,13**	7.61	7.88. 7.86 (7.87)	136.5	128.1
**21**	3.47	1.56	77.3	76.4
***m*-Mes**	7.12	7.18	128.5	123.3
***m'*-Mes**	7.05	7.16	128.5	123.2
***o*-CH_3**	2.18	2.14, 2.11, 1.61 (1.95)	21.3	22.0
***o'*-CH_3**	1.82	2.11, 2.09, 1.61 (1.94)	20.7	21.9
***p*-CH_3**	2.44	2.64, 2.22, 2.18 (2.35)	21.5	21.8
**CH_3(Cp*)**	0.98	2.29, 2.20, 2.14, 1.84, 1.69, 1.60, 1.48, 1.18, 1.13, 0.45, 0.44, 0.10, −0.29, −0.33, −0.42 (1.03)	10.1	13.6, 13.1, 11.5, 10.2, 9.4 (11.6)
**Cp***	-	-	86.5	87.5, 86.2, 86.0, 85.4, 84.8 (86.0)

[a] The average values of calculated chemical shifts are included in brackets

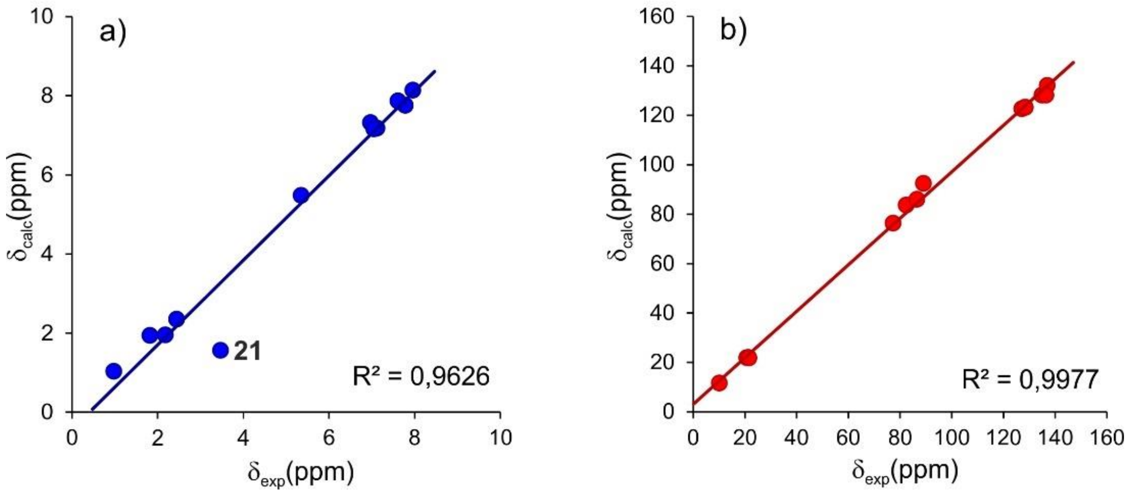

Figure S42. Linear correlation between selected calculated and experimental values of ^1H (a) and ^{13}C (b) chemical shifts for **11**.

Table S4 ^{1}H and ^{13}C NMR (selected) chemical shifts experimental and calculated using the GIAO method for **11-H$_2^{2+}$**.

Position	^{1}H NMR		^{13}C NMR	
	δ_{exp}(ppm) 300 K, CD_2Cl_2	δ_{calc}(ppm)[a]	δ_{exp}(ppm) 300 K, $CDCl_3$	δ_{calc}(ppm)[a]
**2,3**	5.70	5.73	86.8	77.3
**5,20**	8.84	10.04	133.9	126.2
**7,18**	8.37	9.23	135.4	130.9
**8,17**	7.49	8.92, 8.89 (8.91)	126.0	126.7
**12,13**	8.20	9.20, 9.18 (9.19)	137.9	136.9
**21**	1.47	−3.46	73.9	62.2
***m*-Mes**	7.23	7.78	128.4	126.3
***m'*-Mes**	7.14	7.56, 7.54 (7.55)	128.6	126.5
***o*-CH_3**	2.26	2.94, 2.56, 2.07 (2.52)	20.1	21.4
***o'*-CH_3**	1.89	1.94, 1.81, 0.36 (1.37)	20.2	22.8
***p*-CH_3**	2.47	3.00, 2.62, 2.59 (2.74)	21.1	22.2
**CH_3(Cp*)**	1.28	2x3.06, 2x3.04, 2.62, 2x2.19, 2x2.06, 2x1.16, 2x0.49, 2x(-0.10) (1.94)	9.2	2x13.2, 12.9, 2x11.2 (12.3)

[a] The average values of calculated chemical shifts are included in brackets

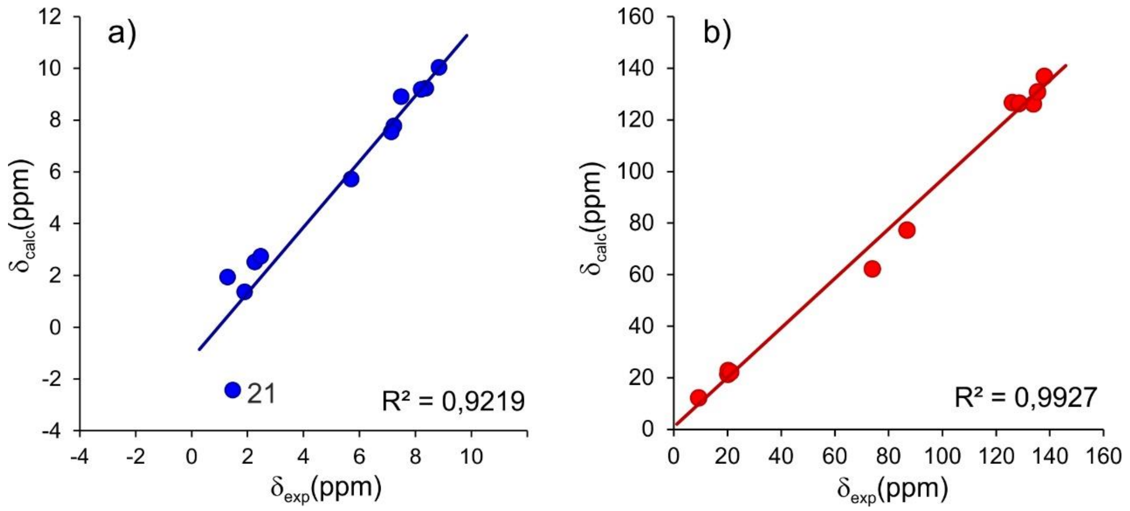

Figure S43. Linear correlation between selected calculated and experimental values of ^{1}H (a) and ^{13}C (b) chemical shifts for **11-H$_2^{2+}$**.

Table S5. ^{1}H and ^{13}C NMR (selected) chemical shifts experimental and calculated for **15-A** using the GIAO method for **15-A**.

Position	^{1}H NMR		^{13}C NMR	
	δ_{exp}(ppm) 300 K, $CHCl_3$	δ_{calc}(ppm)[a]	δ_{exp}(ppm) 300 K, $CHCl_3$	δ_{calc}(ppm)[a]
**1,4**	-	-	89.0	95.3
**2,3**	5.60	5.67	84.4	84.1
**5,20**	8.60	8.65	148.3	139.9
**7,18**	7.93	7.91	137.3	132.8
**8,17**	7.05	7.59	129.0	126.1
**12,13**	7.62	8.25	140.7	135.8
***m*-Mes**	7.20	7.51	128.8	125.1
***m'*-Mes**	6.84	7.11	128.6	125.0
***o*-CH_3**	2.64	3.04, 2.68, 2.32 (2.68)	21.0	22.4
***o'*-CH_3**	1.00	1.31, 1.09, 0.42 (0.94)	18.0	20.0
***p*-CH_3**	2.39	2.72, 2.40, 2.27 (2.46)	21.0	21.7
**CH_3(Cp*)**	1.17	2.26, 2.25, 2.09, 2.07, 1.93, 1.66, 1.65, 1.51, 1.48, 0.79, 0.78, 0.30, 0.27, -0.19, -0.20 (1.24)	10.4	13.0, 2x12.4, 2x10.4 (11.7)
**Cp***	-	-	90.5	95.1, 93.4, 93.3, 2x88.8 (91.9)
**21**	-	-	103.8	126.3

[a] The average values of calculated chemical shifts are included in brackets

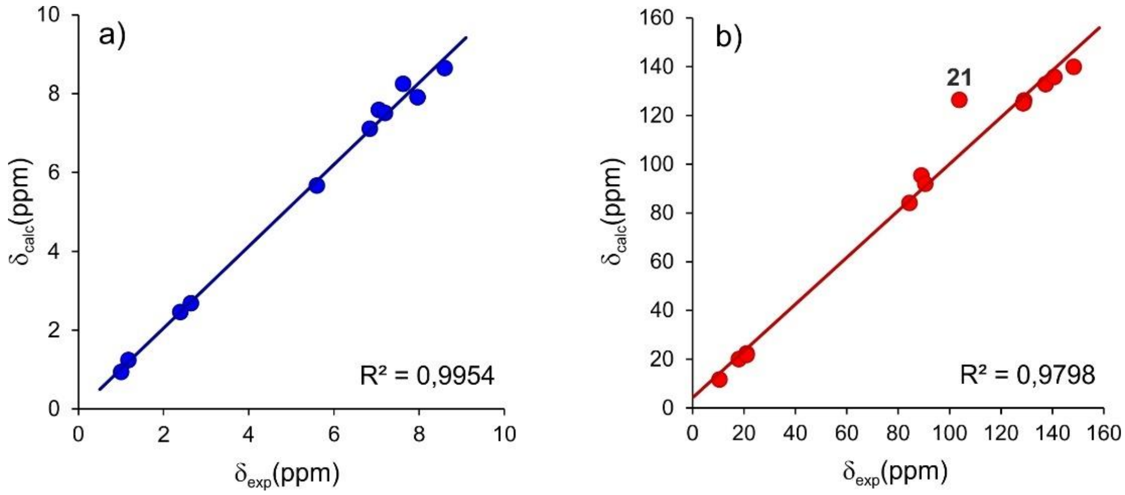

Figure S44. Linear correlation between selected calculated and experimental values of ^{1}H (a) and ^{13}C (b) chemical shifts for **15-A**.

Table S6. ^{1}H and ^{13}C NMR (selected) chemical shifts experimental and calculated using the GIAO method for **15-B**.

Position	^{1}H NMR		^{13}C NMR	
	δ_{exp}(ppm) 300 K, $CHCl_3$	δ_{calc}(ppm)[a]	δ_{exp}(ppm) 300 K, $CHCl_3$	δ_{calc}(ppm)[a]
**1,4**	-	-	90.1	93.5
**2,3**	5.46	5.50	84.9	84.8
**5,20**	8.20	8.51	143.2	137.6
**7,18**	7.81	7.85	136.2	132.4
**8,17**	6.90	7.53	127.6	125.8
**12,13**	7.42	8.11	139.8	135.3
***m*-Mes**	7.14	7.45	128.5	125.1
***m'*-Mes**	6.82	7.12	128.6	125.5
***o*-CH_3**	2.63	3.05, 2.67, 2.31 (2.68)	21.0	22.9
***o'*-CH_3**	1.14	1.30, 1.10, 0.37 (0.93)	19.2	20.1
***p*-CH_3**	2.36	2.69, 2.34, 2.28 (2.44)	21.0	22.0
**CH_3(Cp*)**	1.54	2.35, 2.34, 2.27, 2.24, 2.05, 1.82, 1.79, 1.78, 1.74, 1.07, 1.05, 0.65, 0.58, 0.37, 0.36 (1.50)	10.6	13.2, 13.0, 12.9, 10.2, 10.1 (11.9)
**Cp***	-	-	92.6	95.5, 95.1, 93.4, 89.1, 89.0 (92.4)
**21**	-	-	88.8	110.4

[a] The average values of calculated chemical shifts are included in brackets

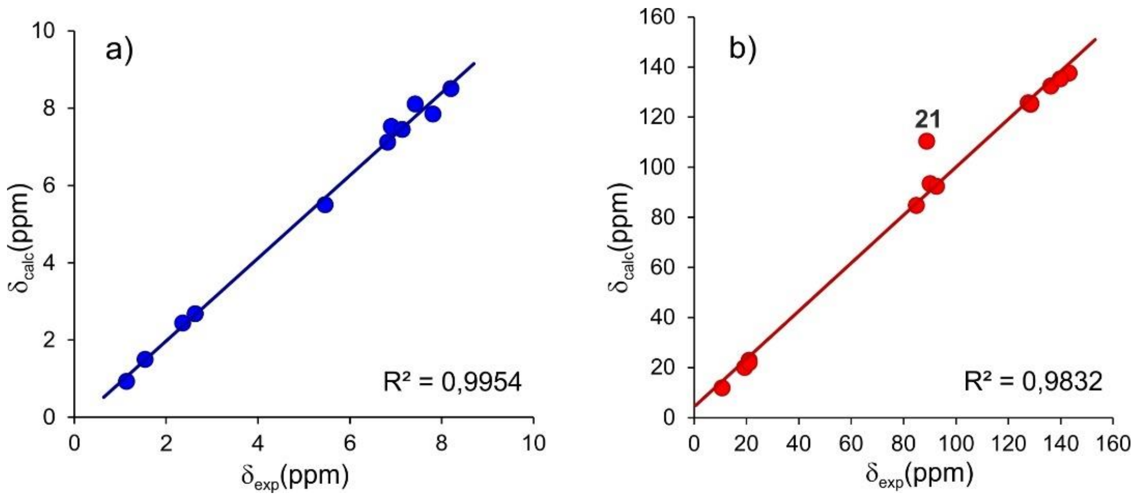

Figure S45. Linear correlation between selected calculated and experimental values of ^{1}H (a) and ^{13}C (b) chemical shifts for **15-B**.

6.5. Cartesian coordinates

11

C	3.53245	-0.69244	0.27046
C	3.5355	0.67777	0.27224
C	2.30558	1.32105	-0.12005
C	2.29945	-1.32904	-0.12286
Se	1.00061	-0.00017	-0.52529
C	2.02717	-2.68429	-0.22858
C	0.72696	-3.14904	-0.66085
C	0.28957	-4.53907	-0.83295
C	-1.0017	-4.48085	-1.25856
C	-1.34916	-3.06098	-1.34708
N	-0.25904	-2.30337	-0.96887
C	-2.57098	-2.54625	-1.7372
C	-2.96197	-1.16597	-1.8569
C	-2.18247	0.00503	-1.57603
C	-2.95577	1.181	-1.85503
C	-4.27823	0.71951	-2.24922
C	-4.28233	-0.69684	-2.2501
C	2.04041	2.67783	-0.22597
C	0.74258	3.14853	-0.65802
C	-1.33407	3.06863	-1.34498
N	-0.2502	2.30683	-0.95661
C	0.31544	4.53947	-0.84463
C	-0.97619	4.48631	-1.27006
C	-2.55729	2.55923	-1.73733
C	3.1034	3.68799	0.09681
C	3.98716	4.13185	-0.91008
C	4.96786	5.07631	-0.58757
C	5.09679	5.59718	0.70302
C	4.21291	5.14302	1.68601
C	3.2178	4.2001	1.40688
C	3.08438	-3.69958	0.09729
C	3.20383	-4.19975	1.41139
C	4.18878	-5.1533	1.69114
C	5.06079	-5.62546	0.70621
C	4.92224	-5.12149	-0.59021
C	3.95132	-4.16744	-0.91352
C	3.88942	3.59916	-2.32197
C	6.14284	6.64048	1.01989
C	2.28618	3.74039	2.50586
C	2.28315	-3.72284	2.51224
C	6.13832	-6.63128	1.03831
C	3.84085	-3.65535	-2.33207
Ru	-3.90415	0.00724	-0.13156
C	-3.66533	0.96188	1.85359
C	-3.29375	-0.42088	1.95175
C	-4.45741	-1.21516	1.62364
C	-5.54807	-0.31933	1.33352
C	-5.05634	1.02736	1.458
C	-2.77383	2.12909	2.15948
C	-1.95891	-0.94967	2.38975
C	-6.96772	-0.71585	1.04868
C	-5.87676	2.27876	1.33576
C	-4.53379	-2.71272	1.687
H	4.40363	-1.28054	0.54191
H	4.40923	1.26124	0.54553
H	0.89299	-5.41894	-0.65393
H	-1.6594	-5.30765	-1.49772
H	-3.33774	-3.27459	-1.99557
H	0.92558	5.41665	-0.67552
H	-1.6273	5.31551	-1.5188
H	-3.31821	3.29095	-2.00368
H	5.64873	5.41214	-1.36707
H	4.29886	5.53096	2.69901
H	4.27393	-5.53758	2.7056
H	5.58575	-5.48076	-1.37423
H	2.89975	3.78493	-2.75321
H	4.04651	2.5158	-2.35491
H	4.63468	4.06981	-2.96896
H	5.75272	7.65452	0.86476
H	7.02401	6.53158	0.38019
H	6.47034	6.57594	2.06212
H	2.51585	4.24435	3.44866
H	2.36111	2.6602	2.67057
H	1.23966	3.9483	2.2568
H	1.23266	-3.91607	2.26835
H	2.37438	-2.64326	2.67254

H	2.50959	-4.22638	3.45606
H	5.84388	-7.27245	1.87481
H	7.07295	-6.13248	1.32494
H	6.36455	-7.27377	0.18171
H	3.99785	-2.57263	-2.38156
H	2.84739	-3.84688	-2.75207
H	4.58024	-4.13541	-2.97887
H	-1.74497	1.93529	1.84619
H	-2.76634	2.3408	3.23712
H	-3.10603	3.03453	1.64605
H	-7.02213	-1.68763	0.55102
H	-7.46665	0.0137	0.40514
H	-7.54933	-0.78832	1.97773
H	-6.6966	2.15263	0.62349
H	-5.27107	3.12397	0.99896
H	-6.31883	2.5544	2.30274
H	-3.59708	-3.17478	1.36462
H	-5.33324	-3.10069	1.05022
H	-4.73546	-3.04952	2.71269
H	-5.11189	-1.32917	-2.5384
H	-1.15935	0.0016	-1.27036
H	-5.10426	1.35718	-2.53591
H	-1.14556	-0.29077	2.0772
H	-1.9179	-1.05032	3.48291
H	-1.75725	-1.93149	1.95508

11-H_2^{2+}

C	3.88995	-0.69038	0.00255
C	3.88999	0.69048	0.00271
C	2.62922	1.33435	0.17059
C	2.62916	-1.33424	0.17025
Se	1.34286	0.00002	0.61227
C	2.3276	-2.69241	-0.00041
C	0.99571	-3.15178	-0.25306
C	0.47707	-4.48641	-0.20888
C	-0.84511	-4.44902	-0.59238
C	-1.19309	-3.08671	-0.88259
N	-0.04766	-2.34864	-0.64251
C	-2.39498	-2.55085	-1.33234
C	-2.71525	-1.17658	-1.5243
C	-2.08333	0.00025	-0.97828
C	-2.71502	1.17712	-1.52458
C	-3.85594	0.70398	-2.3064
C	-3.85607	-0.70338	-2.30625
C	2.32777	2.69262	0.00025
C	0.99606	3.15212	-0.25294
C	-1.19259	3.08717	-0.88305
N	-0.04736	2.34902	-0.64234
C	0.47764	4.48685	-0.20955
C	-0.84446	4.44952	-0.59337
C	-2.39448	2.55134	-1.33295
C	3.43048	3.69692	0.02331
C	3.80076	4.37501	-1.16589
C	4.83925	5.30608	-1.1086
C	5.50931	5.60325	0.08444
C	5.12372	4.92007	1.24374
C	4.10599	3.96401	1.24145
C	3.43015	-3.69691	0.02279
C	4.1015	-3.96775	1.24238
C	5.11291	-4.93055	1.24718
C	5.49977	-5.61306	0.08789
C	4.83005	-5.31628	-1.10541
C	3.79785	-4.37844	-1.16521
C	3.14043	4.09089	-2.49858
C	6.59943	6.64336	0.12313
C	3.74151	3.27593	2.54013
C	3.73391	-3.28163	2.54121
C	6.62253	-6.61823	0.11694
C	3.13664	-4.0966	-2.49794
Ru	-4.23643	0.0005	-0.19935
C	-4.77271	0.71309	1.86062
C	-4.77257	-0.71261	1.86049
C	-5.76329	-1.1604	0.89272
C	-6.40039	0.00022	0.33167
C	-5.76354	1.16087	0.89295
C	-3.97436	1.60539	2.76231
C	-3.97404	-1.60489	2.76203
C	-7.56471	0.00021	-0.6128
C	-6.16806	2.58458	0.65675

C	-6.1675	-2.58414	0.65618
H	4.77839	-1.27525	-0.20823
H	4.7785	1.27533	-0.20792
H	1.04876	-5.35082	0.09623
H	-1.52985	-5.28447	-0.64858
H	-3.15574	-3.27157	-1.61953
H	1.04941	5.35127	0.09531
H	-1.52906	5.28506	-0.65017
H	-3.15498	3.27213	-1.62062
H	5.14026	5.8098	-2.0235
H	5.63021	5.14055	2.17979
H	5.611	-5.15911	2.18575
H	5.12426	-5.82824	-2.01801
H	2.16155	4.57687	-2.58448
H	2.98851	3.01938	-2.66446
H	3.75711	4.46711	-3.31762
H	6.17797	7.63645	0.32159
H	7.13245	6.70159	-0.82954
H	7.32568	6.4349	0.91307
H	4.16479	3.81683	3.38906
H	4.12892	2.25158	2.58493
H	2.65863	3.219	2.68932
H	2.65074	-3.22829	2.69005
H	4.11781	-2.25601	2.58648
H	4.15867	-3.82137	3.39012
H	6.71257	-7.09108	1.0983
H	7.58154	-6.13204	-0.10029
H	6.47926	-7.40169	-0.63195
H	2.98277	-3.02547	-2.66426
H	2.15869	-4.58451	-2.58353
H	3.75401	-4.47197	-3.31686
H	-3.04962	1.12662	3.09208
H	-4.55595	1.85069	3.6598
H	-3.71552	2.5482	2.27466
H	-3.04946	-1.12596	3.09197
H	-3.7149	-2.54753	2.27418
H	-4.55563	-1.85056	3.65941
H	-7.56974	-0.88394	-1.25407
H	-7.5697	0.88429	-1.25415
H	-8.50464	0.00025	-0.04711
H	-6.56793	2.73142	-0.34918
H	-5.33209	3.2736	0.7983
H	-6.95384	2.874	1.36583
H	-5.33155	-3.27307	0.79825
H	-6.5667	-2.73097	-0.35003
H	-6.95372	-2.87368	1.36473
H	-4.52573	-1.33896	-2.86977
H	-1.28829	0.00023	-0.25147
H	-4.52548	1.33958	-2.87005
H	0.07467	-1.39868	-0.96083
H	0.0749	1.39887	-0.96014

14

C	-0.67687	5.90686	0.87965
C	-1.14263	4.54047	0.55274
C	0.67689	5.90686	0.87963
C	1.14264	4.54046	0.55273
C	0.0	3.72744	0.37132
C	-2.45845	4.12954	0.38495
C	-2.89791	2.83447	0.05103
C	-4.27223	2.43599	0.01326
C	-4.29215	1.08216	-0.19773
C	-2.92819	0.64376	-0.31029
C	-2.57813	-0.75792	-0.36097
C	-1.3255	-1.2623	-0.64904
C	-0.69137	-2.48225	-0.25352
C	0.69137	-2.48225	-0.25352
C	1.3255	-1.2623	-0.64905
C	2.57813	-0.75793	-0.36099
C	2.92819	0.64375	-0.31031
C	4.29215	1.08216	-0.19776
C	4.27224	2.43599	0.01321
C	2.89791	2.83446	0.05101
C	2.45846	4.12954	0.38493
N	-2.09386	1.74383	-0.18329
N	2.09386	1.74382	-0.1833
C	3.63169	-1.7472	0.05916
C	5.58979	-3.62788	0.83781
C	5.26375	-3.44512	-0.50891

C	4.29169	-2.52533	-0.9169
C	6.61551	-4.65439	1.25757
C	-3.63169	-1.74719	0.05917
C	-4.29174	-2.52527	-0.9169
C	-5.2638	-3.44506	-0.50891
C	-5.58979	-3.62787	0.83781
C	-6.61552	-4.65438	1.25757
H	-3.24989	4.86029	0.54124
H	-5.11497	3.10073	0.15231
H	-5.15228	0.43216	-0.26621
H	-1.27117	-3.29952	0.16242
H	1.27116	-3.29952	0.16242
H	5.15228	0.43215	-0.26625
H	5.11498	3.10073	0.15223
H	3.24991	4.86029	0.54121
H	7.14308	-4.34566	2.16521
H	6.14375	-5.62218	1.4701
H	7.35911	-4.82169	0.47258
H	-5.78009	-4.0323	-1.26536
H	-7.14294	-4.34576	2.16534
H	-7.35924	-4.82152	0.47266
H	-6.14379	-5.62224	1.46988
C	-4.92057	-2.85028	1.78748
C	-3.95018	-1.91016	1.42581
H	-5.15998	-2.976	2.84134
C	3.95023	-1.91012	1.42579
C	4.9206	-2.85025	1.78747
H	5.16005	-2.97593	2.84132
H	5.78001	-4.03239	-1.26535
C	3.98322	-2.36999	-2.3901
C	3.26594	-1.09232	2.49796
C	-3.98333	-2.36987	-2.39009
C	-3.26583	-1.09241	2.49797
Se	0.0	-0.21791	-1.5284
H	4.69482	-2.93647	-2.99666
H	2.97704	-2.729	-2.63278
H	4.0291	-1.32174	-2.70313
H	3.49789	-1.48433	3.49187
H	3.58691	-0.04524	2.46591
H	2.17829	-1.09279	2.37603
H	-4.69485	-2.93644	-2.99665
H	-2.9771	-2.72873	-2.63281
H	-4.02936	-1.32162	-2.70311
H	-3.49795	-1.4843	3.4919
H	-3.58659	-0.04527	2.46582
H	-2.17817	-1.09311	2.37615
Pd	0.0	1.86042	-0.26759
H	-1.33052	6.75007	1.07258
H	1.33054	6.75007	1.07255

15-A

C	-4.35817	-0.70548	-1.9934
C	-3.00383	-1.15618	-1.68745
C	-4.35827	0.70513	-1.99322
C	-3.00397	1.15587	-1.68716
C	-2.19355	-0.00013	-1.43983
C	-2.54487	-2.49817	-1.64926
C	-1.24096	-2.91295	-1.44346
C	-0.83314	-4.29222	-1.35088
C	0.49011	-4.29624	-1.02816
C	0.91788	-2.91788	-0.93598
C	2.24312	-2.56607	-0.447
C	2.81292	-1.32022	-0.5515
C	3.81801	-0.6879	0.25954
C	3.81801	0.68793	0.25956
C	2.81286	1.32024	-0.55141
C	2.24292	2.56602	-0.44674
C	0.91759	2.91778	-0.93542
C	0.48961	4.29613	-1.02704
C	-0.83366	4.29205	-1.3496
C	-1.24132	2.91276	-1.44263
C	-2.54517	2.49793	-1.64857
N	-0.13959	-2.09249	-1.21456
N	-0.13982	2.09235	-1.21413
C	2.99387	3.61637	0.32313
C	4.42034	5.58436	1.75647
C	4.77263	5.25231	0.44449
C	4.08762	4.27461	-0.28378
C	5.20136	6.61716	2.53223

C	2.99405	-3.61634	0.32296
C	4.08889	-4.27345	-0.28325
C	4.77412	-5.25082	0.44524
C	4.42101	-5.58365	1.75681
C	5.20234	-6.61599	2.53286
H	-3.27608	-3.28658	-1.8109
H	-1.48518	-5.14194	-1.50444
H	1.13483	-5.1466	-0.86228
H	4.42881	-1.27258	0.93873
H	4.42882	1.2726	0.93874
H	1.13426	5.1465	-0.86091
H	-1.48584	5.14173	-1.50281
H	-3.27644	3.28631	-1.81007
H	4.57838	7.1096	3.28415
H	6.04525	6.15543	3.05953
H	5.61317	7.38625	1.87265
H	5.60632	-5.76716	-0.02716
H	4.57984	-7.10756	3.28574
H	5.61335	-7.38582	1.87362
H	6.04685	-6.15407	3.059
C	3.33809	-4.91456	2.3361
C	2.61325	-3.93989	1.6444
H	3.04775	-5.15805	3.35541
C	2.61397	3.93902	1.64505
C	3.33853	4.91408	2.33648
H	3.04886	5.15693	3.35613
H	5.60398	5.76954	-0.02843
C	4.51844	3.96388	-1.70104
C	1.45216	3.24995	2.32551
C	4.52077	-3.96185	-1.7
C	1.45011	-3.25236	2.32412
Se	2.17314	0.00004	-1.7699
H	5.26609	4.68328	-2.04288
H	4.96169	2.96503	-1.78277
H	3.67682	3.99762	-2.40112
H	1.42107	3.50316	3.38801
H	0.4937	3.54812	1.8849
H	1.51911	2.1601	2.24013
H	5.26823	-4.68142	-2.04191
H	4.96471	-2.96321	-1.78066
H	3.6796	-3.99452	-2.40065
H	1.41979	-3.5041	3.387
H	0.49224	-3.55338	1.88415
H	1.51446	-2.1625	2.23708
Pd	-0.21499	-0.00007	-1.30867
Ru	-3.88677	-0.00018	0.0946
C	-3.48874	0.72384	2.16791
C	-4.80535	1.15806	1.73671
C	-5.61064	-0.01169	1.51096
C	-4.78421	-1.1652	1.74363
C	-3.47557	-0.70482	2.17194
C	-7.0696	-0.02571	1.16159
H	-7.68176	-0.02685	2.07253
H	-7.35369	0.85252	0.5768
H	-7.33872	-0.91377	0.58466
C	-5.23772	-2.59499	1.7084
H	-6.03541	-2.74714	0.97719
H	-4.41736	-3.27207	1.45793
H	-5.62763	-2.89869	2.68845
C	-2.34464	-1.58441	2.61449
H	-2.32589	-2.52473	2.05778
H	-1.37819	-1.09531	2.47337
H	-2.44288	-1.83225	3.67929
C	-2.3733	1.62617	2.60386
H	-1.39834	1.15421	2.4628
H	-2.3731	2.5641	2.04285
H	-2.47379	1.87702	3.66773
C	-5.2855	2.57895	1.69417
H	-6.08453	2.71288	0.96084
H	-5.68284	2.87969	2.67214
H	-4.47762	3.27031	1.44211
H	-5.19514	-1.34514	-2.24096
H	-5.19515	1.34481	-2.24105

15-B

C	-3.93472	0.69942	-2.4599
C	-2.71397	1.15105	-1.79814
C	-3.93411	-0.71271	-2.45763
C	-2.71283	-1.1612	-1.79476

C	-1.97832	-0.00412	-1.38316
C	-2.36417	2.49055	-1.48268
C	-1.20915	2.90866	-0.84339
C	-0.83455	4.28823	-0.66538
C	0.43409	4.2952	-0.16722
C	0.84469	2.91883	-0.00941
C	2.21928	2.56779	0.32057
C	2.63605	1.32362	0.72459
C	3.9225	0.6908	0.60395
C	3.92408	-0.68402	0.60404
C	2.63909	-1.31979	0.72483
C	2.22514	-2.56505	0.32132
C	0.85113	-2.91943	-0.00752
C	0.44341	-4.2969	-0.1637
C	-0.82589	-4.29318	-0.66013
C	-1.20371	-2.91456	-0.83907
C	-2.36032	-2.4996	-1.47738
N	-0.17803	2.09077	-0.3904
N	-0.17375	-2.094	-0.38815
C	3.27818	-3.60891	0.07685
C	5.26476	-5.56174	-0.36975
C	4.86147	-5.22926	0.92702
C	3.88567	-4.25847	1.17502
C	6.34705	-6.58627	-0.60951
C	3.27001	3.61412	0.07664
C	3.87486	4.26551	1.17519
C	4.84847	5.23865	0.92783
C	5.25215	5.57176	-0.36865
C	6.33207	6.59894	-0.6077
H	-3.04309	3.27694	-1.80366
H	-1.45454	5.13696	-0.9226
H	1.05566	5.14714	0.06561
H	4.8059	1.27653	0.3746
H	4.80882	-1.26776	0.37477
H	1.06719	-5.14723	0.06912
H	-1.44441	-5.14348	-0.91566
H	-3.0382	-3.28773	-1.79625
H	6.17446	-7.14177	-1.5359
H	7.32902	-6.10534	-0.69781
H	6.4088	-7.30414	0.21298
H	5.30236	5.75089	1.77274
H	6.15856	7.15421	-1.53405
H	6.3917	7.31679	0.21496
H	7.31524	6.12041	-0.69566
C	4.64291	4.90766	-1.43854
C	3.65548	3.93789	-1.24452
H	4.94387	5.15123	-2.45475
C	3.6632	-3.93213	-1.24459
C	4.65285	-4.8995	-1.43925
H	4.95345	-5.14262	-2.45569
H	5.31744	-5.74012	1.77165
C	3.49407	-3.94664	2.60354
C	3.03792	-3.25394	-2.44347
C	3.4827	3.95312	2.60345
C	3.03305	3.25777	-2.44378
Se	1.42943	0.00059	1.38177
H	3.94187	-4.66764	3.29142
H	3.82841	-2.94899	2.90996
H	2.4086	-3.97713	2.74635
H	3.60025	-3.48097	-3.35231
H	2.00595	-3.58784	-2.6023
H	3.00796	-2.1654	-2.32922
H	3.92797	4.67549	3.29152
H	3.81933	2.95643	2.91049
H	2.39702	3.98084	2.7452
H	3.59559	3.48604	-3.35218
H	2.00035	3.58893	-2.6036
H	3.00584	2.16918	-2.32927
Pd	-0.30509	-0.00168	-0.32439
Ru	-4.04519	-0.00298	-0.32724
C	-4.23051	-0.68686	1.79739
C	-5.35213	-1.17278	1.01466
C	-6.09277	-0.035	0.53834
C	-5.4047	1.15026	0.97596
C	-4.26355	0.74128	1.77426
C	-7.39286	-0.0765	-0.20914
H	-7.475	-0.97299	-0.82842
H	-8.23706	-0.08228	0.49216
H	-7.51319	0.793	-0.85988
C	-5.87525	2.56245	0.78705

H	-6.54558	2.85634	1.60494
H	-5.04053	3.26766	0.77875
H	-6.42781	2.68192	-0.14817
C	-3.33729	1.67044	2.50066
H	-3.77812	1.98346	3.45574
H	-2.37922	1.19326	2.71785
H	-3.13458	2.57366	1.91935
C	-3.26588	-1.54925	2.55587
H	-3.69523	-1.85052	3.51993
H	-3.02086	-2.46093	2.00481
H	-2.331	-1.0225	2.75901
C	-5.7573	-2.61034	0.87168
H	-6.41243	-2.90853	1.70027
H	-6.30513	-2.78444	-0.05774
H	-4.89086	-3.27612	0.88344
H	-4.67781	1.33725	-2.92021
H	-4.67675	-1.35261	-2.91577

15-TS

C	-4.278745	0.708558	-2.193258
C	-2.95921	1.172202	-1.75114
C	-4.278736	-0.709107	-2.193161
C	-2.959174	-1.172682	-1.751039
C	-2.207574	-0.000215	-1.448035
C	-2.477309	2.498762	-1.576713
C	-1.204261	2.909655	-1.163241
C	-0.794617	4.28643	-1.047374
C	0.506782	4.291821	-0.643254
C	0.948089	2.918315	-0.492248
C	2.329947	2.640753	-0.074089
C	2.889257	1.368127	0.096255
C	4.129846	0.697421	0.461277
C	4.129873	-0.697591	0.461265
C	2.889289	-1.368303	0.096215
C	2.330066	-2.640977	-0.074139
C	0.948277	-2.918611	-0.492313
C	0.507033	-4.292109	-0.643349
C	-0.794382	-4.286796	-1.047425
C	-1.204094	-2.91004	-1.163232
C	-2.477207	-2.499206	-1.576606
N	-0.135507	2.096696	-0.815494
N	-0.135374	-2.097036	-0.815524
C	3.232378	-3.814327	0.187971
C	4.979012	-5.968104	0.694754
C	4.191792	-5.424990	1.714932
C	3.318385	-4.356678	1.488258
C	5.891806	-7.141396	0.958251
C	3.23231	3.814057	0.187883
C	3.314737	4.359773	1.48704
C	4.18209	5.433372	1.711682
C	4.970462	5.975309	0.691698
C	5.921912	7.114662	0.967018
H	-3.162311	3.308986	-1.815561
H	-1.423089	5.141134	-1.259997
H	1.134854	5.151028	-0.462205
H	5.01888	1.262611	0.718414
H	5.018923	-1.262775	0.718398
H	1.135241	-5.151201	-0.462311
H	-1.422836	-5.141513	-1.260053
H	-3.162185	-3.309466	-1.815399
H	6.775025	-7.11439	0.313659
H	6.22876	-7.161472	1.998452
H	5.376678	-8.090182	0.76394
H	4.241442	5.857712	2.711128
H	6.051518	7.749999	0.086239
H	5.569269	7.741175	1.791127
H	6.914012	6.738287	1.245226
C	4.869136	5.416093	-0.586173
C	4.016994	4.341923	-0.860550
H	5.468816	5.8272	-1.39476
C	4.0209	-4.339075	-0.859221
C	4.879101	-5.407834	-0.582819
H	5.487097	-5.811775	-1.388811
H	4.259163	-5.842072	2.716914
C	2.489018	-3.807789	2.627895
C	3.951647	-3.771976	-2.259724
C	2.482016	3.813952	2.6257
C	3.944301	3.777633	-2.262009
Se	1.765982	-0.000107	-0.240898

H	2.673889	-4.368008	3.547389
H	2.718127	-2.754945	2.827847
H	1.416263	-3.865341	2.411251
H	4.611173	-4.322556	-2.934407
H	2.936315	-3.825052	-2.668436
H	4.251732	-2.71841	-2.287984
H	2.66593	4.375152	3.544788
H	2.709445	2.761112	2.827505
H	1.409726	3.872551	2.406969
H	4.602436	4.329343	-2.937126
H	2.928105	3.831305	-2.66852
H	4.24442	2.724157	-2.29264
Pd	-0.383557	-0.000164	-0.859166
Ru	-4.067508	-0.000079	-0.070581
C	-3.977449	-0.711405	2.053075
C	-5.201575	-1.16219	1.411221
C	-5.973756	-0.001203	1.054576
C	-5.203864	1.161779	1.409684
C	-3.978898	0.714232	2.05218
C	-7.360297	-0.002946	0.482131
H	-7.542134	-0.886821	-0.133984
H	-8.106249	-0.003105	1.287058
H	-7.543847	0.879709	-0.135222
C	-5.656912	2.587584	1.29937
H	-6.184248	2.894354	2.211816
H	-4.813349	3.268832	1.163338
H	-6.343483	2.728774	0.461117
C	-2.94206	1.610002	2.661453
H	-3.228904	1.887329	3.684019
H	-1.966743	1.120914	2.710745
H	-2.823517	2.534659	2.091082
C	-2.938863	-1.604326	2.663557
H	-3.225497	-1.881469	3.686231
H	-2.817963	-2.529171	2.093981
H	-1.964687	-1.112969	2.712843
C	-5.651824	-2.589025	1.302811
H	-6.178518	-2.895598	2.215695
H	-6.338162	-2.732681	0.464787
H	-4.806929	-3.268795	1.167657
H	-5.089874	1.338309	-2.534907
H	-5.089846	-1.338921	-2.53474

7. References

[1] White, C. J.; Wang, T.; Jacobson, R. A.; Angelici, R. J. Synthesis, equilibrium binding, and ^{77}Se NMR studies of η^1-selenophene (Seln) complexes: [CpRu(CO)(PPh_3)(η^1(Se)-Seln)]BF_4. *Organometallics* **1994**,*13,* 4474–4480*.*

[2] White, C. J.; Angelici, R. J. Synthesis, reactions, and ^{77}Se NMR studies of η^5-selenophene complexes of chromium. *Organometallics* **1995**, *14*, 332–340.

[3] M. J. Frisch, G. W. Trucks, H. B. Schlegel, G. E. Scuseria, M. A. Robb, J. R. Cheeseman, G. Scalmani, V. Barone, G. A. Petersson, H. Nakatsuji, X. Li, M. Caricato, A. V. Marenich, J. Bloino, B. G. Janesko, R. Gomperts, B. Mennucci, H. P. Hratchian, J. V. Ortiz, A. F. Izmaylov, J. L. Sonnenberg, D. Williams-Young, F. Ding, F. Lipparini, F. Egidi, J. Goings, B. Peng, A. Petrone, T. Henderson, D. Ranasinghe, V. G. Zakrzewski, J. Gao, N. Rega, G. Zheng, W. Liang, M. Hada, M. Ehara, K. Toyota, R. Fukuda, J. Hasegawa, M. Ishida, T. Nakajima, Y. Honda, O. Kitao, H. Nakai, T. Vreven, K. Throssell, J. A. Montgomery, Jr., J. E. Peralta, F. Ogliaro, M. J. Bearpark, J. J. Heyd, E. N. Brothers, K. N. Kudin, V. N. Staroverov, T. A. Keith, R. Kobayashi, J. Normand, K. Raghavachari, A. P. Rendell, J. C. Burant, S. S. Iyengar, J. Tomasi, M. Cossi, J. M. Millam, M. Klene, C. Adamo, R. Cammi, J. W. Ochterski, R. L. Martin, K. Morokuma, O. Farkas, J. B. Foresman, and D. J. Fox, Gaussian 16, Revision C.01; Gaussian, Inc.: Wallingford CT, **2016**.

[4] C. Lee, W. Yang, R. G. Parr, Development of the Colle-Salvetti correlation-energy formula into a functional of the electron density. *Phys. Rev. B* **1988**, *37*, 785–789.

[5] A. D. Becke, Density-functional exchange-energy approximation with correct asymptotic behavior. *Phys. Rev. A* **1988**, *38*, 3098–3100.

[6] Z. Chen, C. S. Wannere, C. Corminboeuf, R. Puchta, P. v. R. Schleyer, Nucleus-Independent Chemical Shifts (NICS) as an Aromaticity Criterion. *Chem. Rev.* **2005**, *105*, 3842–3888.

[7] Z. Wang, py.Aroma: An Intuitive Graphical User Interface for Diverse Aromaticity Analyses, *Chemistry*, **2024**, *6*, 1692–1703.

[8] D. W. Szczepanik, https://aromaticity.eu/eddb/

[9] D. W. Szczepanik, M. Andrzejak, K. Dyduch, Emil Żak, M. Makowski, G. Mazur, J. Mrozek, A uniform approach to the description of multicenter bonding. *Phys. Chem. Chem. Phys.,* **2014**, *16*, 20514–20523.

[10] D. W. Szczepanik, M. Andrzejak, J. Dominikowska, B. Pawełek, T. M. Krygowski, H. Szatylowicz, M. Solà, The electron density of delocalized bonds (EDDB) applied for quantifying aromaticity. *Phys. Chem. Chem. Phys.* **2017**, *19*, 28970–28981.

[11] Rigaku Oxford Diffraction, CrysAlis PRO Software system, version 1.171.41.80a, Rigaku Oxford Diffraction, **2020**.

[12] G. M. Sheldrick, SHELXT-Integrated Space-Group and Crystal-Structure Determination, *Acta Crystallogr., Sect. A,* **2015**, *A71*, 3–8.

[13] G. M. Sheldrick, Crystal structure refinement with *SHELXL*, *Acta Crystallogr., Sect. C,* **2015**, *C71*, 3–8.

[14] Berlicka, A.; Foryś-Martowłos, P.; Białek, M. J.; Stasiak, K.; Walczak, A.; Wójcik, A.; Białońska, A.; Latos-Grażyński, L. 21-Carba-23-selenaporphyrinoid dyads—an azepine unit as a merging motif. *Angew. Chem. Int. Ed.* **2024**, *63*, e202314925.

[15] Cuesta, L.; Karnas, E.; Lynch, V. M.; Sessler, J. L.; Kajonkijya, W.; Zhu, W.; Zhang, M.; Ou, Z.; Kadish, K. M.; Ohkubo, K.; Fukuzumi, S. (Pentaniethylcyclopentadienyl) ruthenium π-complexes of metalloporphyrins: Platforms with novel photo- and electrochemical properties. *Chem. Eur. J.* **2008**, *14*, 10206–10210.

[16] Cuesta, L.; Karnas, E.; Lynch,V. M.; Chen, P.; Shen, J.; Kadish, K. M.; Ohkubo, K.; Fukuzumi, S.; Sessler, J. L. Metalloporphycenes: synthesis and characterization of (pentamethylcyclopentadienyl) ruthenium sitting-atop and π-complexes. *J. Am. Chem. Soc.* **2009**, *131*, 13538–13547.

8. Author Contributions

A. Berlicka: synthesis of **11**-**15**, (supporting), data analysis (leading), NMR and UV/Vis measurements (leading), preparation of Supporting Information (leading), writing of an original draft (equal), project administration.
A. Walczak: synthesis of **11**-**15** (leading), NMR and UV/Vis measurements (supporting), data analysis (supporting).
M. J. Białek: DFT modeling; analysis and corrections to a prepared manuscript (supporting), funding acquisition.
K. Ślepokura: X-ray analysis of **11** and **13**, i.e. diffraction data collection; crystal structure determination and deposition of the CIF file at the CCDC; preparation of the crystallographic experimental part in the Supporting Information (leading).
P.J. Chmielewski: separation of stereoisomers **15-A** and **15-B**.
L. Latos-Grażyński: data analysis (supporting), writing of original draft (equal), funding acquisition.
